# Dissecting cell identity via network inference and in silico gene perturbation

**DOI:** 10.1038/s41586-022-05688-9

**Published:** 2023-02-08

**Authors:** Kenji Kamimoto, Blerta Stringa, Christy M. Hoffmann, Kunal Jindal, Lilianna Solnica-Krezel, Samantha A. Morris

**Affiliations:** 1 Department of Developmental Biology, Washington University School of Medicine in St Louis, St Louis, MO USA; 2 Department of Genetics, Washington University School of Medicine in St Louis, St Louis, MO USA; 3 Center of Regenerative Medicine, Washington University School of Medicine in St Louis, St Louis, MO USA

**Keywords:** Gene regulatory networks, Developmental biology

## Abstract

Cell identity is governed by the complex regulation of gene expression, represented as gene-regulatory networks^1^. Here we use gene-regulatory networks inferred from single-cell multi-omics data to perform in silico transcription factor perturbations, simulating the consequent changes in cell identity using only unperturbed wild-type data. We apply this machine-learning-based approach, CellOracle, to well-established paradigms—mouse and human haematopoiesis, and zebrafish embryogenesis—and we correctly model reported changes in phenotype that occur as a result of transcription factor perturbation. Through systematic in silico transcription factor perturbation in the developing zebrafish, we simulate and experimentally validate a previously unreported phenotype that results from the loss of *noto*, an established notochord regulator. Furthermore, we identify an axial mesoderm regulator, *lhx1a*. Together, these results show that CellOracle can be used to analyse the regulation of cell identity by transcription factors, and can provide mechanistic insights into development and differentiation.

## Abstract (web summary)

A machine-learning-based strategy called CellOracle combines computational perturbation with modelling of gene-regulatory networks to analyse how cell identity is regulated by transcription factors, and correctly predicts phenotypic changes after transcription factor perturbation in the developing zebrafish.

## Main

The expansion of single-cell technologies into perturbational omics is enabling the development of methods to characterize cell identity. For example, single-cell RNA sequencing (scRNA-seq) coupled with pooled CRISPR screens offers much promise for analysing the genetic regulation of cell identity^2–4^, but cannot be readily used in many biological contexts. Computational methods to simulate single-cell phenotypes after perturbation are emerging, although many approaches still require experimental perturbation data for model training, and thus their scale and application are limited^5^. Moreover, previous deep-learning-based models represent a ‘black box’, which restricts the interpretation of gene-regulatory mechanisms that underlie the simulated biological events. In this respect, gene-regulatory network (GRN) modelling approaches are promising as they reconstruct systematic gene–gene associations from unperturbed single-cell omics data^6–11^. However, previous methods for analysing GRNs largely focus on the static network structure, and determining how a static GRN governs cell identity during dynamic biological processes therefore remains a challenge. Scalable and interpretable approaches are required to understand how gene-regulatory mechanisms relate to observed complex single-cell phenotypes.

Here we present a strategy that overcomes these limitations by combining computational perturbation with GRN modelling. CellOracle integrates multimodal data to build custom GRN models that are specifically designed to simulate shifts in cell identity following transcription factor (TF) perturbation, providing a systematic and intuitive interpretation of context-dependent TF function in regulating cell identity. We apply CellOracle to well-characterized biological systems: haematopoiesis in mice and humans; and the differentiation of axial mesoderm into notochord and prechordal plate in zebrafish. In haematopoiesis, we show that CellOracle recapitulates well-known cell fate regulation governed by TFs. Furthermore, we apply CellOracle to systematically perturb TFs across zebrafish development, recovering known and putative regulators of cell identity. Focusing on axial mesoderm, we predict and validate a prechordal plate phenotype after loss of function (LOF) of the prototypical notochord regulator, *noto*. Moreover, we also simulate and validate a role for the TF *lhx1a* in the development of axial mesoderm. Together, these results show that CellOracle can be used to infer and interpret cell-type-specific GRN configurations at high resolution, enabling mechanistic insights into the regulation of cell identity. CellOracle code and documentation are available at https://github.com/morris-lab/CellOracle and data can be explored at https://celloracle.org.

## In silico gene perturbation using CellOracle

**Fig. 1:**
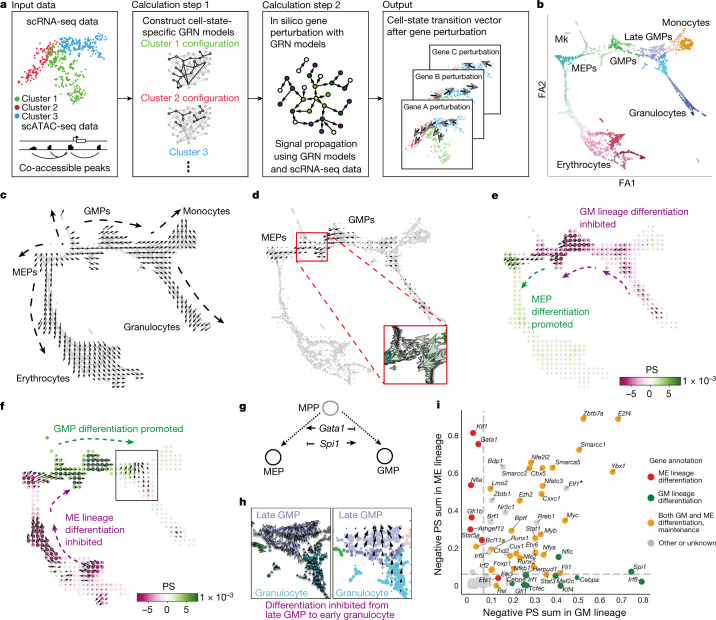
Overview of CellOracle and application to haematopoiesis. **a**, Simulation of cell-state transitions in response to TF perturbation. First, CellOracle constructs custom transcriptional GRNs using scRNA-seq and scATAC-seq data (left). Accessible promoter and enhancer peaks from scATAC-seq data are then combined with scRNA-seq data to generate cluster-specific GRN models (middle). CellOracle simulates the change in cell state in response to a TF perturbation, projecting the results onto the cell trajectory map (right). **b**, Force-directed graph of 2,730 myeloid progenitor cells from Paul et al.^16^. Twenty-four cell clusters (Louvain clustering) were organized into six main cell types. Mk, megakaryocytes. **c**, Differentiation vectors for each cell projected onto the force-directed graph. **d**, CellOracle simulation of cell-state transition in *Spi1* KO simulation. Summarized cell-state transition vectors projected onto the force-directed graph. Vectors for each cell are shown in the inset. **e**, *Spi1* KO simulation vector field with perturbation scores (PSs). **f**, *Gata1* KO simulation with perturbation scores. **g**, Schematic of *Spi1*–*Gata1* lineage switching. MPP, multipotent progenitor. **h**, Detail of *Gata1* simulation for the granulocyte branch. Left, cell-state transition vectors for each cell. Right, summarized vectors. **i**, Systematic KO simulation result of 90 TFs in the GM and ME lineage is summarized as a scatter plot of the sum of negative perturbation scores (shown in log scale). Dashed lines represent cut-off values corresponding to false-positive rate (FPR) = 0.01. Genes are classified into four categories on the basis of their previously reported functions (Supplementary Table 2). The asterisk refers to Supplementary Fig. 11, where we expand on the predicted phenotype. All scores can be explored through our web application (https://celloracle.org).

**Extended Data Fig. 1:**
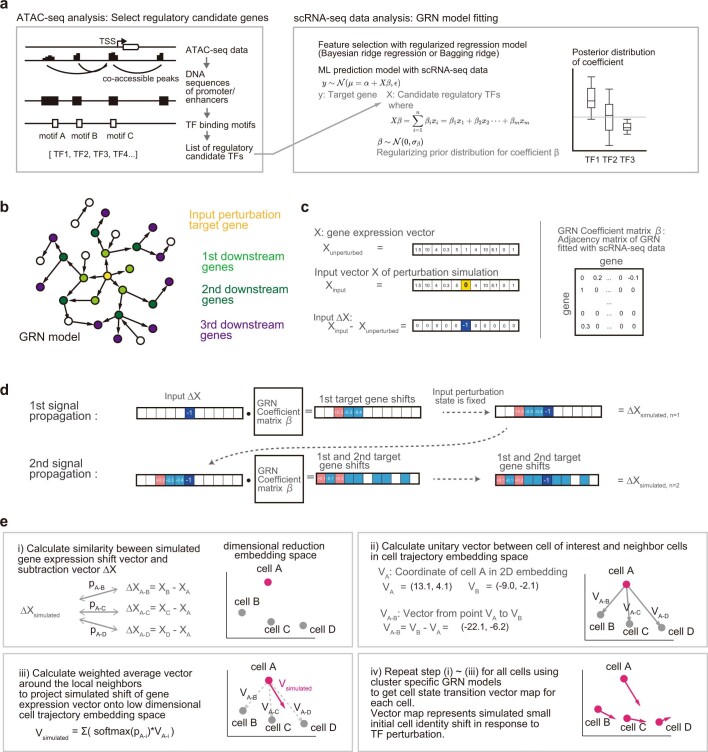
Overview of the CellOracle workflow. **(a)** Overview of the CellOracle context-dependent GRN model construction method. First, genomic DNA sequence and TF-binding-motif information provide all potential regulatory links to construct a ‘base GRN.’ CellOracle uses scATAC-seq data to identify accessible promoter and enhancer DNA sequences in this step. The DNA sequence of these regulatory elements is scanned for TF-binding motifs, generating a list of potential regulatory connections between a TF and its target genes (left). Next, active connections (described below), dependent on cell state or cell type, are identified from all potential connections in the base GRN. CellOracle builds machine-learning (ML) models for this step that predict the quantitative relationship between the TF and the target gene. The ML model fitting results present the certainty of connection as a distribution, enabling the identification of GRN configurations by removing inactive connections from the base GRN structure. **(b—d)** Overview of signal propagation simulation. CellOracle leverages an inferred GRN model to simulate how target gene expression changes in response to the changes in regulatory gene expression. **(b)** The input TF perturbation (shown in yellow) is propagated side-by-side within the network model. **(c)** Input data and GRN coefficient matrix format used in the signal propagation calculation. **(d)** Leveraging the linear predictive ML algorithm features, CellOracle uses the GRN model as a function to perform the signal propagation calculation. Iterative matrix multiplication steps enable the estimation of indirect and global downstream effects resulting from the perturbation of a single TF. **(e)** After signal propagation, the simulated gene expression shift vector is converted into a 2D vector and projected onto the low-dimensional space. Details are described in the Methods.

To gain mechanistic insight into the regulation of cell identity, we developed an in silico strategy to simulate changes in cell identity upon TF perturbation. CellOracle uses custom GRN modelling (Extended Data Fig. 1a) to simulate global downstream shifts in gene expression following knockout (KO) or overexpression of TFs. These simulated values are converted into a vector map of transitions in cell identity, which enables simulated changes in cell identity to be intuitively visualized within a low-dimension space (Fig. 1a and Methods). In silico perturbation involves four steps. (1) Cell-type- or cell-state-specific GRN configurations are constructed using cluster-wise regularized linear regression models with multi-omics data. (2) Using these GRN models, shifts in target gene expression in response to TF perturbation are calculated. This step applies the GRN model as a function to propagate the shift in gene expression rather than the absolute gene expression value, representing the signal flow from TF to target gene. This signal is propagated iteratively to calculate the broad, downstream effects of TF perturbation, allowing the global transcriptional ‘shift’ to be estimated (Extended Data Fig. 1b–d). (3) The cell-identity transition probability is estimated by comparing this shift in gene expression to the gene expression of local neighbours. (4) The transition probability is converted into a weighted local average vector to represent the simulated directionality of cell-state transition for each cell following perturbation of candidate TFs. In the final calculation step, the multi-dimensional gene expression shift vector is reduced to a two-dimensional (2D) vector, allowing for more robust predictions against noise (Extended Data Fig. 1e). We purposefully limit the simulation output data to a 2D vector representing the predicted shift in cell identity because our goal is to model changes in identity rather than predicting absolute changes in gene expression levels. Further details of the CellOracle algorithm are provided in the Methods, including validation of the range of simulated values; null or randomized model analysis; and hyperparameter evaluation (Supplementary Figs. 2–10).

## GRN inference and benchmarking with CellOracle

The CellOracle GRN model must represent regulatory connections as a directed network edge to support signal propagation in response to TF perturbation. Thus, we developed a custom GRN modelling method motivated by previous approaches that incorporate promoter and TF-binding information with scRNA-seq data to infer a directional GRN^7^ (Extended Data Fig. 1a and Methods). First, using single-cell chromatin accessibility data (single-cell assay for transposase-accessible chromatin using sequencing; scATAC-seq), we incorporate flexible promoter and enhancer regions, encompassing proximal and distal regulatory elements. This initial step uses the transcriptional start site (TSS) database (http://homer.ucsd.edu/) and Cicero, an algorithm that identifies co-accessible scATAC-seq peaks, to distinguish accessible promoters and enhancers^12^. The DNA sequence of these elements is then scanned for TF-binding motifs, generating a ‘base GRN structure’ of all potential regulatory interactions in the species of interest (Extended Data Fig. 1a, left). This process is beneficial as it narrows the scope of possible regulatory candidate genes before model fitting (below) and helps define the directionality of regulatory edges in the GRN. To support GRN inference without requiring sample-specific scATAC-seq datasets, we have assembled a base GRN from a mouse scATAC-seq atlas^13^. We have also created general promoter base GRNs for ten commonly studied species (Supplementary Table 1 and Methods). These base GRNs are built into the CellOracle library and provide an alternative solution when scATAC-seq data are unavailable.

In the second step of CellOracle GRN inference, we use scRNA-seq data to identify active connections in the base GRN, generating cell-type- or cell-state-specific GRN configurations for each cluster. In this step, we build a machine-learning model to predict the expression of target genes on the basis of TF expression (Extended Data Fig. 1a, right). Because CellOracle uses genomic sequences and information on TF-binding motifs to infer the base GRN structure and directionality, it does not need to infer the causality or directionality of the GRN from expression data. This approach allows CellOracle to adopt a relatively simple modelling method for GRN inference—a regularized linear machine-learning model. Crucially, this strategy enables the above signal propagation to simulate TF perturbation. To support the use of a linear model, the gene expression matrix of scRNA-seq data is divided into several clusters in advance so that a single data unit for each fitting process represents a linear relationship rather than non-linear or mixed regulatory relationships. Furthermore, a Bayesian or bagging strategy enables the certainty of connection to be presented as a distribution; this allows weak or insignificant connections to be removed from the base GRN (Extended Data Fig. 1a, right), producing a cell-type- or cell-state-specific GRN configuration.

**Extended Data Fig. 2:**
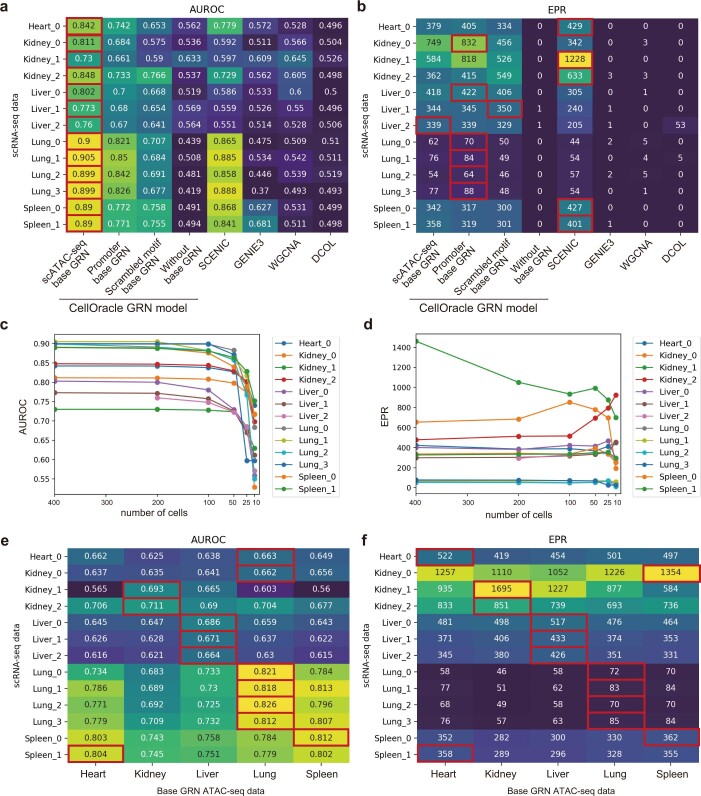
Benchmarking of inferred GRN configurations. **(a,b)** We benchmarked the CellOracle GRN modelling method against pre-existing GRN inference algorithms: WGCNA, DCOL, GENIE3, and SCENIC. Details of input data and ground-truth data are described in the Methods. We generated a base GRN using the Cusanovich mouse sci-ATAC-seq atlas dataset^13^ or UCSC mm9 promoter DNA sequence data. CellOracle scored better than or comparable to other algorithms. CellOracle results with a promoter base GRN received lower but comparable scores than the scATAC-seq base GRN results. In addition, we tested the CellOracle GRN method using two impaired base GRN datasets (Scrambled motif base GRN and no base GRN) to investigate how the base GRN data contributes to its performance. **(a)** AUROC (Area Under the Receiver Operating Characteristic curve) heat map. The top score in each condition is highlighted with a red rectangle. **(b)** EPR (Early Precision Ratio) heat map. EPR represents the EP ratio relative to the random model ER score. An EPR of less than 1 indicates that the GRN inference results are no better than random prediction. **(c,d)** The performance of CellOracle was tested after downsampling cells. GRN models were made after downsampling to 400, 200, 100, 50, 25, and 10 cells. We recommend at least 50 cells for GRN inference based on these results. CellOracle used the same mouse scATAC-seq base GRN as **a** and **b**. The Liver_2 sample contains less than 400 cells. **(e,f)** GRN inference performance comparison between different base GRN data generated from various tissue types. The top score in each condition is highlighted with a red rectangle.

To benchmark our GRN inference method, we generated a comprehensive transcriptional ground-truth GRN using 1,298 chromatin immunoprecipitation followed by sequencing (ChIP–seq) datasets for 80 regulatory factors across 5 different tissues^14^. In addition to benchmarking against diverse GRN inference algorithms, we also assessed the performance of our approach using different base GRNs, data sources and cell downsampling (Extended Data Fig. 2). Inference performance as assessed by the area under the receiver operating characteristic (AUROC) ranged from 0.66 to 0.85 for the promoter base GRN and 0.73 to 0.91 for the scATAC-seq base GRN. Altogether, this benchmarking demonstrates the accuracy of our transcriptional GRN modelling method with a diverse range of data sources. Combined with our signal propagation strategy, CellOracle can effectively interrogate network biology and cell-identity dynamics through in silico perturbation.

## GRN analysis and TF KO in haematopoiesis

**Extended Data Fig. 3:**
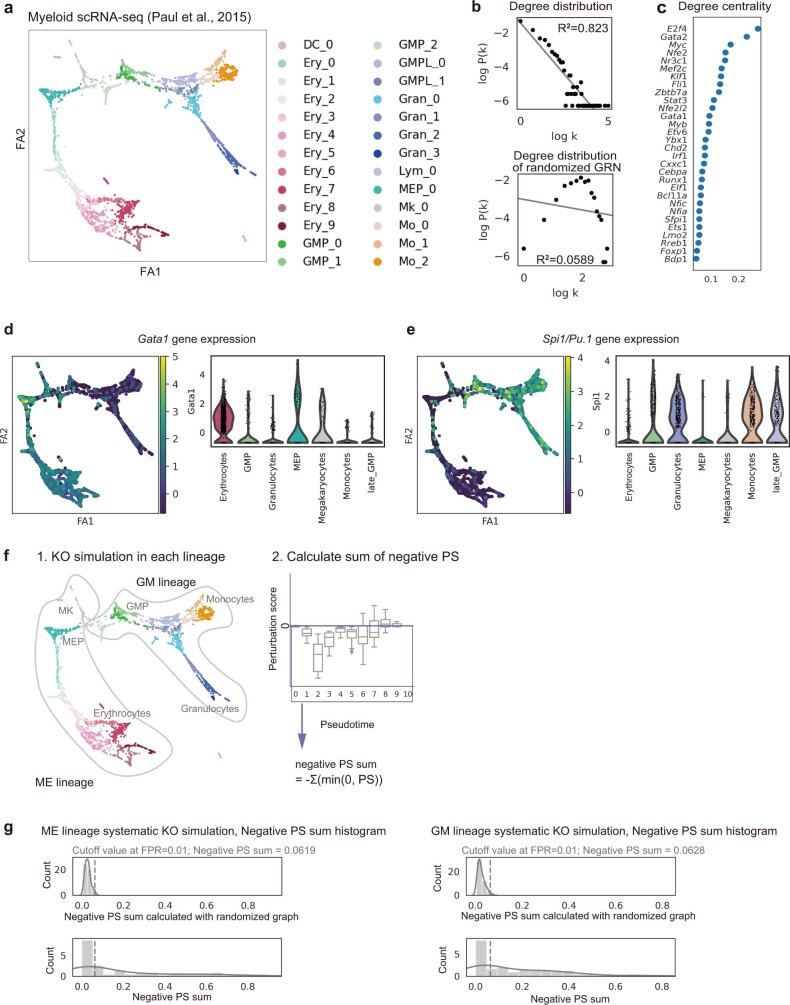
CellOracle analysis of Paul et al. haematopoiesis data. **(a)** Force-directed graph of 2,730 myeloid progenitor cells from Paul et al.^16^ with all clusters labelled. DC = Dendritic Cell; Ery = Erythrocyte; GMP = Granulocyte–Monocyte Progenitor; Gran = Granulocyte; Lym = Lymphoid; MEP = Megakaryocyte–Erythrocyte Progenitor; Mk = Megakaryocyte; Mo = Monocyte. We removed the DC and Lymphoid cell clusters to focus on myeloid cell differentiation. **(b)** Degree distribution of the MEP_0 cluster GRN model. After making the GRN model for each cluster, network edges were pruned. Then, we counted the network degree (k), representing the number of network edges for each gene. P(k) is the frequency of network degree k. The relationship between k and P(k) was visualized after log transformation to test whether the data follow a power law, in which there is a linear relationship between log(k) and log(P(k)). The R-squared value (R^2^) was calculated to quantify the degree of the linear relationship. The same analysis was performed on the randomized GRN (lower panel). **(c)** Top 30 genes ranked by degree centrality in the MEP_0 cluster GRN. **(d)** *Gata1* gene expression (log-transformed UMI) projected onto the force-directed graph (left) and violin plot grouped by cell-type annotation (right). **(e)** *Spi1* gene expression (log-transformed UMI) projected onto the force-directed graph (left) and violin plot grouped by cell-type annotation (right). **(f)** Systematic KO simulation of TFs in the GM (Granulocyte–Monocyte) and ME (Megakaryocyte–Erythrocyte) lineages. The sum of the negative perturbation scores is calculated for each TF to quantify the perturbation effect along each lineage. **(g)** Negative PS sum cut-off value calculation. Cut-off values were calculated for GM and ME lineage simulations based on the distribution of PS sum score calculated from the randomized simulation result (false-positive rate (FPR) = 0.01).

For validation, we aimed to reproduce known TF regulation of mouse haematopoiesis, a well-characterized differentiation paradigm^15^, by applying CellOracle to a 2,730-cell scRNA-seq atlas of myeloid progenitor differentiation^16^ (Fig. 1b and Extended Data Fig. 3a). We constructed GRN models for each of the 24 myeloid clusters identified, representing megakaryocyte and erythroid progenitors (MEPs) and granulocyte–monocyte progenitors (GMPs), differentiating toward erythrocytes, megakaryocytes, monocytes and granulocytes (Fig. 1c). To test whether the CellOracle simulation could recapitulate known TF regulation of cell identity, we performed in silico gene perturbation using the inferred GRNs, and compared the CellOracle KO simulation results with previous biological knowledge and ground-truth KO data.

**Extended Data Fig. 4:**
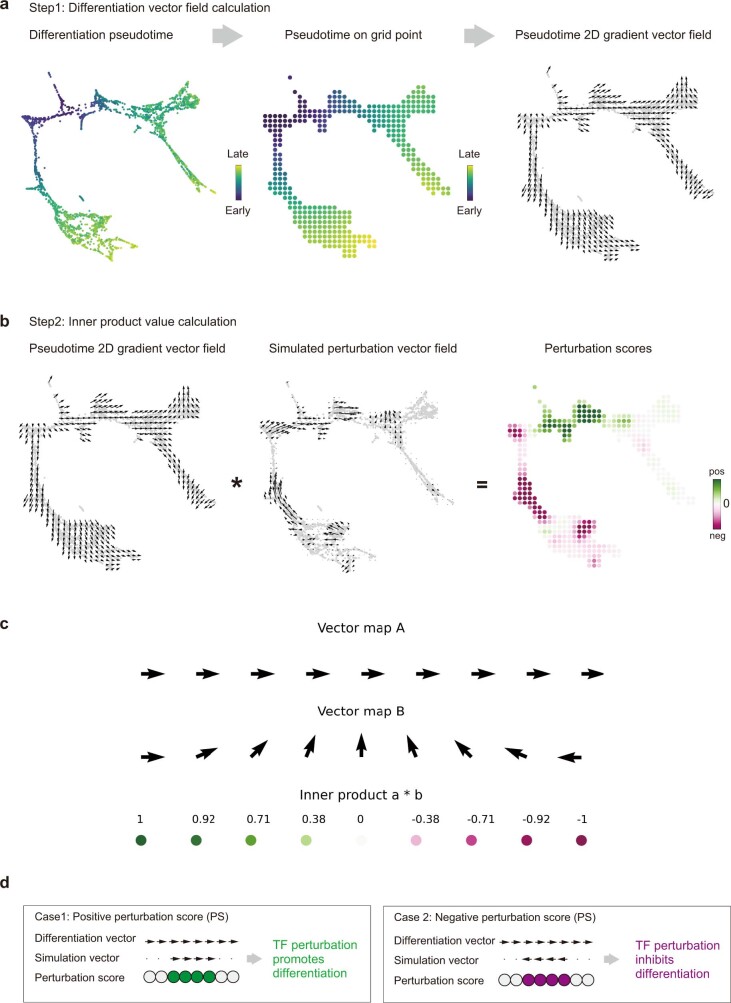
Perturbation score calculation and interpretation. **(a—d)** Schematic for perturbation score (PS) calculation. CellOracle calculates a PS by comparing the direction of the simulated cell state transition with the direction of cell differentiation. **(a)** Schematic for differentiation vector calculations. First, the pseudotime data are summarized by grid points. Then, CellOracle calculates a 2D gradient vector of the pseudotime data representing the directionality of differentiation pseudotime. **(b)** Calculation of the inner-product value between the differentiation vector and gene perturbation vectors. First, the results of the perturbation simulation are converted into the same vector field format as the differentiation vector field, and the inner product of these vectors is calculated to produce a PS. **(c)** A positive PS (magenta) suggests the perturbation vector and differentiation vector share a similar direction, thus, suggesting the TF perturbation would promote differentiation. In contrast, a negative PS (green) represents inhibited differentiation. **(d)** Schematic for perturbation score interpretation. A positive perturbation score (green) predicts that the perturbation promotes differentiation. A negative perturbation score (purple) represents inhibited differentiation.

First, *Spi1* (also known as *PU.1*) and *Gata1* KO simulation is used to illustrate the CellOracle in silico perturbation analysis. The TF perturbation simulation is visualized as a vector map on the 2D trajectory space (Fig. 1d and Supplementary Video 1), representing a potential shift in cell identity in response to TF perturbation. To enable the simulation results to be assessed systematically and objectively, we also devised a ‘perturbation score’ metric, which compares the directionality of the perturbation vector to the natural differentiation vector (Extended Data Fig. 4). A negative perturbation score suggests that TF KO delays or blocks differentiation (Extended Data Fig. 4b–d, purple). Conversely, a positive perturbation score suggests that the differentiation and KO simulation vectors share the same direction, indicating that loss of TF function promotes differentiation (Extended Data Fig. 4b–d, green). *Spi1* KO simulation yielded positive perturbation scores for MEPs, whereas GMPs had negative perturbation scores (Fig. 1e), suggesting that *Spi1* KO inhibits GMP differentiation and promotes MEP differentiation. Inverse perturbation score distributions were produced for the *Gata1* KO simulation (Fig. 1f). Comparing these predictions to previous reports^17,18^: PU.1 directs commitment to the neutrophil and monocyte lineages^19,20^, whereas GATA1 promotes the differentiation of erythroid cells^21^ and eosinophil granulocytes^22–24^. Overall, CellOracle accurately simulated the myeloid lineage switching governed by *Gata1* and *Spi1* (refs. ^15,25–27^; Fig. 1g), including a relatively mild *Gata1* KO phenotype in early granulocyte differentiation (Fig. 1h), which cannot be inferred from the low levels of *Gata1* expression in granulocytes (Extended Data Fig. 3d). However, CellOracle did not detect a previously reported depletion of erythrocyte progenitors after *Spi1* KO^27,28^, probably owing to changes in cell proliferation that are not predicted by the method.

**Extended Data Fig. 5:**
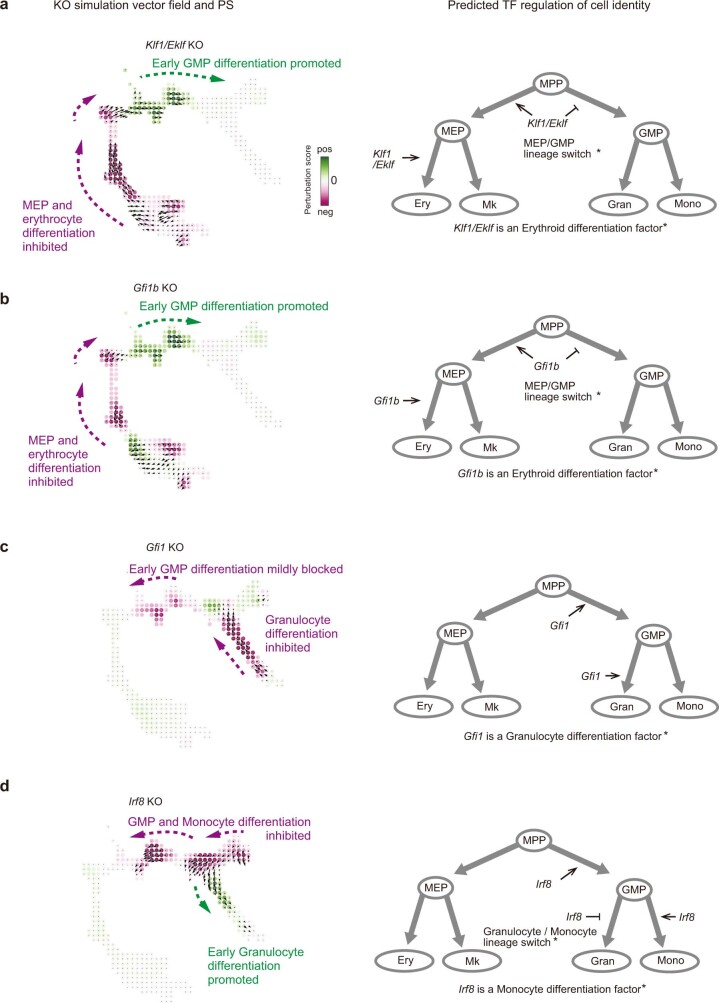
CellOracle TF KO simulation results for Paul et al. haematopoiesis data: part 1. **a**–**d**, CellOracle KO simulation for four key TF regulators of haematopoiesis: *Klf1* (**a**), *Gfi1b* (**b**), *Gfi1* (**c**) and *Irf8* (**d**) reported in^15,29^. The simulated cell state transition vector field is visualized with perturbation scores (PS; magenta: negative score; green: positive score). The right column shows a summary of the TF role based on the CellOracle simulation results, cell transition vector, and PS. For example, a positive PS in the TF KO simulation (green) implies that the TF has a role in cell state maintenance or inhibiting cell differentiation. In contrast, a negative PS in the KO simulation (magenta) implies that the TF normally promotes cell differentiation.

**Extended Data Fig. 6:**
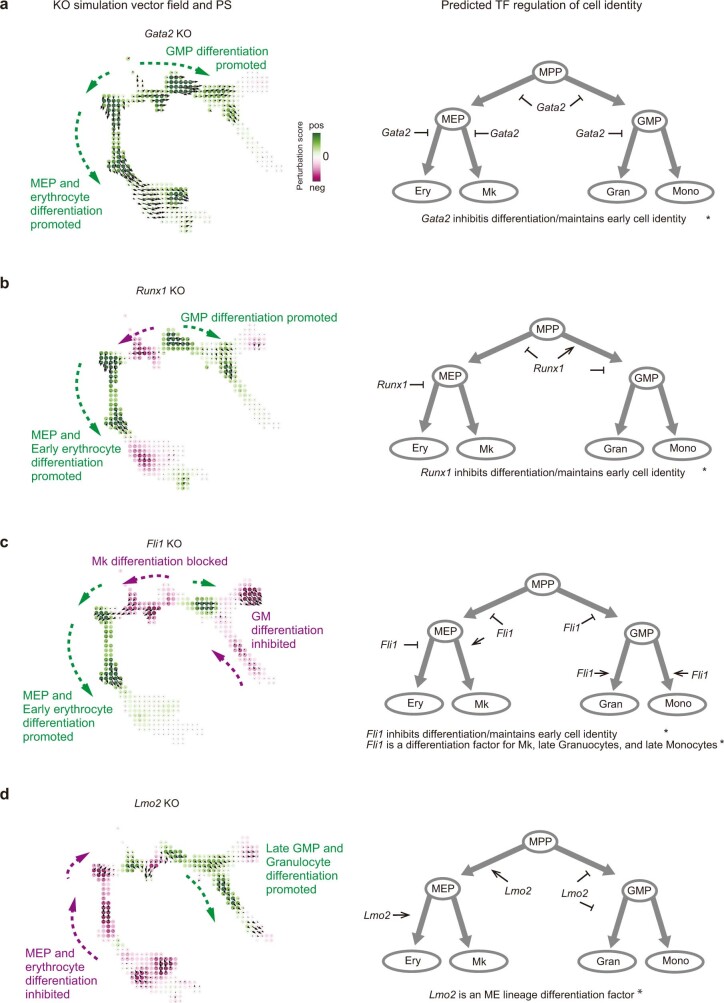
CellOracle TF KO simulation results for Paul et al. haematopoiesis data: part 2. **a**–**d**, CellOracle KO simulation results for *Gata2* (**a**), *Runx1* (**b**), *Fli1* (**c**) and *Lmo2* (**d**). The simulated cell state transition vector field is visualized with perturbation scores (PS; magenta: negative score; green: positive score). The right column shows a summary of the TF role based on the CellOracle simulation results, cell transition vector, and PS. For example, a positive PS in the TF KO simulation (green) implies that the TF has a role in cell state maintenance or inhibiting cell differentiation. In contrast, a negative PS in the KO simulation (magenta) implies that the TF normally promotes cell differentiation.

**Extended Data Fig. 7:**
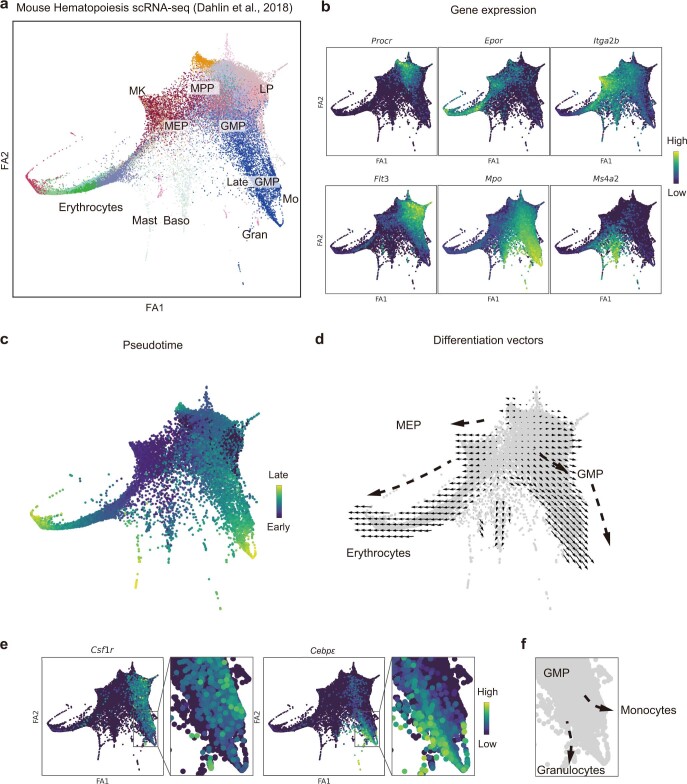
Dahlin et al. mouse haematopoiesis scRNA-seq data. **a**, Force-directed graph of 44,082 myeloid progenitor cells from Dahlin et al.^58^ with all clusters labelled. MPP = Multipotent Progenitor; GMP = Granulocyte–Monocyte Progenitor; Gran = Granulocyte; LP = Lymphoid progenitor; MEP = Megakaryocyte–Erythrocyte Progenitor; Mk = Megakaryocyte; Mo = Monocyte; Baso = Basophil. **(b)** Marker gene expression (log-transformed UMI) projected onto the force-directed graph. *Procr* = MPP marker; *Epor* = Erythrocyte marker; *Itga2b* = Mk marker; *Flt3* = LP marker; *Mpo* = Gran/Mo marker; *Ms4a2* = Baso marker. **(c)** Pseudotime values projected onto the force-directed graph. **(d)** Differentiation vector calculated from the pseudotime gradient. ME and GM lineages are highlighted. **(e)** *Csf1r* and *Cebpε* gene expression projected onto the force-directed graph. The right panel is a magnified area of the GM lineage. *Csf1r* is a monocyte marker, and *Cebpε* is a granulocyte marker. **(f)** Early lineage bifurcation between monocytes and granulocytes is observed on the force-directed graph.

**Extended Data Fig. 8:**
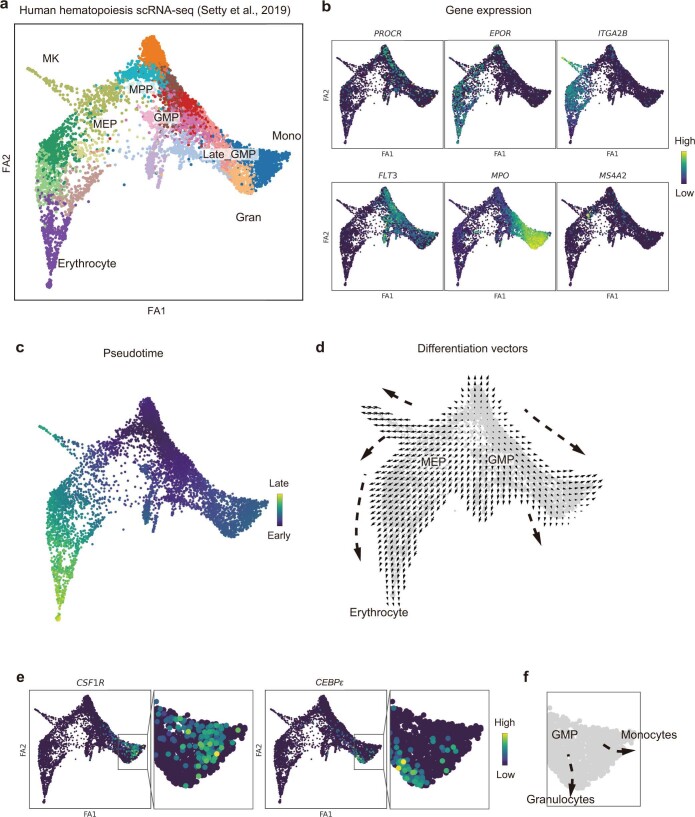
Setty et al. human haematopoiesis scRNA-seq data. **(a)** Force-directed graph of 5,610 myeloid progenitor cells from Setty et al.^67^ with all clusters labelled. MPP = Multipotent Progenitor; GMP = Granulocyte–Monocyte Progenitor; Gran = Granulocyte; MEP = Megakaryocyte–Erythrocyte Progenitor; Mk = Megakaryocyte. **(b)** Marker gene expression (log-transformed UMI) projected onto the force-directed graph. *PROCR* = MPP marker; *EPOR* = Erythrocyte marker; *ITGA2B* = Mk marker; *FLT3* = LP marker; *MPO* = Gran/Mo marker; *MS4A2* = Baso marker. **(c)** Pseudotime values projected onto the force-directed graph. **(d)** Differentiation vector calculated from the pseudotime gradient. ME and GM lineages are highlighted. **(e)** *CSF1R* and *CEBPE* gene expression projected onto the force-directed graph. The right panel is a magnified area of the GM lineage. The *CSF1R* is a monocyte marker, and *CEBPE* is a granulocyte marker. **(f)** Early lineage bifurcation between monocytes and granulocytes is observed on the force-directed graph.

We next evaluated eight additional TFs that have established roles in myeloid differentiation: *Klf1* (also known as *Eklf)*, *Gfi1b*, *Fli1*, *Gfi1*, *Gata2*, *Lmo2*, *Runx1* and *Irf8* (refs. ^15,29^). CellOracle also correctly reproduced their reported KO phenotypes (Extended Data Figs. 5 and 6), which we extended to two additional datasets of mouse and human haematopoiesis (Extended Data Figs. 7 and 8 and Supplementary Figs. 13 and 14). In addition, we scaled up our simulation to all TFs that passed filtering (Methods) to systematically perturb 90 TFs in the dataset in the context of granulocyte–monocyte (GM) and megakaryocyte–erythroid (ME) differentiation. The reported cell-fate-regulatory functions of these TFs fall into three major categories: (1) ME lineage differentiation; (2) GM lineage differentiation; and (3) ME and GM lineage differentiation and maintenance of haematopoietic stem cell (HSC) identity (Supplementary Table 2). We ranked the TFs on the basis of the sum of the negative perturbation score in the KO simulation, representing the potential of a TF potential to promote differentiation (Methods and Extended Data Fig. 3f).

To summarize this systematic TF perturbation, the summed negative perturbation scores are shown on a scatter plot (Fig. 1i). The dashed lines represent cut-off values calculated with a randomized vector (Extended Data Fig. 3g). The distribution of negative perturbation score sums for all TF KOs was highly consistent with known TF functions in differentiation. For example, TFs involved in ME lineage differentiation are enriched on the top left side of the scatter plot. By contrast, GM differentiation factors are found at the bottom right. TFs that regulate both lineages are located on the top right side, whereas the lower-ranked factors are enriched for TFs that have not been reported to regulate blood differentiation (Fig. 1i). Overall, 85% of the top 30 TFs ranked by this objective, systematic perturbation strategy are reported regulators of myeloid differentiation (Supplementary Table 2). Of the remaining TFs, several have no reported phenotypes in haematopoiesis at present, and therefore represent putative regulators. We note that the negative perturbation score metric does not always convey all information of the vector field, which might oversimplify the role of a TF. For example, *Elf1* has a negative perturbation score in both the ME and the GM lineage, and its function is unclear on the summarized perturbation score plot; however, closer inspection of the vector reproduced its reported phenotype in the ME lineage, highlighting the importance of investigating the simulation output (Supplementary Fig. 11). Finally, we directly compared the output of CellOracle to existing methods for identifying regulatory TFs using gene expression and chromatin accessibility, demonstrating the unique insights into context-dependent TF regulation that CellOracle can provide (Supplementary Figs. 15 and 16).

**Fig. 2:**
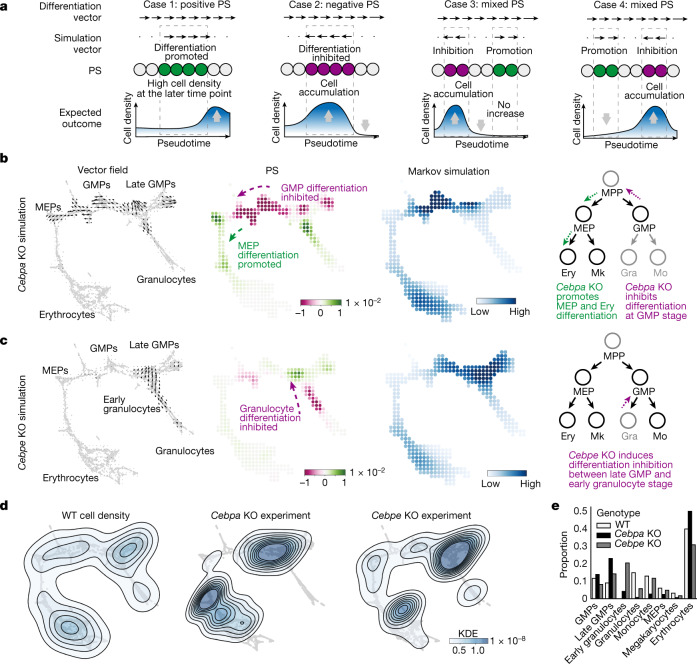
Validation of CellOracle using experimentally measured cell density in *Cebpa* and *Cebpe* KOs in haematopoiesis. **a**, Biological interpretation of perturbation scores (estimation of cell density based on perturbation score). Case 1: the differentiation and perturbation simulation vectors share the same direction, indicating a population shift towards a more differentiated identity. Case 2: the two vectors are opposed, suggesting that differentiation is inhibited. Case 3: predicted inhibition precedes promotion; thus, cells will be likely to accumulate. **b**,**c**, CellOracle *Cebpa* KO (**b**) and *Cebpe* KO (**c**) simulations showing cell-state transition vectors, perturbation scores and estimated cell density (Markov simulation). Right, schematics of simulated phenotype. Ery, erythrocyte. **d**, Ground-truth experimental cell density plot of wild-type (WT) cells, *Cebpa* KO cells and *Cebpe* KO cells in the force-directed graph embedding space. Estimated kernel density data are shown as a contour line on a scatter plot to depict cell density. **e**, Cell-type proportions in the WT and ground-truth KO samples. Gra, granulocyte; KDE, kernel density estimation; Mo, monocyte.

We further validated CellOracle simulation by focusing on several genes for which experimental KO scRNA-seq data are available: *Cebpa*, *Cebpe* and *Tal1* (refs. ^16,30^). *Cebpa* is necessary for the initial differentiation of GMPs, and its loss leads to a marked decrease in differentiated myeloid cells, accompanied by an increase in erythroid progenitors. By contrast, *Cebpe* is not required for initial GMP differentiation, but it is essential for the subsequent maturation of GMPs into granulocytes^16^. Notably, when we compare the simulation results to the experimental KO cell distribution, we must again consider the effects of TF perturbation in the context of natural cell differentiation (Fig. 2a). Thus, we performed a Markov random walk simulation based on the differentiation and simulation vectors to estimate how TF perturbation leads to changes in cell distribution (Supplementary Fig. 17 and Methods). For *Cebpa*, CellOracle simulation predicted that differentiation is inhibited at GMP–late GMP clusters, whereas early erythroid differentiation is promoted (Fig. 2b). The simulation recapitulates the experimental cell distribution (Fig. 2b,d). For *Cebpe*, CellOracle again correctly modelled the inhibition of differentiation at the entry stage of granulocyte differentiation (Fig. 2c), consistent with experimental KO data (Fig. 2d).

**Extended Data Fig. 9:**
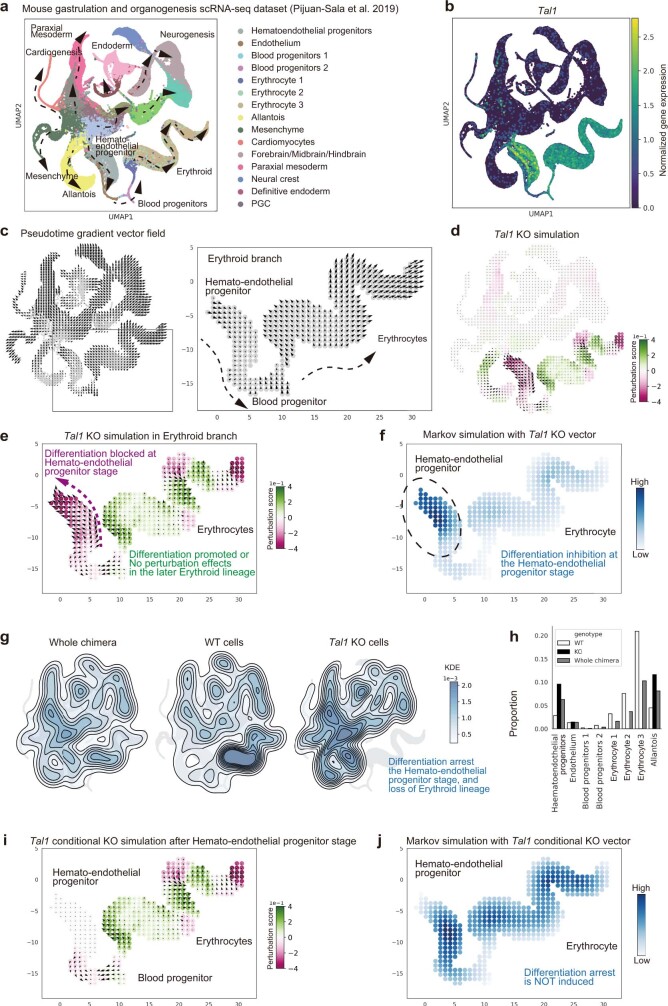
CellOracle validation using experimentally measured cell density in *Tal1* KO in Pijuan-Sala et al. gastrulation and organogenesis scRNA-seq data. **(a)** UMAP plot of chimeric E8.5 embryos of wild-type (WT) and *Tal1* KO cells (25,307 cells and 26,311 cells, respectively) from a published scRNA-seq atlas of mouse gastrulation and organogenesis^30^. **(b)** *Tal1* gene expression (log-transformed UMI) projected onto the UMAP plot. **(c)** Pseudotime gradient vector field used in the perturbation score (PS) calculations. Developmental pseudotime was calculated using the DPT method with WT chimera scRNA-seq data and then converted into a 2D gradient vector field. **(d)** PS and cell transition vector field of the *Tal1* KO simulation. **(e)** The magnified area of erythrocyte differentiation predicts inhibition or arrest of cell differentiation at the haematoendothelial progenitor stage. **(f)** The Markov random walk simulation result predicts high cell density in the haematoendothelial progenitor cluster and lower cell density at later stages, indicating that *Tal1* KO would induce differentiation arrest at the haematoendothelial progenitor stage. **(g)** Experimentally measured *Tal1* KO data. The kernel cell density of whole chimera (left), WT (middle), and *Tal1* KO cells (right) were calculated after downsampling each condition (25,307 cells) to control for sample size. A scatter plot of whole chimera cells is shown as background (light grey) to highlight the overall cell trajectory structure. **(h)** The bar plot shows the cell type composition in each sample (right panel). Overall, the experimental result aligns with the simulated predictions. The relative fold change between WT and KO samples is also shown in Supplementary Table 4. **(i)** Perturbation score and cell transition vector field of the *Tal1* conditional KO simulation in the erythroid lineage. *Tal1* expression was set to zero in the Blood progenitor and Erythrocyte clusters; CellOracle simulates KO effects in later erythroid differentiation stages. **(j)** The Markov simulation result shows uniform cell density, predicting that *Tal1* KO would not induce differentiation arrest in a conditional KO targeting later stages of erythroid differentiation.

We also analysed a single-cell atlas of mouse organogenesis^30^ to simulate the loss of *Tal1* function (Extended Data Fig. 9a–d). CellOracle reproduced the inhibited differentiation of haematoendothelial progenitors in the *Tal1* KO^30^ (Extended Data Fig. 9e–h). In addition, CellOracle showed that loss of *Tal1* in later stages of erythroid differentiation does not block cell differentiation (Extended Data Fig. 9i,j), consistent with previous conditional *Tal1* KO experiments at equivalent stages^31^. Together, these results show that CellOracle effectively simulates cell-state-specific TF function, corroborating previous knowledge of the mechanisms that regulate cell fate in haematopoiesis and ground-truth in vivo phenotypes. Furthermore, systematic KO simulations demonstrate that CellOracle enables objective and scalable in silico gene perturbation analysis.

## Systematic TF KO simulations in zebrafish

**Extended Data Fig. 10:**
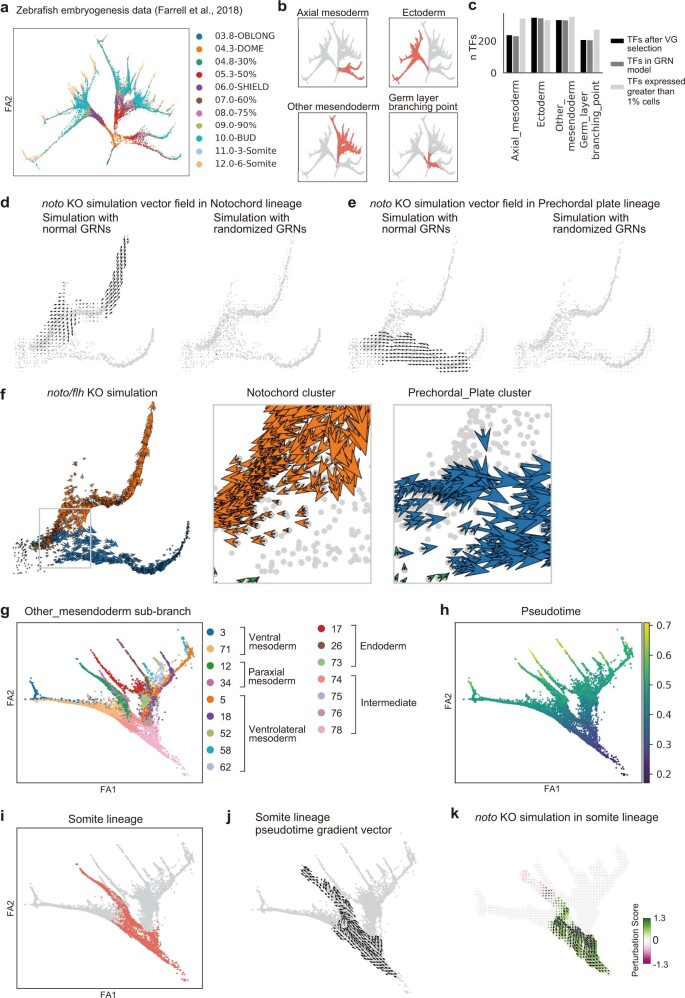
CellOracle *noto* LOF simulation with Farrell et al. zebrafish embryogenesis data. **(a)** 2D force-directed graph of a published atlas^32^ of zebrafish embryogenesis (n = 25,711 cells). **(b)** Main trajectory partitioned into four sub-branches. **(c)** Bar plots depicting the number of TFs after variable gene selection (black), the number of TFs with >1 network edge in the inferred GRN model (dark grey), and the number of TFs expressed in >1% of cells (light grey). **(d)** CellOracle *noto* LOF simulation result (left) and simulation results with a randomized GRN model (right) for the notochord lineage. Simulated cell state transitions for each cell were converted to a vector field and visualized with a scatter plot (shown in grey). **(e)** *Noto* LOF simulation for the prechordal plate lineage. **(f)** CellOracle *noto* LOF simulation vector is shown at single-cell resolution. Cells in the Notochord cluster are shown in orange, while the Prechordal Plate cells are shown in blue. The right panel is the magnified area. **(g)** Force-directed graph of the Other mesendoderm sub-branch with cell cluster annotations from the Farrell et al. study^32^ (n = 10,265 cells). **(h)** Pseudotime data are projected onto the force-directed graph. **(i)** The Somite lineage, defined in the previous Farrell et al. study^32^, is in red. **(j)** Pseudotime gradient vector field calculated for the Somite lineage. **(k)** *Noto* LOF simulation vector field in the cells of the Somite lineage are shown with perturbation scores.

Next, we applied CellOracle to systematically perturb TFs across zebrafish development. We made use of a 38,731-cell atlas of zebrafish embryogenesis published in a study by Farrell et al.^32^, comprising 25 developmental trajectories that span zygotic genome activation to early somitogenesis. We first inferred GRN configurations for the 38 cell types and states identified in the Farrell et al. study^32^, splitting the main branching trajectory into four sub-branches: ectoderm; axial mesoderm; other mesendoderm; and germ layer branching point (Extended Data Fig. 10a,b). In the absence of scATAC-seq data, we constructed a base GRN using promoter information from the UCSC database, obtaining information on TF-binding motifs from the *Danio rerio* CisBP motif database (Methods). Our benchmarking has shown that this approach produces reliable GRN inference (Extended Data Fig. 2). After preprocessing and GRN inference, we performed KO simulations for all TFs with inferred connections to at least one other gene (*n* = 232 ‘active’ TFs; Methods). The results of these simulations across all developmental trajectories can be explored at https://www.celloracle.org.

**Fig. 3:**
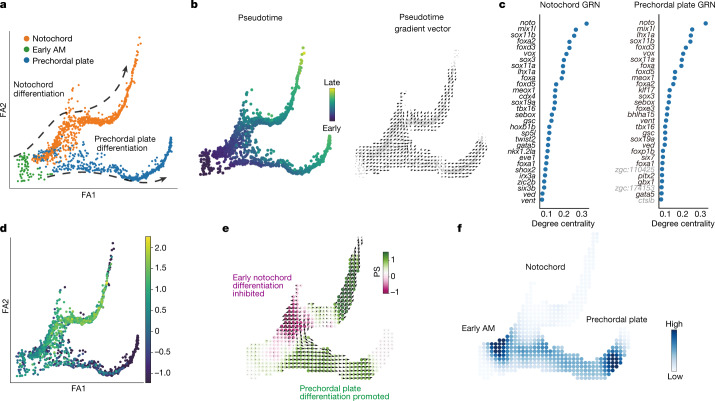
CellOracle KO simulation with zebrafish embryogenesis data. **a**, Two-dimensional force-directed graph of the axial mesoderm (AM) sub-branch (*n* = 1,669 cells) in a published zebrafish embryogenesis atlas (Farrell et al.^32^). Arrows indicate notochord cell differentiation (top) and prechordal plate differentiation (bottom). **b**, Conversion of URD-calculated pseudotime (left) into a 2D pseudotime gradient vector field (right). **c**, Degree centrality scores were used to rank the top 30 TFs in notochord (left) and prechordal plate (right). Black text denotes TFs. Grey text denotes non-TFs. **d**, Expression of *noto* projected onto the axial mesoderm sub-branch. **e**, *Noto* KO simulation vector and perturbation scores. **f**, Markov simulation to estimate cell density in the *noto* KO sample. The simulation predicted inhibited early notochord differentiation and promotion of prechordal plate differentiation, indicating a potential lineage switch.

Our systematic TF KO simulation provides a valuable resource for identifying regulators of early zebrafish development and enables candidates to be prioritized for experimental validation. To further examine this comprehensive perturbation atlas, we focused on axial mesoderm differentiation, spanning 4.3 to 12 h post-fertilization (hpf) (Fig. 3a,b and Extended Data Fig. 10a,b). This midline structure bifurcates into notochord and prechordal plate lineages, representing a crucial patterning axis^33^, and has been extensively characterized, in part through large-scale genetic screens^34^. For these lineages, we performed systematic TF KO simulation and network analysis for 232 candidate TFs (Extended Data Fig. 10c). CellOracle ranked *noto*, a well-characterized TF regulator of notochord development, as the top TF on the basis of degree centrality, along with other known regulators of notochord development (Fig. 3c). Degree centrality is a straightforward measure that reports how many edges (genes) are directly connected to a node (TF); highly connected nodes are likely to be essential for a biological process^35,36^. In zebrafish *floating head*^*n1/n1*^ (*flh*^*n1/n1*^) mutants, which lack a functional *noto* gene (*noto* is also known as *flh*)^37^, axial mesoderm does not differentiate into notochord, and assumes a somitic mesoderm fate instead^38^. *Noto* LOF simulation correctly reproduced the loss of notochord (Fig. 3d–f and Extended Data Fig. 10d–f), in addition to enhanced somite differentiation (Extended Data Fig. 10g–k). Moreover, CellOracle predicted a previously unknown (to our knowledge) consequence of *noto* LOF: enhanced prechordal plate differentiation (Fig. 3e,f). We also noted that later stages of notochord differentiation received a positive perturbation score, indicating that continued expression of *noto* is not required for notochord differentiation. Alternatively, this finding could suggest that downregulation of *noto* is required for notochord maturation.

## Experimental validation of *noto* LOF

**Fig. 4:**
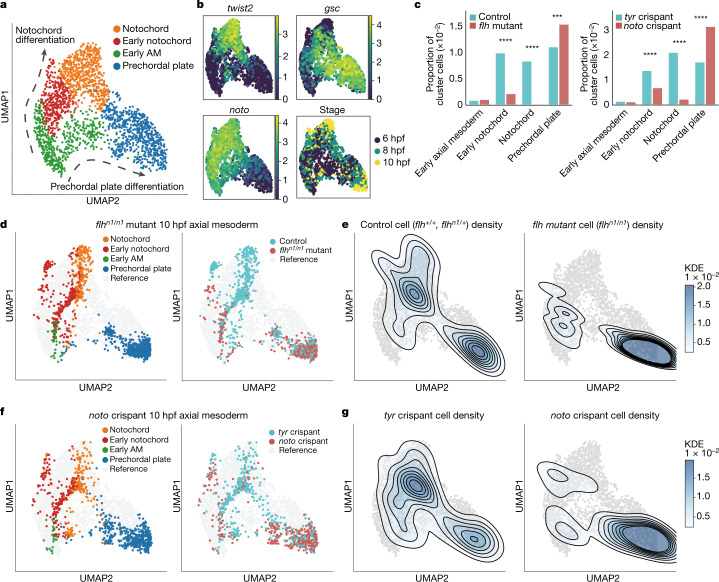
Experimental validation of zebrafish *noto* LOF predictions. **a**, UMAP plot of WT reference data for axial mesoderm (6, 8 and 10 hpf): notochord, early notochord, early axial mesoderm and prechordal plate clusters (*n* = 2,012 cells). Arrows indicate notochord differentiation (top) and prechordal plate differentiation (bottom). **b**, Gene expression (log-transformed unique molecular identifier (UMI) count) and developmental stage are projected onto the axial mesoderm UMAP plot. *Noto* and *twist2* are expressed in notochord, whereas *gsc* marks the prechordal plate. **c**, Bar plots comparing cell cluster compositions between treatments and controls (left, *flh*^*n1/n1*^ mutants (10 hpf) and controls; right, *noto* crispants (10 hpf) and *tyr* crispants). Cluster compositions are presented as the proportion of each group normalized to the whole cell number. In both *flh*^*n1/n1*^ mutants and *noto* crispants, the notochord is significantly depleted (*flh*^*n1/n1*^: *P* = 5.55 × 10^−52^; noto: *P* = 1.39 × 10^−33^, chi-square test) and the prechordal plate is significantly expanded (*flh*^*n1/n1*^: *P* = 1.07 × 10^−4^; *noto*: *P* = 5.01 × 10^−18^, chi-square test. ***P < 0.001; ****P < 0.0001). **d**–**g**, *flh*^*n1/n1*^ mutant or *noto* crispant data projected onto the WT axial mesoderm UMAP plot. **d**, Cluster annotation and sample label projected onto the UMAP plot. **e**, Kernel cell density contour plot shows control cell density (left) and *flh*^*n1/n1*^ mutant cell density (right). **f**, Cluster annotation and sample label projected onto the UMAP plot. **g**, *tyr* crispant cell density (left) and *noto* crispant cell density (right) shown on the kernel cell density contour plot.

**Extended Data Fig. 11:**
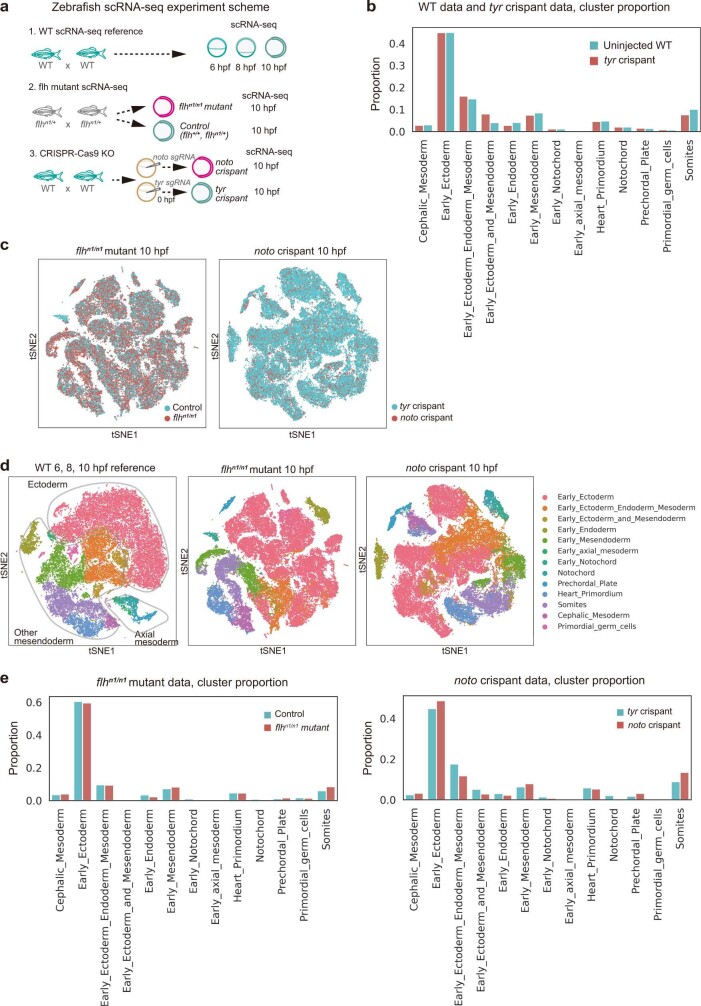
Zebrafish scRNA-seq experiments for *noto* LOF analysis. **(a)** Schematic illustration of zebrafish scRNA-seq experiments. (1) The reference dataset was generated using cells from 6, 8, and 10 hpf wild-type (WT) embryos. To assess *noto* LOF, we also assayed (2) *flh*^*n1/n1*^ mutants and (3) *noto/flh* crispants at 10 hpf (~25 embryos per sample; Methods). **(b)** Cell cluster composition comparing *tyr* crispant (control) with WT cells, showing similar cell distributions. After data integration, cell-type labels were transferred from the whole WT 6, 8, and 10 hpf reference data (see Methods). **(c)** Sample label projected onto the *t-*SNE plot. *flh*^*n1/n1*^ mutant and control sample (left, n = 57,175 cells, 2 independent biological replicates for each sample), and *t-*SNE plot of *noto* crispant and *tyr* crispant samples (right, n = 9,185 cells, 2 biological, 3 technical replicates for *noto* crispant; n = 46,440 cells, n = 3 independent biological, 5 technical replicates for *tyr* crispant). **(d)** Cluster annotation label projected onto the *t-*SNE plot. WT zebrafish cells (left, n = 38,606 cells, two technical replicates per stage), *flh*^*n1/n1*^ mutant and control sample (middle), *noto* crispant and *tyr* crispant samples (right). **(e)** Cell cluster composition comparing LOF samples with the control samples.

Next, we experimentally validated the predicted expansion of prechordal plate after *noto* LOF. First, we generated a 38,606-cell wild-type (WT) reference atlas from dissociated WT embryos at 6, 8 and 10 hpf (2 technical replicates per stage) and used Seurat’s label transfer function^39^ to cluster and label the WT reference cells according to the annotations in Farrell et al.^32^ (Extended Data Fig. 11). Subsetting the axial mesoderm clusters showed the expected bifurcation of cells into notochord and prechordal plate, accompanied by upregulation of marker genes (Fig. 4a,b). For visualization of axial mesoderm cells, we used a uniform manifold approximation and projection (UMAP) transfer function to enable comparable data visualization between different samples (Methods).

For experimental perturbation of *noto*, we generated and dissociated pools of 25 *flh*^*n1/n1*^ mutant embryos, recognized at 10 hpf by the lack of notochord boundaries, and sibling controls (*flh*^*n1/+*^ and *flh*^*+/+*^) for scRNA-seq. We integrated these datasets and projected them onto the WT axial mesoderm reference atlas. In agreement with previous observations, we observed a significant depletion of cells labelled as notochord in *flh*^*n1/n1*^ mutants (−98%, relative to control, *P* = 5.55 × 10^−52^, chi-square test; Fig. 4c, left), concomitant with an expansion of the somite cluster (+41.3%; *P* = 5.90 × 10^−29^; Extended Data Fig. 11e, left). Furthermore, as predicted by *noto* LOF simulation, we observed a significant expansion of the prechordal plate cluster in *flh*^*n1/n1*^ mutants (+38.6%; *P* = 1.07 × 10^−4^; Fig. 4c, left). Plotting cell density revealed stalled notochord differentiation and bifiurcation of the mid axial mesoderm, with excess prechordal plate cells (Fig. 4d,e), consistent with the *noto* LOF simulation (Fig. 3e,f). To orthogonally validate these results, we produced *noto* LOF with a modified CRISPR–Cas9 protocol that we have previously used to achieve near-complete gene disruption in F_0_ embryos injected with two *noto*-targeting guide RNAs (gRNAs)^40^ (Methods). The resulting *noto* ‘crispants’ were dissociated at 10 hpf (9,185 cells, *n* = 2 biological and *n* = 3 technical replicates) and compared by single-cell analysis to controls that targeted the *tyrosinase* gene (*tyr)*, which is not expressed until later in development (*n* = 46,440 single cells, *n* = 3 biological and *n* = 5 technical replicates; Extended Data Fig. 11b). Analysis of cell-type composition confirmed a significant depletion of notochord, with an expansion of somitic mesoderm and prechordal plate (Fig. 4c, right, Fig. 4f,g and Extended Data Fig. 11e, right) in *noto* crispants, highly consistent with our *flh*^*n1/n1*^ mutant analysis. Together, in addition to further validating the performance of CellOracle, these results highlight the ability of this approach to identify experimentally quantifiable phenotypes in well-characterized mutants that may have been previously overlooked owing to a reliance on gross morphology. We next sought to identify new LOF phenotypes in axial mesoderm development.

## Discovery of axial mesoderm regulators

**Fig. 5:**
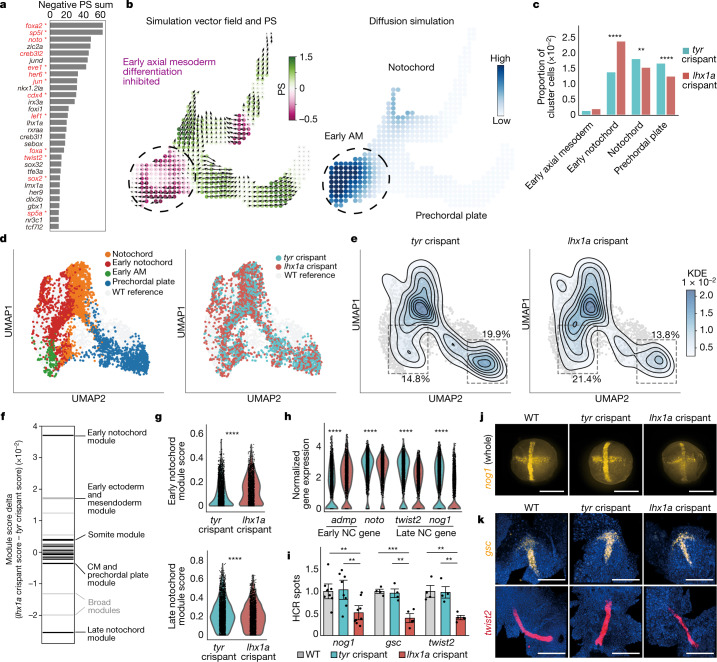
Experimental validation of *lhx1a* as a putative regulator of zebrafish axial mesoderm development. **a**, Top 30 TFs according to predicted KO effects. Red and *: previously reported notochord regulators (Supplementary Table 2). *lhx1a*, *sebox* and *irx3a* were selected for experimental validation. **b**, *lhx1a* LOF simulation in the axial mesoderm sub-branch, predicting an inhibition of axial mesoderm differentiation from early stages. **c**, scRNA-seq validation of experimental LOF: cell cluster composition of the axial mesoderm clusters normalized to the whole cell number in *lhx1a* and *tyr* (control) crispant samples. Early notochord is significantly expanded (*P* = 1.34 x 10^−35^, chi-square test) and differentiated axial mesoderm populations are significantly depleted (notochord: *P* = 3.83 x 10^−3^; prechordal plate: *P* = 1.28 x 10^−7^, chi-square test) in *lhx1a* crispants. **d**, *lhx1a* and *tyr* crispant axial mesoderm cells at 10 hpf. Left, cell type annotation of *lhx1* and *tyr* crispant cells. Right, *lhx1a* and control crispant data projected onto the WT UMAP. **e**, Control cell density (left, *n* = 2,342 cells) and *lhx1a* crispant cell density (right, *n* = 2,502 cells). **f**, Rug plot showing the difference in averaged NMF module scores between *lhx1a* and *tyr* crispants in notochord lineage cells. Black, cell-type-specific modules. Light grey, broad cluster modules. CM, cephalic mesoderm. **g**, Violin plot of NMF module score in notochord lineage cells (*n* = 1,918 *lhx1a* crispant and *n* = 2,616 *tyr* crispant cells. **h**, Violin plots of gene expression in the notochord (NC) lineage cells. ****P < 0.0001, two-tailed Wilcoxon rank-sum test with Bonferroni correction. **i**, Quantification (number of spots in flattened HCR image) normalized to WT. Mean ± s.e.m. *n* = 2 independent biological replicates, 8 embryos per replicate. *nog1*: *P* = 0.0022 (WT versus *lhx1a* crispant), *P* = 0.0052 (*tyr* versus *lhx1a* crispant); *gsc*: *P* = 0.00042 (WT versus *lhx1a* crispant), *P* = 0.0018 (*tyr* versus *lhx1a* crispant); *twist2*: *P* = 0.0011 (WT versus *lhx1a* crispant), *P* = 0.0012 (*tyr* versus *lhx1a* crispant); two-sided *t*-test. **j**, Representative HCR images for *nog1* expression (yellow) in whole embryos at 10 hpf. **k**, Representative flattened HCR images of 10 hpf embryos stained with probes against *gsc* (yellow) and *twist2* (red); nuclei are stained with DAPI (blue). Scale bars, 300 μm.

**Extended Data Fig. 12:**
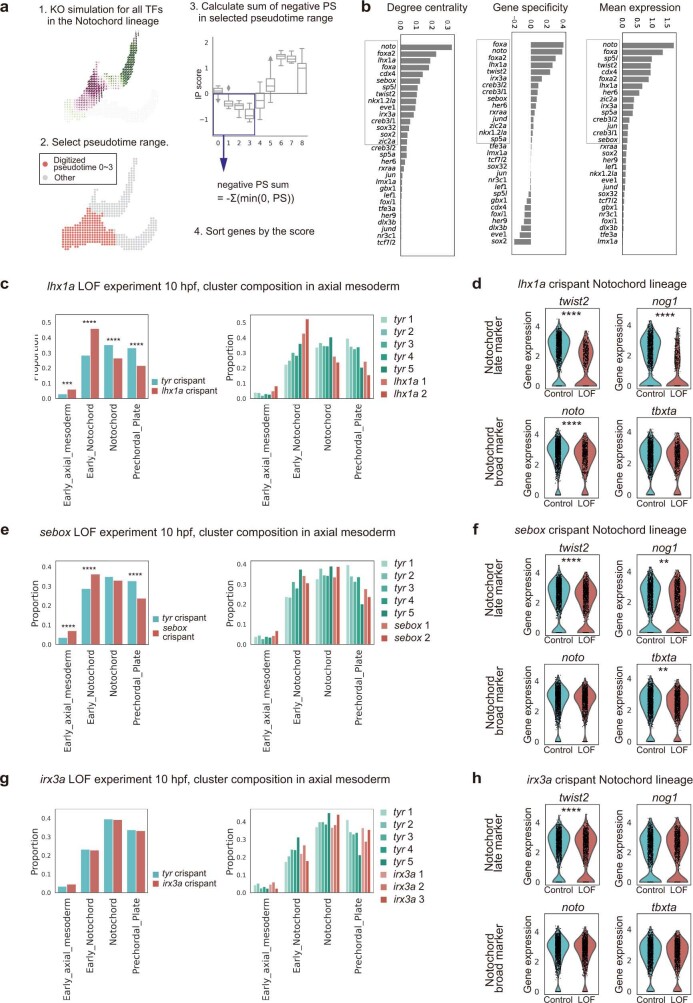
Zebrafish notochord regulator screening with CellOracle and initial experimental validation. **(a)** Overview of the systematic LOF simulation and quantification method. CellOracle LOF simulation was performed for 232 TFs in the Notochord lineage to calculate the perturbation score (PS). The sum of the negative PS was calculated for each TF in the selected area between digitized pseudotime 0 to 3, before lineage specification. **(b)** Degree centrality score in the Notochord cluster GRN (left), gene expression specificity score in the Axial mesoderm sub-branch (middle), and mean expression value in the Axial mesoderm sub-branch (right) were calculated for the top 30 TFs selected in the systematic simulation to further prioritize candidate genes for experimental validation. We selected genes in the top 50% of these scores. Please refer to the Methods for the detailed selection procedure. We selected three candidates for experimental validation: *lhx1a*, *sebox*, and *irx3a*. (**c**,**e**,**g**) Cell cluster composition in axial mesoderm cells, comparing LOF (*lhx1a*, *sebox*, and *irx3a)* samples with control samples. Cell cluster composition comparison was performed with a Chi-square test, Two-tailed Bonferroni correction. *lhx1a* experiment: Early axial mesoderm p = 0.000229717, Early Notochord p = 1.08×10^−21^, Notochord p = 4.38×10^−6^, Prechordal Plate p = 1.42×10^−10^. *Sebox* experiment: Early axial mesoderm p = 3.01×10^−6^, Early Notochord p = 2.87×10^−6^, Notochord p = 4.38×10^−6^, Prechordal Plate p = 4.17×10^−9^. The left panels show cluster composition in the merged data, and the right panels show individual scRNA-seq batch. *lhx1a* LOF produced the most significant changes in cell composition. (**d**,**f**,**h**) Comparison of notochord marker gene expression between LOF and control samples. scRNA-seq gene expression in the Notochord lineage clusters is shown as a violin plot. Late-stage notochord markers, *twist2* and *nog1*, or broad/early notochord markers, *noto* and *tbxta*, are visualized. Statistical tests: Wilcoxon rank-sum test, two-tailed with Bonferroni p-value correction. *lhx1a* experiment: *twist2* p = 7.118×10^−64^, *nog1* p = 7.757×10^−67^, *noto* p = 7.718×10^−11^. *sebox* experiment: *twist2* p = 8.022×10^−10^, *nog1* p = 3.184×10^−3^, *tbxta* p = 1.551×10^−3^. *irx3a* experiment: *twist2* p = 0.000012. **(c)** n = 720 cells and 1,686 cells for *lhx1a* crispant and *tyr* crispant, respectively. **(e)** n = 1,216 cells and 1,703 cells for *sebox* crispant and *tyr* crispant, respectively. **(g)** n = 1,176 cells and 1,651 cells for *irx3a* crispant and *tyr* crispant, respectively.

To identify novel TFs required for axial mesoderm differentiation, we prioritized TFs according to predicted KO phenotypes, focusing on early-stage differentiation before evident lineage specification (Extended Data Fig. 12a). The resulting ranked list contains several known notochord regulators, including *noto* (Fig. 5a, red and Supplementary Table 2), confirming CellOracle’s capacity to model known developmental regulation. However, it is important to note that some known notochord regulators, such as *foxa3* (ref. ^41^), were not identified as they are filtered out in the first steps of data processing owing to low expression. Systematic perturbation simulations for all lineages can be found at https://celloracle.org. As well as the axial mesoderm, we also performed an in-depth analysis of the adaxial mesoderm, which gives rise to somites. Overall, more than 80% of the top 30 TFs in this analysis were associated with somite differentiation (Supplementary Table 3).

In addition to known TFs, we identified several TFs with no previously reported role in axial mesoderm differentiation (Fig. 5a, black). We further prioritized candidate genes for experimental validation by GRN degree centrality, gene enrichment score in axial mesoderm and average gene expression value, selecting *lhx1a*, *sebox* and *irx3a* (Extended Data Fig. 12b). CellOracle predicts impaired notochord differentiation for all three genes after their LOF (Fig. 5b and Supplementary Fig. 19). However, no LOF studies describing axial mesoderm phenotypes that relate to these genes have, to our knowledge, been reported in zebrafish. Mouse *Lhx1 (Lim1)* KO embryos lack anterior head structures and kidneys^42^. In zebrafish, *sebox* (*mezzo*) has been implicated in mesoderm and endoderm specification^43^, whereas *irx3a* (*ziro3*) morphants exhibit changes in the composition of pancreatic cell types^44^.

We generated *lhx1a*, *sebox* and *irx3a* crispants (Supplementary Fig. 20b–d). We performed initial single-cell analyses at 10 hpf, integrating crispant scRNA-seq datasets with the control gRNA reference atlas described above. We observed significant changes in cell-type composition and notochord marker expression in *lhx1a* crispants (Extended Data Fig. 12c,d and Supplementary Table 4). Notably, we found a more considerable reduction in the expression of late notochord genes relative to broad notochord markers, suggesting that loss of *lhx1a* function inhibits the differentiation and maturation of notochord cells. We observed a slight yet significant reduction in the expression of the notochord markers *twist2*, *nog1* and *tbxta* in *sebox* crispants (Extended Data Fig. 12e,f and Supplementary Table 4), confirming CellOracle’s predictions that *lhx1a* and *sebox* are regulators of axial mesoderm development. *Irx3a* crispants showed no significant phenotype in cell-type composition but exhibited a slight reduction in *twist2* expression in the notochord (Extended Data Fig. 12g,h).

**Extended Data Fig. 13:**
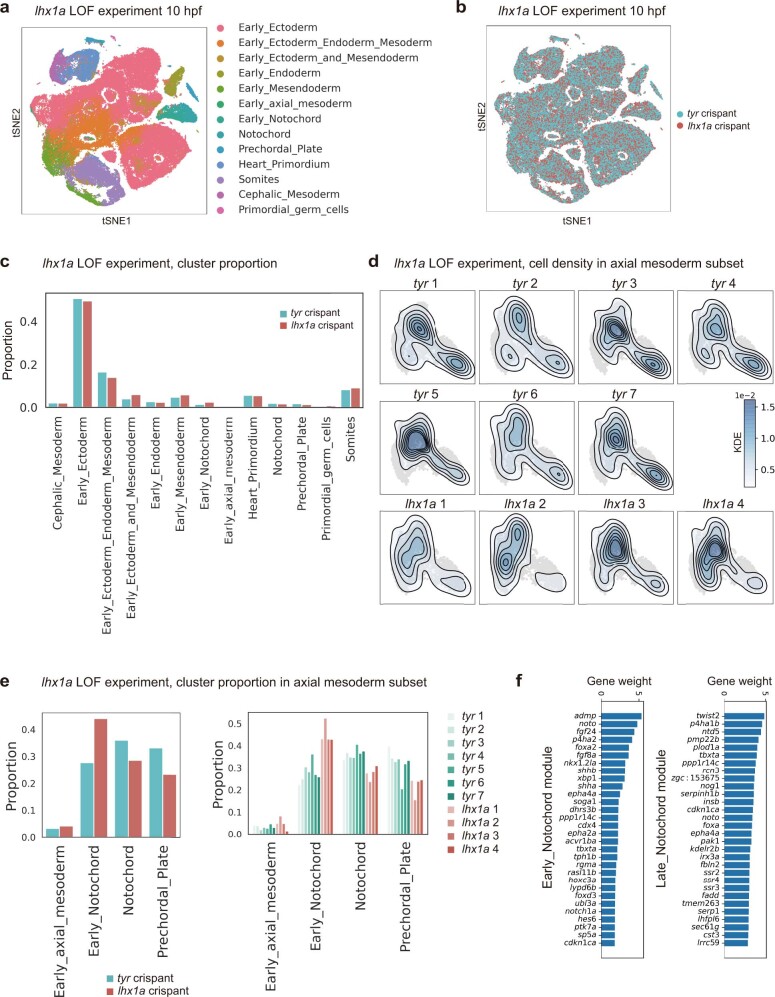
Zebrafish scRNA-seq experiments for *lhx1a* LOF analysis. (**a**,**b**) *t-*SNE plot of *lhx1a* crispant (n = 45,582 cells, 4 biological replicates) and *tyr* control crispant samples (n = 76,163 cells, 5 biological, 7 technical replicates). **(a)** Cluster annotation labels transferred from WT reference data projected onto the *t-*SNE plot. **(b)** Sample label projected onto the *t-*SNE plot. **(c)** Cell cluster composition comparing *lhx1a* crispant and *tyr* control crispant samples as a proportion of cells from the whole embryo. **(d)** Cell density in the axial mesoderm is visualized as a kernel cell density contour plot. The cell number is downsampled to match the cell number before kernel cell density calculation (n = 260, 290, 336, and 367 for *lhx1a* crispant 1~4, n = 248, 234, 344, 316, 213, 286, and 350 for *tyr* crispant 1~7). The same contour threshold values are used for the visualization. **(e)** Cell cluster composition in the axial mesoderm clusters comparing *lhx1a* crispant and *tyr* control crispant samples. The left panels show cluster composition in the merged data, while the right panels show the individual scRNA-seq batch. **(f)** The top 30 NMF module weights for the Early notochord module (left) and the Late notochord module (right) are shown as a bar plot.

We extended *lhx1a* LOF characterization by performing four independent biological replicates for *lhx1a* crispants (*n* = 45,582 cells) and *tyr* crispants (*n* = 76,163 cells, 5 biological and 7 technical replicates). CellOracle predicted inhibition of early axial mesoderm differentiation after *lhx1a* disruption, depleting both notochord and prechordal plate lineages (Fig. 5b). Indeed, the *lhx1a* crispants exhibited inhibition of axial mesoderm differentiation (Fig. 5c–e): a significant expansion of the early notochord cluster (+70.2%; *P* = 1.34 × 10^−35^), with a concomitant reduction of later notochord (−15.3%; *P* = 3.83 × 10^−3^) and prechordal plate clusters (−24.7%; *P* = 1.28 × 10^−7^). These phenotypes were reproducible across independent biological replicates (Extended Data Fig. 13e), validating the predicted inhibition of early axial mesoderm differentiation (Fig. 5a,b).

To further analyse the *lhx1a* LOF axial mesoderm phenotype, we investigated global changes in gene expression across all cell types using non-negative matrix factorization (NMF), a method to quantify gene module activation^45^ (Supplementary Table 5 and Methods). We observed that a module corresponding to the early notochord was significantly activated in *lhx1a* crispants (*P* = 2.62 × 10^−32^; Fig. 5f,g). The top gene in this module is *admp* (Extended Data Fig. 13f, left), which is significantly upregulated in *lhx1a* crispant cells (*P* = 6.69 × 10^−46^; Fig. 5h) and encodes a known negative regulator of notochord and prechordal plate development^46^. By contrast, the late notochord module received a significantly lower score in the *lhx1a* crispant cells (*P* = 1.04 × 10^−5^; Fig. 5g, bottom). This module comprises late notochord marker genes, such as *twist2* and *nog1* (Extended Data Fig. 13f, right), which showed significantly lower expression in *lhx1a* crispant cells (*P* = 4.52 × 10^−105^ and *P* = 4.95 × 10^−105^, respectively; Fig. 5h). Further, *lhx1a* crispant cells exhibited a higher somite module score (*P* = 5.19 × 10^−25^ and Supplementary Table 5), suggesting that notochord cells may be redirected towards a somitic identity after *lhx1a* LOF. Overall, the NMF analysis supports the hypothesis that loss of *lhx1a* function induces global changes in gene expression that are related to inhibited notochord differentiation.

Finally, we confirmed the *lhx1a* LOF phenotype using orthogonal approaches. Hybridization chain reaction (HCR) RNA fluorescence in situ hybridization for *nog1* (late notochord) and for *gsc* and *twist2* (prechordal plate and notochord, respectively) showed that these genes were significantly downregulated in *lhx1a* crispants (Fig. 5i–k). These results were further confirmed by quantitative reverse transcription PCR (qRT-PCR) and whole-mount in situ hybridization against *nog1* (Supplementary Fig. 22). Together, this experimental validation confirms the significant and consistent disruption of axial mesoderm development after loss of *lhx1a* function. In summary, these results demonstrate the ability of CellOracle to accurately predict known TF perturbation phenotypes, provide insight into previously characterized mutants and reveal regulators of established developmental processes in well-studied model organisms.

## Discussion

The emerging discipline of perturbational single-cell omics enables regulators of cell identity and behaviour to be modelled and predicted^5^. For example, scGen combines variational autoencoders with latent space vector arithmetic to predict cell infection response. However, this approach requires experimentally perturbed training data, which limits its scalability^47^. More importantly, it remains challenging to interpret the gene program behind the simulated outcome using these previous computational perturbation approaches because they rely on complex black-box models; thus, the simulations lack any means to interpret how gene regulation relates to cellular phenotype. On the other hand, previous GRN analyses relied largely on static graph theory and could not consider cell identity as a dynamic property. Here we present a strategy that overcomes these limitations by integrating computational perturbation with GRN modelling. CellOracle uses GRN models to yield mechanistic insights into the regulation of cell identity; simulation and vector visualization based on the custom network model enables the interpretable, scalable and broadly applicable analysis of dynamic TF function.

We validated CellOracle using various in vivo differentiation models, verifying its efficacy and its robustness to complex and noisy biological data. CellOracle simulates shifts in cell identity by considering systematic gene-to-gene relationships for each cell state using multimodal data, generating a complex context-dependent vector representation that is not possible using differential gene expression or chromatin accessibility alone. For example, the role of *Gata1* in granulocyte differentiation would probably not be predicted given its low expression in this cell type. However, CellOracle could corroborate this relatively mild *Gata1* phenotype. Furthermore, CellOracle correctly reproduced the reported early-stage-specific cell-fate-regulatory role of *Tal1* in erythropoiesis, which is impossible to uncover on the basis of the constitutive expression of *Tal1* throughout all erythroid stages. This capacity of CellOracle means that it could identify previously unreported phenotypes. For example, the LOF simulation of a well-characterized regulator of zebrafish axial mesoderm development, *noto*, predicted a previously unreported expansion of the prechordal plate, which we experimentally validated. This case suggests that *noto* has a role in suppressing alternate fates, which could only be predicted by the integrative simulation using the GRN and cell differentiation trajectory together. Finally, although we focus on TF KO and LOF in this study, we have also recently demonstrated that CellOracle can be used to simulate TF overexpression^48^.

We note some limitations of the method. First, CellOracle visualizes the simulation vector within the existing trajectory space; thus, cell states that do not exist in the input scRNA-seq data cannot be analysed. Nevertheless, existing single-cell data collected after severe developmental disruption do not report the emergence of new transcriptional states in the context of loss of gene function, which suggests extensive canalization even during abnormal development^32^, supporting the use of CellOracle to accurately simulate TF perturbation effects. Second, we emphasize that TF simulation is limited by input data availability and data quality. For example, a perturbation cannot be simulated if a TF-binding motif is unknown or TF expression is too sparse, as we note in the case of *foxa3* in zebrafish^41^.

Our application of CellOracle to systematically simulate TF perturbation has revealed regulators of a well-characterized developmental paradigm: the formation of axial mesoderm in zebrafish. Although zebrafish axial mesoderm has been well-characterized through mutagenesis screens, a role for Lhx1a in these developmental stages is likely to have gone unreported owing to the absence of gross morphological phenotypical changes at 10 hpf after disruption of *lhx1a* (ref. ^49^). However, our ability to predict and validate such a phenotype showcases the power of single-cell computational and experimental approaches, enabling finer-resolution dissection of gene regulation even in well-characterized systems. Moreover, CellOracle provides information at intermediate steps in a given developmental pathway, obviating the need for gross morphological end-points. Indeed, each simulation can be thought of as many successive predictions along a lineage, although we stress that experimental validation is essential to validate CellOracle’s predictions where possible. However, applying these approaches to emerging systems or where experimental intervention is not feasible promises to accelerate our understanding of how cell identity is regulated. For example, in the context of human development, we have recently applied CellOracle to predict candidate regulators of medium spiny neuron maturation in human fetal striatum^50^, demonstrating the power of in silico perturbation where experimental approaches cannot be deployed.

## Methods

### CellOracle algorithm overview

The CellOracle workflow consists of several steps: (1) base GRN construction using scATAC-seq data or promoter databases; (2) scRNA-seq data preprocessing; (3) context-dependent GRN inference using scRNA-seq data; (4) network analysis; (5) simulation of cell identity following TF perturbation; and (6) calculation of the pseudotime gradient vector field and the inner-product score to generate perturbation scores. We implemented and tested CellOracle in Python (versions 3.6 and 3.8) and designed it for use in the Jupyter notebook environment. CellOracle code is open source and available on GitHub (https://github.com/morris-lab/CellOracle), along with detailed descriptions of functions and tutorials.

### Base GRN construction using scATAC-seq data

In the first step, CellOracle constructs a base GRN that contains unweighted, directional edges between a TF and its target gene. CellOracle uses the regulatory region’s genomic DNA sequence and TF-binding motifs for this task. CellOracle identifies regulatory candidate genes by scanning for TF-binding motifs within the regulatory DNA sequences (promoter and enhancers) of open chromatin sites. This process is beneficial as it narrows the scope of possible regulatory candidate genes in advance of model fitting and helps to define the directionality of regulatory edges in the GRN. However, the base network generated in this step may still contain pseudo- or inactive connections; TF regulatory mechanisms are not only determined by the accessibility of binding motifs but may also be influenced by many context-dependent factors. Thus, scRNA-seq data are used to refine this base network during the model fitting process in the next step of base GRN assembly.

Base GRN assembly can be divided into two steps: (i) identification of promoter and enhancer regions using scATAC-seq data; and (ii) motif scanning of promoter and enhancer DNA sequences.

#### Identification of promoter and enhancer regions using scATAC-seq data

CellOracle uses genomic DNA sequence information to define candidate regulatory interactions. To achieve this, the genomic regions of promoters and enhancers first need to be designated, which we infer from ATAC-seq data. We designed CellOracle for use with scATAC-seq data to identify accessible promoters and enhancers (Extended Data Fig. 1a, left panel). Thus, scATAC-seq data for a specific tissue or cell type yield a base GRN representing a sample-specific TF-binding network. In the absence of a sample-specific scATAC-seq dataset, we recommend using scATAC-seq data from closely related tissue or cell types to support the identification of promoter and enhancer regions. Using broader scATAC-seq datasets produces a base GRN corresponding to a general TF-binding network rather than a sample-specific base GRN. Nevertheless, this base GRN network will still be tailored to a specific sample using scRNA-seq data during the model fitting process. The final product will consist of context-dependent (cell-type or state-specific) GRN configurations.

To identify promoter and enhancer DNA regions within the scATAC-seq data, CellOracle first identifies proximal regulatory DNA elements by locating TSSs within the accessible ATAC-seq peaks. This annotation is performed using HOMER (http://homer.ucsd.edu/homer/). Next, the distal regulatory DNA elements are obtained using Cicero, a computational tool that identifies *cis*-regulatory DNA interactions on the basis of co-accessibility, as derived from ATAC-seq peak information^12^. Using the default parameters of Cicero, we identify pairs of peaks within 500 kb of each other and calculate a co-accessibility score. Using these scores as input, CellOracle then identifies distal *cis*-regulatory elements defined as pairs of peaks with a high co-accessibility score (≥0.8), with the peaks overlapping a TSS. The output is a bed file in which all *cis*-regulatory peaks are paired with the target gene name. This bed file is used in the next step. CellOracle can also use other input data types to define *cis*-regulatory elements. For example, a database of promoter and enhancer DNA sequences or bulk ATAC-seq data can serve as an alternative if available as a .bed file.

For the analysis of mouse haematopoiesis that we present here, we assembled the base GRN using a published mouse scATAC-seq atlas consisting of around 100,000 cells across 13 tissues, representing around 400,000 differentially accessible elements and 85 different chromatin patterns^13^. This base GRN is built into the CellOracle library to support GRN inference without sample-specific scATAC-seq datasets. In addition, we have generated general promoter base GRNs for several key organisms commonly used to study development, including 10 species and 23 reference genomes (Supplementary Table 1).

#### Motif scan of promoter and enhancer DNA sequences

This step scans the DNA sequences of promoter and enhancer elements to identify TF-binding motifs. CellOracle internally uses gimmemotifs (https://gimmemotifs.readthedocs.io/en/master/), a Python package for TF motif analysis. For each DNA sequence in the bed file obtained in step (i) above, motif scanning is performed to search for TF-binding motifs in the input motif database.

For mouse and human data, we use gimmemotifs motif v.5 data. CellOracle also provides a motif dataset for ten species generated from the CisBP v.2 database (http://cisbp.ccbr.utoronto.ca).

CellOracle exports a binary data table representing a potential connection between a TF and its target gene across all TFs and target genes. CellOracle also reports the TF-binding DNA region. CellOracle provides pre-built base GRNs for ten species (Supplementary Table 1), which can be used if scATAC-seq data are unavailable.

### scRNA-seq data preprocessing

CellOracle requires standard scRNA-seq preprocessing in advance of GRN construction and simulation. The scRNA-seq data need to be prepared in the AnnData format (https://anndata.readthedocs.io/en/latest/). For data preprocessing, we recommend using Scanpy (https://scanpy.readthedocs.io/en/stable/) or Seurat (https://satijalab.org/seurat/). Seurat data must be converted into the AnnData format using the CellOracle function, seuratToAnndata, preserving its contents. In the default CellOracle scRNA-seq preprocessing step, zero-count genes are first filtered out by UMI count using scanpy.pp.filter_genes(min_counts=1). After normalization by total UMI count per cell using sc.pp.normalize_per_cell(key_n_counts=‘n_counts_all’), highly variable genes are detected by scanpy.pp.filter_genes_dispersion(n_top_genes=2000~3000). The detected variable gene set is used for downstream analysis. Gene expression values are log-transformed, scaled and subjected to dimensional reduction and clustering. The non-log-transformed gene expression matrix (GEM) is also retained, as it is required for downstream GRN calculation and simulation.

### Context-dependent GRN inference using scRNA-seq data

In this step of CellOracle GRN inference, a machine-learning model is built to predict target gene expression from the expression levels of the regulatory genes identified in the previous base GRN refinement step. By fitting models to sample gene expression data, CellOracle extracts quantitative gene–gene connection information. For signal propagation, the CellOracle GRN model must meet two requirements: (1) the GRN model needs to represent transcriptional connections as a directed network edge; and (2) the GRN edges need to be a linear regression model. Because of this second constraint, we cannot use pre-existing GRN inference algorithms, such as GENIE3 and GRNboost (refs. ^7,51^). CellOracle leverages genomic sequences and information on TF-binding motifs to infer the base GRN structure and directionality, and it does not need to infer the causality or directionality of the GRN from gene expression data. This allows CellOracle to adopt a relatively simple machine-learning model for GRN inference—a regularized linear machine-learning model. CellOracle builds a model that predicts the expression of a target gene on the basis of the expression of regulatory candidate genes:

$${x}_{j}=\,\mathop{\sum }\limits_{i=0}^{n}{b}_{i,j}{x}_{i}+\,{c}_{j},$$

where *x*_*j*_ is single target gene expression and *x*_*i*_ is the gene expression value of the regulatory candidate gene that regulates gene *x*_*j*_. *b*_*i,j*_ is the coefficient value of the linear model (but *b*_*i,j*_ = 0 if *i* = *j*), and *c* is the intercept for this model. Here, we use the list of potential regulatory genes for each target gene generated in the previous base GRN construction step (ii).

$${x}_{i}\in \{{x}_{0},\,{x}_{1},\,\ldots {x}_{n}\}={\rm{Regulatory}}\,{\rm{candidate\; TFs\; of\; gene}}\,{x}_{j}$$

The regression calculation is performed for each cell cluster in parallel after the GEM of scRNA-seq data is divided into several clusters. The cluster-wise regression model can capture non-linear or mixed regulatory relationships. In addition, L2 weight regularization is applied by the Ridge model. Regularization not only helps distinguish active regulatory connections from random, inactive, or false connections in the base GRN but also reduces overfitting in smaller samples.

The Bayesian Ridge or Bagging Ridge model provides the coefficient value as a distribution, and we can analyse the reproducibility of the inferred gene–gene connection (Extended Data Fig. 1a, right). In both models, the output is a posterior distribution of coefficient value *b*:

$${x}_{j}\sim {\rm{N}}{\rm{o}}{\rm{r}}{\rm{m}}{\rm{a}}{\rm{l}}\,\,(\mathop{\sum }\limits_{i=1}^{n}{b}_{i,j}{x}_{i}+{c}_{j},{\epsilon })$$

$$b\sim {\rm{N}}{\rm{o}}{\rm{r}}{\rm{m}}{\rm{a}}{\rm{l}}\,(\,{\mu }_{b},{{\sigma }}_{b})$$

where ${\mu }_{b}$ is the centre of the distribution of *b*, and ${\sigma }_{b}$ is the standard deviation of *b*. The user can choose the model method depending on the availability of computational resources and the aim of the analysis; CellOracle’s Bayesian Ridge requires fewer computational resources, whereas the Bagging Ridge tends to produce better inference results than Bayesian Ridge. Using the posterior distribution, we can calculate *P* values of coefficient *b*; one-sample *t*-tests are applied to *b* to estimate the probability (the centre of *b* = 0). The *P* value helps to identify robust connections while minimizing connections derived from random noise. In addition, we apply regularization to coefficient *b* for two purposes: (i) to prevent coefficient *b* from becoming extremely large owing to overfitting; and (ii) to identify informative variables through regularization. In CellOracle, the Bayesian Ridge model uses regularizing prior distribution of *b* as follows:

$$b\sim {\rm{N}}{\rm{o}}{\rm{r}}{\rm{m}}{\rm{a}}{\rm{l}}\,(0,{{\sigma }}_{b})$$

$${{\sigma }}_{b}^{-1}\sim {\rm{G}}{\rm{a}}{\rm{m}}{\rm{m}}{\rm{a}}\,({10}^{-6},{10}^{-6})$$

${\sigma }_{b}$ is selected to represent non-informative prior distributions. This model uses data in the fitting process to estimate the optimal regularization strength. In the Bagging Ridge model, custom regularization strength can be manually set.

For the computational implementation of the above machine-learning models, we use a Python library, scikit-learn (https://scikit-learn.org/stable/). For Bagging Ridge regression, we use the Ridge class in the sklearn.linear_model and BaggingRegressor in the sklearn.ensemble module. The number of iterative calculations in the bagging model can be adjusted depending on the computational resources and available time. For Bayesian Ridge regression, we use the BayesianRidge class in sklearn.linear_module with the default parameters.

### Simulation of cell identity following perturbation of regulatory genes

The central purpose of CellOracle is to understand how a GRN governs cell identity. Toward this goal, we designed CellOracle to make use of inferred GRN configurations to simulate how cell identity changes following perturbation of regulatory genes. The simulated gene expression values are converted into 2D vectors representing the direction of cell-state transition, adapting the visualization method previously used by RNA velocity^52^. This process consists of four steps: (i) data preprocessing; (ii) signal propagation within the GRN; (iii) estimation of transition probabilities; and (iv) analysis of simulated transition in cell identity.

(i) Data preprocessing
  For simulation of cell identity, we developed our code by modifying Velocyto.py, a Python package for RNA-velocity analysis (https://velocyto.org). Consequently, CellOracle preprocesses the scRNA-seq data per Velocyto requirements by first filtering the genes and imputing dropout. Dropout can affect Velocyto’s transition probability calculations; thus, *k*-nearest neighbour (KNN) imputation must be performed before the simulation step.
(ii) Within-network signal propagation
  This step aims to estimate the effect of TF perturbation on cell identity. CellOracle simulates how a ‘shift’ in input TF expression leads to a ‘shift’ in its target gene expression and uses a partial derivative $\frac{\partial {x}_{j}}{\partial {x}_{i}}$. As we use a linear model, the derivative $\frac{\partial {x}_{j}}{\partial {x}_{i}}$ is a constant value and already calculated as *b*_*i*,*j*_ in the previous step if the gene *j* is directly regulated by gene *i*:
  $$\frac{\partial {x}_{j}}{\partial {x}_{i}}={b}_{i,j}.$$
  And we calculate the shift of target gene ${\Delta x}_{j}$ in response to the shift of regulatory gene ${\Delta x}_{i}$:
  $${\Delta x}_{j}=\frac{\partial {x}_{j}}{\partial {x}_{i}}{\Delta x}_{i}={b}_{i,j}{\Delta x}_{i}.$$
  As we want to consider the gene-regulatory ‘network’, we also consider indirect connections. The network edge represents a differentiable linear function shown above, and the network edge connections between indirectly connected nodes is a composite function of the linear models, which is differentiable accordingly. Using this feature, we can apply the chain rule to calculate the partial derivative of the target genes, even between indirectly connected nodes.
  $$\frac{\partial {x}_{j}}{\partial {x}_{i}}=\mathop{\prod }\limits_{k=0}^{n}\frac{\partial {x}_{k+1}}{\partial {x}_{k}}=\mathop{\prod }\limits_{k=0}^{n}{b}_{k,k+1},$$
  where
  $$\begin{array}{c}{x}_{k}\in \{{x}_{0},\,{x}_{1},\,\ldots {x}_{n}\}\,=\,\text{Gene expression of ordered network}\\ \,\text{nodes on the shortest path from gene}\,i\,\text{to gene}\,j.\end{array}$$
  For example, when we consider the network edge from gene 0 to 1 to 2, the small shift of gene 2 in response to gene 0 can be calculated using the intermediate connection with gene 1 (Supplementary Fig. 1).
  $$\frac{\partial {x}_{2}}{\partial {x}_{0}}=\frac{\partial {x}_{1}}{\partial {x}_{0}}\times \frac{\partial {x}_{2}}{\partial {x}_{1}}={b}_{0,1}\times {b}_{1,2}$$
  $${\Delta x}_{2}=\frac{\partial {x}_{2}}{\partial {x}_{0}}{\Delta x}_{0}={b}_{0,1}{b}_{1,2}{\Delta x}_{0}$$
  In summary, the small shift of the target gene can be formulated by the multiplication of only two components, GRN model coefficient *b*_*i*,*j*_ and input TF shift ${\Delta x}_{i}$. In this respect, we focus on the gradient of gene expression equations rather than the absolute expression values so that we do not model the error or the intercept of the model, which potentially includes unobservable factors within the scRNA-seq data.
  The calculation above is implemented as vector and matrix multiplication. First, the linear regression model can be shown as follows.
  $${X}^{{\prime} }=X\cdot B+C,$$
  where the $X\in {{\mathbb{R}}}^{1\times N}$ is a gene expression vector containing *N* genes, $C\in {{\mathbb{R}}}^{1\times N}$ is the intercept vector, $B\in {{\mathbb{R}}}^{N\times N}$ is the network adjacency matrix, and each element *b*_*i,j*_ is the coefficient value of the linear model from regulatory gene *i* to target gene *j*.
  First, we set the perturbation input vector $\Delta {X}_{{\rm{input}}}\in {{\mathbb{R}}}^{1\times N}$, a sparse vector consisting of zero except for the perturbation target gene *i*. For the TF perturbation target gene, we set the shift of the TF to be simulated. The CellOracle function will produce an error if the user enters a gene shift corresponding to an out-of-distribution value.
  Next, we calculate the shift of the first target gene:
  $$\Delta {X}_{{\rm{simulated}},n=1}=\Delta {X}_{{\rm{input}}}\cdot B.$$
  However, we fix the perturbation target gene *i* value, and the ${\Delta x}_{i}$ retains the same value as the input state. Thus, the following calculation will correspond to both the first and the second downstream gene shift calculations.
  $$\Delta {X}_{{\rm{simulated}},n=2}=\Delta {X}_{{\rm{simulated}},n=1}\cdot B.$$
  Likewise, the recurrent calculation is performed to propagate the shift from gene to gene in the network. Repeating this calculation for *n* iterations, we can estimate the effects on the first to the *n*th indirect target gene (Extended Data Fig. 1b–d):
  $$\Delta {X}_{{\rm{simulated}},n}=\Delta {X}_{{\rm{simulated}},n-1}\cdot B.$$
  CellOracle performs three iterative cycles in the default setting, sufficient to predict the directionality of changes in cell identity (Supplementary Figs. 4 and 5). We avoid a higher number of iterative calculations as it might lead to unexpected behaviour. Of note, CellOracle performs the calculations cluster-wise after splitting the whole GEM into gene expression submatrices on the basis of the assumption that each cluster has a unique GRN configuration. Also, gene expression values are checked between each iterative calculation to confirm whether the simulated shift corresponds to a biologically plausible range. If the expression value for a gene is negative, this value is adjusted to zero. The code in this step is implemented from scratch, specifically for CellOracle perturbations using NumPy, a python package for numerical computing (https://numpy.org).
(iii) Estimation of transition probabilities
  From the previous steps, CellOracle produces a simulated gene expression shift vector $\Delta {X}_{{\rm{simulated}}}\in {{\mathbb{R}}}^{1\times N}$ representing the simulated initial gene expression shift after TF perturbation. Next, CellOracle aims to project the directionality of the future transition in cell identity onto the dimensional reduction embedding (Fig. 1a, right and Extended Data Fig. 1e). For this task, CellOracle uses a similar approach to Velocyto (https://github.com/velocyto-team/velocyto.py). Velocyto visualizes future cell identity on the basis of the RNA-splicing information and calculated vectors from RNA synthesis and degradation differential equations. CellOracle uses the simulated gene expression vector $\Delta {X}_{{\rm{simulated}}}$ instead of RNA-velocity vectors.
  First, CellOracle estimates the cell transition probability matrix $P\in {{\mathbb{R}}}^{M\times M}$ (*M* is number of cells): *p*_*i*,*j*_, the element in the matrix *P*, is defined as the probability that cell *i* will adopt a similar cell identity to cell *j* after perturbation. To calculate *p*_*i*,*j*_, CellOracle calculates the Pearson’s correlation coefficient between *d*_*i*_ and *r*_*i*,*j*_:
  $${p}_{ij}=\frac{\exp \left(corr\left({r}_{ij}{,d}_{i}\right)/T\right)}{\sum _{j\in G}\exp \left(corr\left({r}_{ij}{,d}_{i}\right)/T\right)},$$
  where *d*_*i*_ is the simulated gene expression shift vector $\Delta {X}_{{\rm{simulated}}}\in {{\mathbb{R}}}^{1\times N}$ for cell *i*, and ${r}_{ij}\in {{\mathbb{R}}}^{1\times N}$ is a subtraction of the gene expression vector $X\in {{\mathbb{R}}}^{1\times N}$ between cell *i* and cell *j* in the original GEM. The value is normalized by the Softmax function (default temperature parameter *T* is 0.05). The calculation of *p*_*i.j*_ uses neighbouring cells of cell i. The KNN method selects local neighbours in the dimensional reduction embedding space (*k* = 200 as default).
(iv) Calculation of simulated cell-state transition vector
  The transition probability matrix *P* is converted into a transition vector ${V}_{i,{\rm{simulated}}}\in {{\mathbb{R}}}^{1\times 2}$, representing the relative cell-identity shift of cell *i* in the 2D dimensional reduction space, as follows: CellOracle calculates the local weighted average of vector ${V}_{i,j}\in {{\mathbb{R}}}^{1\times 2},{V}_{i,j}$ denotes the 2D vector obtained by subtracting the 2D coordinates in the dimensional reduction embedding between cell *i* and cell *j* (${\rm{cell}}\;j\in G$).
  $${V}_{i,{\rm{s}}{\rm{i}}{\rm{m}}{\rm{u}}{\rm{l}}{\rm{a}}{\rm{t}}{\rm{e}}{\rm{d}}}=\,\sum _{j\in G}{p}_{ij}{V}_{i,j}$$
(v) Calculation of vector field
  The single-cell resolution vector ${V}_{i,{\rm{simulated}}}$ is too fine to interpret the results in a large dataset consisting of many cells. We calculate the summarized vector field using the same vector averaging strategy as Velocyto. The simulated cell-state transition vector for each cell is grouped by grid point to get the vector field, ${V}_{{\rm{vector}}{\rm{field}}}=\,{{\mathbb{R}}}^{2\times L\times L}$, (*L* is grid number, default *L* is 40). ${v}_{{\rm{grid}}}\in {{\mathbb{R}}}^{2}$, an element in the ${V}_{{\rm{vector}}{\rm{field}}}$, is calculated by the Gaussian kernel smoothing.
  $${v}_{{\rm{grid}}}={\sum }_{i\in H}{K}_{\sigma }(g,\,{V}_{i,{\rm{simulated}}}){V}_{i,{\rm{simulated}}},$$
  where the $g\in {{\mathbb{R}}}^{2}$ denotes grid point coordinates, *H* is the neighbour cells of *g* and ${K}_{\sigma }$ is the Gaussian kernel weight:
  $${K}_{\sigma }({v}_{0},{v}_{1})=\exp \left(\frac{-{\parallel {v}_{0}-{v}_{1}\parallel }^{2}}{2{\sigma }^{2}}\right).$$

### Calculation of pseudotime gradient vector field and inner-product score to generate a perturbation score

To aid the interpretation of CellOracle simulation results, we quantify the similarity between the differentiation vector fields and KO simulation vector fields by calculating their inner-product value, which we term the perturbation score (PS) (Extended Data Fig. 4). Calculation of the PS includes the following steps:

(i) Differentiation pseudotime calculation
  Differentiation pseudotime is calculated using DPT, a diffusion-map-based pseudotime calculation algorithm, using the scanpy.tl.dpt function (Extended Data Fig. 4a, left). CellOracle also works with other pseudotime data, such as Monocle pseudotime and URD pseudotime data. For the Farrell et al.^32^ zebrafish scRNA-seq data analysis, we used pseudotime data calculated by the URD algorithm, as described previously^32^.
(ii) Differentiation vector calculation based on pseudotime data
  The pseudotime data are transferred to the *n* by *n* 2D grid points (*n* = 40 as default) (Extended Data Fig. 4a, centre). For this calculation, we implemented two functions in CellOracle: KNN regression and polynomial regression for the data transfer. We choose polynomial regression when the developmental branch is a relatively simple bifurcation, as is the case for the Paul et al.^16^ haematopoiesis data. We used KNN regression for a more complex branching structure, such as the Farrell et al.^32^ zebrafish development data. Then, CellOracle calculates the gradient of pseudotime data on the 2D grid points using the numpy.gradient function, producing the 2D vector map representing the direction of differentiation (Extended Data Fig. 4a, right).
(iii) Inner-product value calculation between differentiation and KO simulation vector field
  Then, CellOracle calculates the inner-product score (perturbation score (PS)) between the pseudotime gradient vector field and the perturbation simulation vector field (Extended Data Fig. 4b). The inner product between the two vectors represents their agreement (Extended Data Fig. 4c), enabling a quantitative comparison of the directionality of the perturbation vector and differentiation vector with this metric.
(iv) PS calculation with randomized GRN model to calculate PS cut-off value

CellOracle also produces randomized GRN models. The randomized GRNs can be used to generate dummy negative control data in CellOracle simulations. We calculated cut-off values for the negative PS analysis in the systematic KO simulation. First, the negative PS is calculated for all TFs using either a normal or a randomized vector. The score distribution generated from the randomized vector was used as a null distribution. We determined the cut-off value corresponding to a false-positive rate of 0.01 by selecting the 99th percentile value of PSs generated with randomized results (Extended Data Fig. 3g).

### Network analysis

In addition to CellOracle’s unique gene perturbation simulation, CellOracle’s GRN model can be analysed with general network structure analysis methods or graph theory approaches. Before this network structure analysis, we filter out weak or insignificant connections. GRN edges are initially filtered on the basis of *P* values and absolute values of edge strength. The user can define a custom value for the thresholding according to the data type, data quality and aim of the analysis. After filtering, CellOracle calculates several network scores: degree centrality, betweenness centrality and eigenvector centrality. It also assesses network module information and analyses network cartography. For these processes, CellOracle uses igraph (https://igraph.org).

### Validation and benchmarking of CellOracle GRN inference

To test whether CellOracle can correctly identify cell-type- or cell-state-specific GRN configurations, we benchmarked our new method against diverse GRN inference algorithms: WGCNA, DCOL, GENIE3 and SCENIC. WGCNA is a correlation-based GRN inference algorithm, which is typically used to generate a non-directional network^53^; DCOL is a ranking-based non-linear network modelling method^54^; and GENIE3 uses an ensemble of tree-based regression models, and aims to detect directional network edges. GENIE3 emerged as one of the best-performing algorithms in a previous benchmarking study^55^. The SCENIC algorithm integrates a tree-based GRN inference algorithm with information on TF binding^7^.

### Preparation of input data for GRN inference

We used the Tabula Muris scRNA-seq dataset for GRN construction input data^56^. Cells were subsampled for each tissue on the basis of the original tissue-type annotation: spleen, lung, muscle, liver and kidney. Data for each tissue were processed using the standard Seurat workflow, including data normalization, log transformation, finding variable features, scaling, principal component analysis (PCA) and Louvain clustering. The data were downsampled to 2,000 cells and 10,000 genes using highly variable genes detected by the corresponding Seurat function. Cell and gene downsampling were necessary to run the GRN inference algorithms within a practical time frame: we found that some GRN inference algorithms, especially GENIE3, take a long time with a large scRNA-seq dataset, and GENIE3 could not complete the GRN inference calculation even after several days if the whole dataset was used.

### GRN inference method

After preprocessing, the exact same data were subjected to each GRN inference algorithm to compare results fairly. We followed the package tutorial and used the default hyperparameters unless specified otherwise. Details are as follows. WGCNA: we used WGCNA v.1.68 with R 3.6.3. WGCNA requires the user to select a ‘power parameter’ for GRN construction. We first calculate soft-thresholding power using the ‘pickSoftThreshold’ function with networkType=“signed”. Other hyperparameters were set to default values. Using the soft-thresholding power value, the ‘adjacency’ function was used to calculate the GRN adjacency matrix. The adjacency matrix was converted into a linklist object by the ‘getLinkLis’ function and used as the inferred value of the WGCNA algorithm. DCOL: we used nlnet v.1.4 with R 3.6.3. The ‘nlnet’ function was used with default parameters to make the DCOL network. The edge list was extracted using the ‘as_edgelist’ function. DCOL infers an undirected graph without edge weights. We assigned the value 1.0 for the inferred network edge and 0.0 for other edges. The assigned value was used as the output of the DCOL algorithm. GENIE3: we used GENIE3 v.1.8.0 with R 3.6.3. The GRN weight matrix was calculated with the processed scRNA-seq data using the ‘GENIE3’ function and converted into a GRN edge and weight list by the ‘getLinkList’ function. GENIE3 provides a directed network with network weight. The weight value was directly used as the inferred value of the GENIE3 algorithm. SCENIC: we used SCENIC v.1.2.2 with R 3.6.3. The SCENIC GRN calculation involves multiple processes. The calculation was performed according to SCENIC’s tutorial (https://rdrr.io/github/aertslab/SCENIC/f/vignettes/SCENIC_Running.Rmd). First, we created the initialize settings configuration object with ‘initializeScenic’. Then we calculated the co-expression network using the ‘runGenie3’ function, following the GRN calculation with several SCENIC functions; runSCENIC_1_coexNetwork2modules, runSCENIC_2_createRegulons and runSCENIC_2_createRegulons. We used the ‘10kb’ dataset for the promoter information range. The calculated GRN information was loaded with the ‘loadInt’ function, and the ‘CoexWeight’ value was used as the inferred value of the SCENIC algorithm.

### Ground-truth data preparation for GRN benchmarking

Cell-type-specific ground-truth GRNs were generated in the same manner as in a previous benchmarking study^55^. Here, we selected tissues commonly available in the Tabula Muris scRNA-seq dataset, mouse sci-ATAC-seq atlas data and ground-truth datasets: heart, kidney, liver, lung and spleen. The ground-truth data were constructed as follows. (i) Download all mouse TF ChIP–seq data as bed files from the ChIP-Atlas database (https://chip-atlas.org). (ii) Remove datasets generated under non-physiological conditions. For example, we removed ChIP–seq data from gene KOs or adeno-associated virus treatment. (iii) Remove data that include fewer than 50 peaks. (iv) Select peaks detected in multiple studies. (v) Group data by TF and remove TFs if the number of detected target genes is less than ten peaks. (vi) Convert data into a binary network: each network edge is labelled either 0 or 1, representing its ChIP–seq binding between genes. These steps yielded tissue- or cell-type-specific ground-truth data for 80 TFs, corresponding to 1,298 experimental datasets.

### GRN benchmarking results

GRN inference performance was evaluated by the AUROC and the early precision ratio (EPR), following the evaluation method used in a previous benchmarking study^55^. CellOracle and SCENIC outperformed WGCNA, DCOL and GENIE3 based on AUROC (Extended Data Fig. 2a). This is because CellOracle and SCENIC filter out non-transcriptional connections (that is, non-TF–target gene connections) and other methodologies detect many false-positive edges between non-TFs. CellOracle with a scATAC-seq atlas base GRN performed better than CellOracle with a promoter base GRN and SCENIC. This difference was mainly derived from sensitivity (or true-positive rate). With scATAC-seq data, CellOracle captures a higher number of regulatory candidate genes. Considering EPR, representing inference accuracy for top *k* network edges (*k* = number of network edges with the label ‘1’ in the ground-truth data), CellOracle performed well compared to other approaches (Extended Data Fig. 2b): GENIE3 and WGCNA assigned a high network edge weight to many non-transcriptional connections, resulting in many false-positive edges for the highly ranked inferred genes.

The CellOracle GRN construction method was analysed further to assess the contribution of the base GRN. We performed the same GRN benchmarking with a scrambled motif base GRN or no base GRN. For the scrambled motif base GRN, we used scrambled TF-binding-motif data for the base GRN construction. For the no base GRN analysis, selection of regulatory candidate genes was skipped, and all genes were used as regulatory candidate genes. As expected, the AUROC scores decreased when we used the scrambled motif base GRN (ranked 12/13 in AUROC, 11/13 in EPR; Extended Data Fig. 2a,b), decreasing even further in the no base GRN model (13/13; Extended Data Fig. 2a,b). The scrambled motif base GRN did not detect many regulatory candidate TFs, producing lower sensitivity. However, the scrambled motif base GRN can still work positively by removing connections from non-TF genes to TFs, functioning to filter out false-positive edges, and resulting in a better score relative to the no base GRN model. In summary, the base GRN is primarily important to achieve acceptable specificity, and the scATAC-seq base GRN increases sensitivity.

Next, we used CellOracle after downsampling cells to test how cell number affects GRN inference results. Cells were downsampled to 400, 200, 100, 50, 25 and 10 cells and used for GRN analysis with the scATAC-seq base GRN. GRNs generated with 400, 200, 100 and 50 cells received comparable or slightly reduced AUROC scores. The AUROC score decreased drastically for GRNs generated with 25 and 10 cells (Extended Data Fig. 2c). EPR was relatively robust even with small cell numbers (Extended Data Fig. 2d).

We performed additional benchmarking to investigate data compatibility between the base GRN and scRNA-seq data sources. A tissue-specific base GRN was generated separately using bulk ATAC-seq data^57^. We focused on the same five tissue types as above. Unprocessed bulk ATAC-seq data were downloaded from the NCBI database using the SRA tool kit (spleen: SRR8119827; liver: SRR8119839; heart: SRR8119835; lung: SRR8119864; and kidney: SRR8119833). After FASTQC quality check (https://www.bioinformatics.babraham.ac.uk/projects/fastqc/), fastq files were mapped to the mm9 reference genome and converted into bam files. Peak calling using HOMER was used to generate bed files from the bam files. Peak bed files were then annotated with HOMER. Peaks within 10 kb around the TSS were used. Peaks were sorted by the ‘findPeaks Score’ generated by the HOMER peak-calling step, and we used the top 15,000 peaks for base GRN construction. These peaks were scanned with the gimmemotifs v.5 vertebrate motif dataset, which is the same motif set we use for scATAC-seq base GRN construction.

We compared benchmarking scores between GRN inference results generated from different base GRNs. Overall, GRN construction performed best when the same tissue type for ATAC-seq base GRN construction and scRNA-seq was used (10/13 in AUROC, 11/13 in EPR; Extended Data Fig. 2e,f). The score was lower with different tissue types combined between the base GRN and scRNA-seq data. In summary, benchmarking confirmed that our GRN construction method performs well for the task of transcriptional GRN inference.

### CellOracle evaluation

#### Evaluation of simulation value distribution range

We investigated a range of simulated values to confirm that the signal propagation step does not generate an out-of-distribution prediction. Specifically, we assessed the distribution of the sum of the simulated shift and original gene expression, which correspond to the simulated expression level (termed ‘simulation gene expression level’ here for explanatory purposes: *X*_simulation gene expression level_ = *X*_original_ + Δ*X*_simulated,_). We evaluate all genes, comparing the simulation gene expression level with the original gene expression distribution. To detect out-of-distribution data, we calculated the maximum exceedance percentage, representing the percentage difference of the maximum value of the simulated gene expression level compared to the maximum value of the wild-type gene expression value. The higher maximum exceedance indicates a bigger difference between simulated and wild-type values, identifying out-of-distribution values. For the *Spi1* KO simulation with the Paul et al. haematopoiesis dataset^16^, we present the top four genes showing the maximum exceedance values (Supplementary Fig. 2). The simulation expression levels of even these genes appear very similar to the original wild-type distributions of gene expression. For example, in the *Ly86* simulated value distribution, 99.963% of all cells are within the wild-type gene expression range. Only 0.037% of cells exhibit a *Ly86* gene simulation value outside the wild-type distribution, but the maximum difference is only 3.2%. We designed CellOracle to simulate a minimal relative shift vector rather than an out-of-distribution prediction, confirmed by this analysis. The functions we have used for these analyses are implemented in CellOracle. Users can check simulation value distributions, and CellOracle will produce a warning if out-of-distribution simulations occur.

To further explore the minimum number of cells with minor out-of-distribution values, we generated a simulation vector in which the out-of-distribution values are clipped into the wild-type distribution range. The simulated cell-identity shift vector of clipped values is indistinguishable compared to the original results (Supplementary Fig. 2b–e), confirming that the CellOracle simulation is not relying on these out-of-distribution values. The out-of-distribution value can be clipped if we add ‘clip_delta_X=True’ in the CellOracle signal propagation function. Thus, users can ensure the simulation is not relying on out-of-distribution values.

### CellOracle simulation results generated with randomized GRN or no signal propagation

We performed KO simulation with randomized GRN models to clarify the necessity of the GRN signal propagation simulation. In addition, we calculated cell-identity vectors without the signal propagation step; the cell-identity shift vector was calculated solely on the basis of input TF expression loss, thus representing the information from the expression pattern of only a single TF. The vector map in Supplementary Fig. 3 shows *Gata1* KO simulation results and *Spi1* KO simulation results with an intact GRN coefficient matrix, randomized GRN matrix or no GRN signal propagation. The randomized GRN analysis results and no GRN signal propagation results show only slight cell-identity shift vectors (Supplementary Fig. 3b,c,e,f). Although very subtle vectors can be observed, most expected simulation results are not obtained. Thus, we confirmed that the GRN signal propagation strategy has an essential role in the CellOracle KO simulation.

### Evaluation of signal propagation number

We next tested the number of iterations at the signal propagation step. We performed KO simulations using two independent mouse haematopoiesis datasets: Paul et al.^16^ and Dahlin et al.^58^. For several TFs, we tested different numbers of signal propagation rounds in the KO simulations across independent clusters. First, focusing on the Paul dataset, simulation vector fields for *Spi1* and *Gata1*, with 0, 1 and 3 rounds of signal propagation, were investigated (Supplementary Fig. 4). The simulation under hyperparameter *n* = 0 shows the vector calculated without any signal propagation within the GRN; that is, the vector is calculated from only the difference of the input TF gene expression shift. This *n* = 0 simulation shows almost no phenotype, showing the necessity of the GRN signal propagation process. Next, a comparison of vector fields from *n* = 1 and *n* = 3 simulations shows similar results. This is not surprising given the following. (1) Most coefficient values in the GRN are small, ranging between −1 and 1 (Supplementary Fig. 4d). (2) Accordingly, the signal will be attenuated over the propagation process in most cases. (3) This also means that the first signal propagation step will produce the most significant shifts relative to the later steps. However, when scrutinizing the vectors, we observe a more evident shift in cell identity around the late GMP cluster and the early granulocytes in the *n* = 3 *Gata1* KO vectors compared to *n* = 1 vectors. The results suggest that the second and third rounds of signal propagation increase the sensitivity to detect small shifts by adding the second and third rounds of downstream gene effects, respectively.

To quantify these observations and determine whether there is an ideal number of signal propagation rounds, we investigated the L1-norm of Δ*X*, representing the sum of the magnitudes of each simulated gene expression shift. The L1-norm of Δ*X* is almost saturated at the *n* = 3 in most cases (Supplementary Fig. 4c). We also performed these analyses with the Dahlin haematopoiesis dataset^58^ (Supplementary Fig. 5). Overall, the results are consistent with our analysis of the Paul data. Again, we observe that the L1-norm of Δ*X* is saturated at relatively small *n* values in most cases. However, *Cebpa* is an outlier in this analysis, in which the delta *X* length gradually and continuously increases as *n* increases. We next examined the vector field of *Cebpa* with various *n* (Supplementary Fig. 6). Despite such divergence of the L1-norm of Δ*X*, the vector field of *Cebpa* showed consistent results, suggesting that the calculation strategy for cell-identity shift is robust using the local neighbour vectors (Extended Data Fig. 1e).

Altogether, at *n* = 3, the simulated shift vectors almost converge, producing consistent results. In rare cases, the L1-norm of Δ*X* might show divergence with *n*. However, the *n* = 3 simulation results are consistent with higher *n* values, which might generate unexpected behaviour owing to signal divergence. On the basis of these analyses, we recommend that users perform three iterations for the signal propagation step.

### Selection of dimensionality reduction method

#### CellOracle simulation with UMAP and *t*-SNE using Paul et al. haematopoiesis data

We used UMAP and *t*-distributed stochastic neighbour embedding (*t*-SNE) for the perturbation simulation analysis to show how the choice of dimensionality reduction affects CellOracle results. We used Scanpy to construct UMAP or *t*-SNE plots using the Paul et al. haematopoiesis dataset^16^. In the UMAP (Supplementary Fig. 7a), we observe a similar trajectory that agrees with the force-directed graph (Fig. 1b). However, monocyte and granulocyte branches on the UMAP are relatively less resolved. This notwithstanding, the simulation results using the UMAP (Supplementary Fig. 8, top) lead to the same conclusion as Fig. 1. For example, in the *Gata1* KO simulation, we correctly predict inhibited differentiation along the MEP lineage whereas GMP differentiation is promoted. Furthermore, we predict inhibited GMP to granulocyte differentiation, consistent with our force-atlas-based presentation in Fig. 1h. In comparison, the overall structure of the *t*-SNE graph is consistent with the force-directed and UMAP graphs, although it lacks resolution (Supplementary Fig. 7b). However, the *t*-SNE results still agree with Fig. 1, just at a lower resolution (Supplementary Fig. 8, bottom). In conclusion, we stress that the choice of the dimensional reduction algorithm is crucial to sensitively analyse the cell differentiation trajectory.

#### Guidance for selecting the dimensionality reduction method

For the force-directed graph calculation, we recommend using Scanpy’s sc.pl.draw_graph function^59^ or SPRING^60^. Both internally use force atlas 2 (ref. ^61^). Compared to UMAP, force-directed graphs can capture more fine-branching structures but can be unstable if the data have many branches that can overlap. To avoid branch overlap, PAGA cell trajectory information can be used to initiate the force-directed graph calculation: https://scanpy.readthedocs.io/en/stable/tutorials.html#https://github.com/theislab/paga.

We recommend using force-directed graphs as a first choice because they generally produce a high-resolution lineage structure. However, we recommend UMAP as a reliable alternative if overlapping branches are observed. In our CellOracle tutorial, we show the detailed guide and code for the dimensionality reduction implementation, including data preprocessing: https://morris-lab.github.io/CellOracle.documentation.

### CellOracle KO simulation with unrelated cell-type base GRNs

To assess how base GRN performance relates to scATAC data source, we performed TF KO simulations in haematopoiesis using the ‘general’ mouse scATAC-seq atlas^13^ base GRN versus a heart-specific base GRN to represent an unrelated cell type (Supplementary Fig. 9). The simulation vectors using the mismatched heart base GRN are weaker, although still in general agreement. We reason that even if the base GRN retains some edges that are not active in the scRNA-seq data, CellOracle can still work robustly. However, simulation with the heart base GRN fails to detect the early granulocyte phenotype in the *Gata1* KO and almost all shifts in the *Cepba* KO, suggesting reduced sensitivity with the mismatched base GRN.

We also assess the mean degree centrality (the number of genes to which a TF is connected) in the inferred GRNs for each of four TFs (Supplementary Fig. 10). With the inappropriate heart base GRN, CellOracle fails to build network edges for some genes, resulting in a low degree centrality score and reduced simulation sensitivity. We recommend starting CellOracle analysis with the general GRN and comparing its performance against tailored base GRNs.

### Markov simulation based on CellOracle simulation vector

To estimate cell distribution in response to gene perturbation, we need to consider both the differentiation hierarchy and the perturbation vector together. We performed a Markov random walk simulation as described previously^52^ (https://github.com/velocyto-team/velocyto.py) with some modifications. First, our Markov simulation used the CellOracle cell-identity transition vector in addition to the differentiation vector; the transition probability matrix for these vectors was applied alternatively to consider both effects. Second, cells in early differentiation stages were selected and used for the initial state of our Markov simulation, whereas the previous study used the whole population as the initial state^52^. The Markov simulation analysis with data from another study^59^ is shown in Supplementary Fig. 17 to show typical simulation results and their interpretation.

### CellOracle analysis with previously published scRNA-seq and scATAC-seq data

#### Paul et al. mouse haematopoiesis scRNA-seq data

The GEM was downloaded with Scanpy’s data loading function, scanpy.datasets.paul15(). After removing genes with zero counts, the GEM was normalized by total UMI counts ((scanpy.pp.filter_genes(min_counts=1), scanpy.pp.normalize_per_cell(key_n_counts=‘n_counts_all’)). Highly variable genes, including 90 TFs, detected by scanpy.pp.filter_genes_dispersion(flavor=‘cell_ranger’, n_top_genes=2000, log=False), were used for the following downstream analysis: the GEM was log-transformed, scaled and subjected to PCA (scanpy.pp.log1p(), scanpy.pp.scale(), scanpy.tl.pca(svd_solver=‘arpack’)). We calculated the force-directed graph dimensional reduction data based on the PAGA graph^62^ for initialization (scanpy.tl.paga(), scanpy.tl.draw_graph(init_pos=‘paga’)). Cells were clustered using the Louvain clustering method (scanpy.tl.louvain (resolution=1.0)). Clusters were annotated manually using marker gene expression and the previous annotations from Paul et al.^16^ We removed dendritic cell (DC) and lymphoid cell clusters to focus on myeloid cell differentiation. GRN calculation and simulation were performed as described above, using the default parameters. For the base GRN, we used the base GRN generated from the mouse sci-ATAC-seq atlas dataset^13^.

Cell density was visualized using a kernel density estimation (KDE) plot. First, we performed random downsampling to 768 cells to adjust the cell number between WT and KO samples. KDE was calculated with the scipy.stat.gaussian_kde function. The calculated KDE was visualized with the matplotlib.pyplot.contour function. We used the same contour threshold levels between all samples.

Although we did not focus on the network structure in the main text, we examined CellOracle GRN models using graph theory approaches before the simulation analysis. Graph theory analysis revealed that these inferred GRN configurations resemble a scale-free network the degree distribution of which follows a power law, a characteristic configuration of biological networks^63^ (Extended Data Fig. 3b). Further, we assess GRNs using degree centrality—a basic measure of how many genes a TF connects to^63^. Using the MEP cluster as an example, 27 out of 30 genes with a high degree centrality score in the MEP_0 GRN are confirmed known regulators of MEP lineage differentiation or stem and progenitor cell function (Extended Data Fig. 3c and Supplementary Table 2). Analysis of additional clusters yielded similar agreement with previous literature, confirming that CellOracle GRN inference captures biologically plausible cell-state-specific GRN structures, consistent with previous biological knowledge. All network analysis and simulation results can be explored at https://www.celloracle.org.

#### Pijuan-Sala et al. mouse early gastrulation and organogenesis scRNA-seq data

We applied CellOracle to a scRNA-seq atlas of mouse gastrulation and organogenesis by Pijuan-Sala et al.^30^. This single-cell profiling of WT cells highlighted a continuous differentiation trajectory across the early development of various cell types (Extended Data Fig. 9a). In addition, the developmental effects of *Tal1* KO, a TF known to regulate early haematoendothelial development^64,65^, were investigated in this study. We validated the CellOracle simulation using these *Tal1* KO ground-truth scRNA-seq data. The data were generated from seven chimeric E8.5 embryos of WT and *Tal1* KO cells (25,307 cells and 26,311 cells, respectively). We used the R library, MouseGastrulationData (https://github.com/MarioniLab/MouseGastrulationData), to download the mouse early gastrulation scRNA-seq dataset. This library provides the GEM and metadata. We used the *Tal1* chimera GEM and cell-type annotation, “cell type.mapped”, provided by this library. Data were normalized with SCTransform^66^. The GEM was converted to the AnnData format and processed in the same way as the Paul et al. dataset. For the dimensionality reduction, we used UMAP using the PAGA graph for the initialization (maxiter=500, min_dist=0.6). We removed the extraembryonic ectoderm (ExE), primordial germ cell (PGC) and stripped nuclei clusters which lie outside the main differentiation branch. After removing these clusters, we used the WT cell data for the simulations (24,964 cells). GRN calculations and simulations were performed as described above using the default parameters. We used the base GRN generated from the mouse sci-ATAC-seq atlas dataset. We constructed cluster-wise GRN models for 25 cell states. Then, we simulated *Tal1* KO effects using the WT scRNA-seq dataset. For the late-stage-specific *Tal1* conditional KO simulation, we set *Tal1* expression to be zero in the blood progenitor and erythroid clusters to analyse the role of *Tal1* in late erythroid differentiation stages (Extended Data Fig. 9i,j).

#### Farrell et al. zebrafish early development scRNA-seq data

GEM, metadata and URD trajectory data were downloaded from the Broad Institute Single Cell Portal (https://tinyurl.com/7dup3b5k). We used the cell clustering data and developmental lineage data from Farrell et al.^32^ The GEM was already normalized and log_2_-transformed, which we converted to non-log-transformed data before CellOracle analysis. The GEM had human gene symbols, which we converted back to zebrafish gene symbols using gene name data in ZFIN (https://zfin.org). We used URD dimensional reduction embedding data. To use the URD differentiation trajectory in the CellOracle simulations, we ran several preprocessing and calculations. We first identified cells with URD coordinate data (*n* = 26,434 cells). The “EPL/periderm and primordial germ cell” cluster, which represents 1.7% of the total population, was also excluded from our analysis because it is located outside the main differentiation trajectory branch. The whole URD structure (*n* = 25,711 cells) was split into four sub-branches to simplify the calculations (Extended Data Fig. 10b). Then, we converted the original URD coordinates, a 3D matrix, into a 2D matrix using PCA (sklearn.decomposition.PCA) because CellOracle requires 2D dimensional reduction embedding data. The GEM was converted into the AnnData format. At the variable gene detection step, we selected the top 3,000 genes. GRN calculation and simulations were performed as described above using the default parameters. We did not calculate pseudotime because the pseudotime data calculated with URD were available. The pre-calculated pseudotime data were used to calculate the 2D development vector field. For base GRN construction, we used UCSC TSS and promoter data and the zebrafish reference genome (https://useast.ensembl.org/Danio_rerio/Info/Index), danRer11 (the bed file is included in the CellOracle package). The promoter DNA sequence was scanned with CisBP version2 motif dataset to generate the base GRN (http://cisbp.ccbr.utoronto.ca).

For screening novel regulators of axial mesoderm cell identity, we prioritized candidate genes as follows. First, we performed CellOracle KO simulations for 232 active TFs, which had at least one gene edge in the constructed GRN model in the axial mesoderm branch (Extended Data Fig. 12a, step 1). We focused on the early differentiation stage by selecting cells between digitized pseudotime 0 and 3 (Extended Data Fig. 12a, step 2). For this analysis, we focused on negative perturbation scores to identify candidate TFs. A large negative perturbation score indicates a predicted inhibition or block in differentiation following TF KO; thus, we reasoned that these TFs might have a positive role in differentiation (Extended Data Fig. 12a, step 3). To prioritize TFs according to the predicted differentiation inhibition effects, we ranked TFs according to the sum of their negative perturbation scores, resulting in the 30 genes listed in Fig. 5a. Next, we surveyed the GRN degree centrality scores of 30 candidate genes in the notochord cluster GRN because we reasoned that those genes with higher GRN degree centrality result in a more reliable simulation. Then, we calculated the gene specificity score comparing the axial mesoderm sub-branch and the other sub-branches using the Scanpy function, sc.tl.rank_genes_groups(). Although gene specificity does not necessarily relate to gene function, we assumed that specific gene expression would simplify the interpretation of experimental results and reduce the likelihood of unexpected phenotypes from clusters other than axial mesoderm. Finally, we analysed mean expression, assuming perturbation experiments with highly expressed genes would be more robust, especially in the CRISPR–Cas9-based F_0_ embryo analysis. After removing previously reported genes, we selected candidate genes that exist in the 50th percentile of these scores (Extended Data Fig. 12b, highlighted in a grey rectangle), resulting in *lhx1a*, *sebox*, *irx3a*, *creb3l1 and zic2a*. We finally selected three candidates, *lhx1a*, *sebox* and *irx3a*, and removed *creb3l1* and *zic2a* from the first LOF experiment list, according to the following rationale: *creb3l1* gene sequences are similar to *creb3l2*; thus, it was challenging to design specific sgRNAs to target this gene; *creb3l2* was previously reported to regulate axial mesoderm development. Although *zic2a* narrowly passed the gene specificity threshold described above, we found that *zic2a* expression was high in the other mesendoderm sub-branch and the ectoderm sub-branches; thus, we excluded this gene from our downstream analyses.

#### Dahlin et al. mouse haematopoiesis scRNA-seq data

Mouse haematopoiesis scRNA-seq data from Dahlin et al.^58^ were downloaded from the PAGA GitHub repository (https://github.com/theislab/paga). The GEM was normalized by total UMI counts after removing genes with zero counts ((scanpy.pp.filter_genes(min_counts=1), scanpy.pp.normalize_per_cell(key_n_counts=‘n_counts_all’)). Highly variable genes were detected and used for the following downstream analysis: (scanpy.pp.filter_genes_dispersion(flavor=‘cell_ranger’, n_top_genes=3000, log=False)). The GEM was log-transformed, scaled and subjected to PCA and Louvain clustering (scanpy.pp.log1p(), scanpy.pp.scale(), scanpy.tl.pca(svd_solver=‘arpack’), scanpy.tl.louvain(resolution=1.5)). The original force-directed graph reported in Dahlin et al.^58^ was used for the CellOracle simulation. GRN calculation and simulation were performed using the default parameters. For the base GRN, we used the mouse sci-ATAC-seq atlas dataset^13^.

#### Setty et al. human haematopoiesis scRNA-seq data

Human haematopoiesis scRNA-seq were downloaded from the Human Cell Atlas: https://data.humancellatlas.org/explore/projects/091cf39b-01bc-42e5-9437-f419a66c8a45 (Setty et al.)^67^. The GEM was normalized by total UMI counts after removing genes with zero counts ((scanpy.pp.filter_genes(min_counts=1), scanpy.pp.normalize_per_cell(key_n_counts=‘n_counts_all’)). Highly variable genes were detected and used for the following downstream analysis: (scanpy.pp.filter_genes_dispersion(flavor=‘cell_ranger’, n_top_genes=3000, log=False)). The GEM was log-transformed, scaled and subjected to PCA and Louvain clustering (scanpy.pp.log1p(), scanpy.pp.scale(), scanpy.tl.pca(svd_solver=‘arpack’), scanpy.tl.louvain(resolution=1.5)). The force-directed graph was calculated with SPRING (https://kleintools.hms.harvard.edu/tools/spring.html). We removed DC and lymphoid cell clusters in line with the Paul et al.^16^ data analysis. GRN calculation and simulation were performed using the default parameters. For the base GRN, we used the base GRN generated using the Buenrostro et al. human haematopoiesis scATAC-seq data described below^68^.

#### Buenrostro et al. human haematopoiesis scATAC-seq data

Human haematopoiesis scATAC-seq data from Buenrostro et al.^68^ were used to construct a human haematopoiesis base GRN. The scATAC-seq peak data and count matrix was obtained from the Gene Expression Omnibus (GEO), with accession code GSE96769, and processed with Cicero (v.1.3.4) to obtain co-accessibility scores as follows: After removing peaks with zero counts, cells were filtered by the peak count (min count = 200, max count = 30,000). The data were processed using Cicero functions (detect_genes(), estimate_size_factors(), preprocess_cds(method = "LSI"), reduce_dimension(reduction_method = ‘UMAP’, preprocess_method = "LSI")). Then Cicero co-accessibility scores were calculated using run_cicero() with human chromosome length information imported by data("human.hg19.genome"). Output peak and co-accessibility scores were used for CellOracle base GRN construction. CellOracle annotated the TSS site in the peaks, and the TSS peaks and *cis*-regulatory peaks with co-accessibility scores ≥ 0.8 were used for motif scanning. We used the gimmemotifs vertebrate v5 motif dataset, which is CellOracle’s default for mouse and human motif scanning.

TF motif enrichment analysis was performed using ChromVar^68^. The ChromVar score matrix, which includes 2,034 cells and 1,764 motif data, was processed with scanpy to generate a force-directed graph and Louvain clustering (scanpy.tl.pca(), scanpy.tl.louvain(resolution=0.5), scanpy.tl.draw_graph()). The cluster was annotated using cell source FACS gate sample labels. The score fold change was calculated and visualized as a volcano plot (Supplementary Fig. 16). The statistical test was performed using the two-tailed Wilcoxon rank-sum test with Bonferroni correction.

### Comparison between CellOracle haematopoiesis KO simulation results and previous reports

CellOracle KO simulation results for 12 key TFs that regulate myeloid differentiation are shown in Figs. 1 and 2, Extended Data Figs. 5 and 6 and Supplementary Figs. 13 and 14. The simulation results were compared with previous reports (summarized in Supplementary Table 2). In these figures, the summary of the simulation results is shown in the right column with the mark (*), which indicates that the simulation results agree with the previously reported role or phenotype of the TF. We note that the input haematopoiesis data focus on the myeloid lineage; thus, the simulation results show relative cell-identity shifts within the myeloid lineage only. For example, *Spi1* has an important role not only in the myeloid lineage but also in other cell types, such as HSCs and lymphoid lineages^69^. However, we cannot simulate the role in these cell types if they are not present in the input data.

1. *Klf1* (*KLF1*)
  *Klf1* promotes differentiation towards the ME lineage, promoting erythroid cell differentiation in particular^15^. CellOracle simulation results agree with this role (Extended Data Fig. 5a and Supplementary Figs. 13e and 14e).
2. *Gata1* (*GATA1*)
  *Gata1* promotes ME lineage differentiation and also promotes granulocyte differentiation^15,70^. Both the Paul et al.^16^ and Dahlin et al.^58^ data simulation results reproduce these *Gata1* roles. (Fig. 1f and Supplementary Fig. 13b). In the Setty et al. dataset^67^, the ME lineage phenotype is reproduced, but the granulocyte phenotype is not observed (Supplementary Fig. 14b). We speculate that this is because the Setty dataset includes few mature granulocytes.
3. *Gata2* (*GATA2*)
  *Gata2* is a key factor in maintaining stemness in MPPs^15^. Simulation results in all data agree with this role for *Gata2* (Extended Data Fig. 6a and Supplementary Figs. 13i and 14g).
4. *Spi1* (*SPI1*)
  *Spi1* promotes GM lineage differentiation. The inhibition of *Spi1* shifts cell identity from the GM to the ME lineage^15,71^. Simulation results in all datasets agree with this role of *Spi1* (Fig. 1e and Supplementary Figs. 13a and 14a).
5. *Cebpa* (*CEBPA*)
  Cebp*a* promotes GM lineage differentiation while inhibiting ME lineage differentiation^16,72^, and promoting granulocyte differentiation in particular^15^. Simulation results using the Paul et al.^16^ and Dahlin et al.^58^ datasets agree with this role for *Cebpa* (Fig. 2b and Supplementary Fig. 13c). Although the ME lineage phenotype is not detected using the Setty et al. dataset^67^, the GM lineage phenotype is successfully reproduced (Supplementary Fig. 14c).
6. *Cebpe* (*CEBPE*)
  *Cebpe* promotes granulocyte lineage differentiation^15,16^. Simulation results in all datasets agree with this role of *Cebpe* (Fig. 2c and Supplementary Figs. 13d and 14d).
7. *Gfi1* (*GFI1*)
  *Gfi1* promotes granulocyte lineage differentiation^15,72–74^. Simulation results using the Paul et al.^16^ and Dahlin et al.^58^ datasets agree with this role of *Gfi1* (Extended Data Fig. 5c and Supplementary Fig. 13g).
8. *Gfi1b* (*GFI1B*)
  *Gfi1b* promotes ME lineage differentiation^15^. Simulation results in all data suggest that *Gfi1b* promotes erythroid differentiation (Extended Data Fig. 5b and Supplementary Fig. 13f). The Mk phenotype is unclear in the simulation, probably owing to the small numbers of Mk cells.
9. *Irf8* (*IRF8*)
  *Irf8* promotes GM lineage differentiation. In particular, *Irf8* promotes monocyte differentiation as a lineage switch between monocyte and granulocyte bifurcation^29^. Simulation results in all data agree with the role of *Irf8* (Extended Data Fig. 5d and Supplementary Figs. 13h and 14f).
10. *Lmo2* (*LMO2*)
  *Lmo2* is a central factor in maintaining stemness in the MPP compartment^15^. Simulation results using the Dahlin et al. data^58^ agree with this role. (Supplementary Fig. 13l). However, simulation results using Paul et al. data^16^ showed a different phenotype in erythrocyte cells, suggesting that *Lmo2* is also crucial for promoting erythroid differentiation (Extended Data Fig. 6d). A function of *Lmo2* in promoting erythroid differentiation was also reported^75^.
11. *Runx1* (*RUNX1*)
  *Runx1* is an central factor in maintaining stemness in the MPP compartment^15^ Simulation results in all datasets agree with this role of *Runx1* (Extended Data Fig. 6b and Supplementary Fig. 13j).
12. *Fli1* (*FLI1*)

*Fli1* has context-dependent roles. *Fli1* is a key factor for Mk differentiation^15^, and for maintaining stemness in the stem and progenitor comparment^76^. The simulations consistently reproduce these phenotypes (Extended Data Fig. 6c and Supplementary Figs. 13k and 14h). In addition, a previous study reported that loss of *Fli1* causes dysregulation in later differentiation stages^77^, consistent in simulations using the Paul et al. dataset^16^ (Extended Data Fig. 6c).

### Zebrafish lines

The zebrafish experiments were approved by the Institutional Animal Care and Use Committees at Washington University in St Louis. All animal experiments followed all relevant guidelines and regulation. The following zebrafish lines were used in this study: AB* and *floating head*^*n1/n1*^ (*flh/noto*) mutants^37^. Sample sizes and developmental stages are stated below. Randomization was not performed as experimental groups were determined by genotype. Blinding was performed for the generation and analysis of the single-cell data.

### CRISPR–Cas9-based mutagenesis of F_0_ embryos

To generate somatic gene deletions in early zebrafish embryos, we used CRISPR–Cas9 with two or three sgRNAs as described previously^78^. In brief, sgRNAs were designed using CHOPCHOP (http://chopchop.cbu.uib.no/) to target 5′ exons and the functional domain of each selected TF and synthesized (IDT) (Supplementary Fig. 20b). sgRNA sequences are listed in Supplementary Table 6. Duplex sgRNA was prepared by mixing equimolar amounts of Alt-R crRNA and Alt-tracrRNA (IDT) in IDT Duplex Buffer, heating to 95 °C and slowly cooling to room temperature (RT) for 20 min. For the final mix of ribonucleoprotein complex (RNPs), around 4 μM duplex sgRNA was assembled with around 5 μM CRISPR–Cas nuclease (Alt-R S.p. HiFi Cas9 Nuclease V3) in 3 M KCl 0.025% and phenol red solution. The activity of HiFi Cas9 and selected sgRNAs was confirmed with regular PCR, Sanger sequencing and capillary electrophoresis, as described previously^40^. In brief, DNA from eight embryos for each combination of Cas9 and sgRNAs was extracted at 10 hpf. PCR amplification was performed with primers complementary to sequences 250 bp upstream and downstream of the PAM sequences (Supplementary Table 6). In addition, tracking of indels by decomposition (TIDE)^79^ analysis was used to predict the percentage of indels at the target locus (Supplementary Fig. 20c). *flh*^*n1/n1*^ mutant embryos were generated by crossing heterozygotes and selecting mutants on the basis of their morphology at 10 hpf.

### Embryo collection and processing

Zebrafish embryos were produced by natural matings and injected at the one-cell stage with around 2–4 nl of RNP solution into the blastodisc. Embryos were incubated at 28 °C after removing those damaged during the injection process. After 9 hpf, embryos were enzymatically dechorionated and deyolked as previously described^32^. In brief, embryos were dechorionated by incubation in 1 mg ml^−1^ pronase, washed with ‘blue water’ and then transferred into plastic Petri dishes coated with 2% agarose with methylene blue water. Deyolking was performed manually by ‘squeezing’ the yolk out of the blastoderm cap with a closed pair of forceps inserted between the embryonic blastoderm and the yolk. The layer of cells detached from the yolk was transferred to a 1.5-ml Eppendorf tube with 50 μl of DMEM/F12 medium. For each experiment, 40–50 individual CRISPR–Cas9-targeted embryos (crispants) were prepared for dissociation into single-cell suspensions. Cell dissociation was performed according to the previous report (Farrell et al.)^32^. DMEM/F12 medium was added to the Eppendorf tube to bring the total volume to 200 μl. Cells were mechanically dissociated by flicking the tube 15 times and pipetting 3 times. The cell mixture was spun at 300*g* for 2 min and twice washed with PBS + 0.1% BSA. The same procedure was followed to collect and dissociate cells from WT and *flh*^*n1/n1*^ mutant embryos.

### RNA extraction and qRT-PCR

Total RNA was extracted from approximately 50 embryos for each experimental condition, homogenized in Trizol (Life Technologies) and further purified following Qiagen RNEasy Mini Kit instructions^80^. One microgram of total RNA was used to synthesize cDNA with the iScript kit (BioRad) following the manufacturer’s protocol. SYBR green (BioRad) qRT-PCR reactions were run in a CFX Connect Real-Time PCR detection system (BioRad) with three technical replicates. The primers used are listed in Supplementary Table 6.

### Whole-mount in situ hybridization

An antisense RNA probe for *nog1* was generated from plasmid pBSKII^81^, previously linearized with Not1, and used as a template for in vitro transcription using NEB T7 RNA polymerase and RNTPs labelled with digoxygenin (DIG) (Roche). WISH was performed according to a previous report^82^. In brief, embryos were fixed overnight in 4% paraformaldehyde (PFA) in phosphate-buffered saline (PBS), rinsed in PBS + 0.1% Tween 20 (PBT) and dehydrated in methanol. Embryos were then rehydrated in PBT and incubated for at least 2 h in hybridization solution (HM) with 50% formamide (in 0.75 M sodium chloride, 75 mM sodium citrate, 0.1% Tween 20, 50 μg ml^−1^ heparin (Sigma) and 200 μg ml^−1^ tRNA) at 70 °C, then hybridized overnight at 70 °C with antisense probes diluted approximately 1 ng μl^−1^ in hybridization solution. Embryos were washed through a series of 10 min, 70 °C washes in HM diluted with 2× SSC buffer (0.3 M sodium chloride and 30 mM sodium citrate) once in each of the following: 75% HM, 50% HM, 25% HM and 100% 2× SSC. The same gradual change from SSC to PBT was performed for the subsequent washes. Embryos were blocked at RT for several hours in PBT with 2% goat serum and 2 mg ml^−1^ bovine serum albumin (BSA), then incubated overnight at 4 °C with anti-DIG antibody (Roche 11093274910) at 1:5,000 on a horizontal shaker (40 rpm). Embryos were rinsed six times for 15 min per wash in PBT, and then in staining buffer (PBT+100 mM Tris pH 9.5, 50 mM MgCl_2_ and 100 mM NaCl) before staining with BM Purple solution (Roche).

### HCR

HCR was performed according to the protocols provided by Molecular Instruments (https://www.molecularinstruments.com). Embryos were fixed at 10 hpf with 4% PFA, dehydrated with methanol and rehydrated as described for WISH above. Embryos were pre-hybridized in hybridization buffer (Molecular Instruments) for 1 h at 37 °C and subsequently incubated in 200 μl of hybridization solution containing 1 pg of probes overnight at 37 °C. Embryos were then washed four times in wash buffer (Molecular Instruments) followed by two washes in 5× SSCT, containing 5× SSC buffer (Thermo Fisher Scientific) and 0.1% Tween 20 (Sigma). For the pre-amplification step, embryos were incubated in amplification buffer (Molecular Instruments) for more than 1 h. At the same time, hairpin mixtures were prepared by heating 12 pmol of hairpin 1 (H1) and 2 (H2) for each sample to 95 °C for 90 s, followed by cooling in the dark for 30 min at RT. H1 and H2 were mixed and then added to 200 μl amplification buffer. Embryos were incubated in the hairpin mixture at RT overnight in the dark. On the third day, embryos were washed more than 4 times in 5× SSCT and either stored at 4 °C or mounted for microscopy.

### Microscopy

Embryos subjected to HCR were mounted in 3% low-melt agarose in glass-bottomed 35-mm Petri dishes. Alternatively, embryos were manually deyolked and flattened on a glass slide with one to two drops of 3% methylcellulose. Images of the anterior and posterior body regions were taken by acquiring around 200-μm *z*-stacks with a 1-μm step, using a 10× objective lens on a modified Olympus IX81 inverted spinning disc confocal microscope equipped with Voltran and Cobolt steady-state lasers and a Hamamatsu ImagEM EM CCD digital camera.

### Image quantification with IMARIS software

Individual confocal 3D datasets were analysed with IMARIS 9.9 software (Bitplane). On the basis of the DAPI signal, radii were determined by taking half of the longest diameter of each nucleus, which was measured as a single spot using the ‘spots’ view in IMARIS. These parameters were applied in all images used for quantification. Nuclei positive for specific probes within a selected area were identified using the ‘spots’ view as spots with a signal in the specific channel that overlapped with DAPI spots. Analysis was performed on eight embryos: four anterior and four posterior per experimental group.

### 10X Chromium procedure

For single-cell library preparation on the 10X Genomics platform, we used: the Chromium Single Cell 3′ Library & Gel Bead Kit v2 (PN-120237), Chromium Single Cell 3′ Chip kit v2 (PN-120236) and Chromium i7 Multiplex Kit (PN-120262), according to the manufacturer’s instructions in the Chromium Single Cell 3′ Reagents Kits V2 User Guide. Before cell capture, methanol-fixed cells were placed on ice, then spun at 3,000 rpm for 5 min at 4 °C, followed by resuspension and rehydration in PBS, as described previously^83^. A total of 17,000 cells were loaded per lane of the chip, aiming to capture 10,000 single-cell transcriptomes. The resulting cDNA libraries were quantified on an Agilent TapeStation and sequenced on an Illumina NextSeq 550.

### 10X Chromium scRNA-seq data processing

#### 10X alignment and digital GEM generation

The Cell Ranger v5.0.1 pipeline (https://support.10xgenomics.com/single-cell-gene-expression/software/downloads/latest) was used to process data generated using the 10X Chromium platform. Cell Ranger processes, filters and aligns reads generated with the Chromium single-cell RNA sequencing platform. Following this step, the default Cell Ranger pipeline was implemented, and the filtered output data were used for downstream analyses.

### Zebrafish scRNA-seq data processing

We used the R package Seurat (v.4.0.1) to process scRNA-seq data. Cells were filtered by RNA count and percentage of mitochondrial genes to remove low-quality cells. Data were normalized using the Seurat NormalizeData() function. Variable genes were identified using the FindVariableFeatures() function with nfeature = 2,000. Data were integrated by applying Seurat functions, SelectIntegrationFeatures(), FindIntegrationAnchors() and IntegrateData() using default parameters. After data scaling, PCA and clustering were performed. The data after cell calling may include cells with very low mRNA counts generated from non-cell GEMs and ambient RNA. To remove such non-cell GEM data, we assessed the RNA count distribution to remove clusters with an abnormal RNA count distribution. Scaling, PCA, clustering and *t-*SNE were performed again after removing the cells above. *t-*SNE was calculated using the first 30 principal components. We applied the same pipeline to the WT reference, *flh* mutant and crispant scRNA-seq data.

After data integration and standard scRNA-seq preprocessing, the whole WT reference scRNA-seq data were annotated as follows. The segmentation labels generated in the Farrell et al.^32^ zebrafish scRNA-seq data were transferred to the new scRNA-seq data using the Seurat function, FindTransferAnchors and TransferData, with default parameters. We manually adjusted the cell annotation to account for differences in the timing of cell collection. We generated cell-type annotations for the clustering data generated in the previous step by referring to the Farrell et al. dataset annotation labels. The WT reference cell-type annotations were transferred to the other scRNA-seq data using the same Seurat label transfer functions.

To compare cell identity on the same 2D embedding space, we used UMAP and the UMAP transfer function. We first calculated UMAP with axial mesoderm clusters in WT reference datasets. Using this pre-trained UMAP model, we projected KO and control axial mesoderm data onto the same UMAP 2D embedding space constructed with WT reference data.

Cell density was visualized using a KDE plot. First, we performed random downsampling to adjust the cell number between the LOF control samples. (i) Whole-cell populations were randomly subsampled into a subset to have an equal cell number to the smaller dataset. (ii) Then, axial mesoderm cells were selected and subjected to KDE calculation. KDE was calculated with the scipy.stat.gaussian_kde function. The calculated KDE was visualized with the matplotlib.pyplot.contour function. We used the same contour threshold levels between the LOF and control samples.

In addition to the UMAP transfer analysis above, the WT data, *lhx1a* crispant and *tyr* crispant data were analysed with UMAP without data transfer (Supplementary Fig. 21). The 10 hpf axial mesoderm cell data were integrated using Seurat functions (SelectIntegrationFeatures(), FindIntegrationAnchors(), and IntegrateData() with default parameters), and then UMAP graph and Louvain cluster were calculated (RunPCA(), FindNeighbors(reduction = "pca", dims = 1:30), RunUMAP(reduction = "pca", dims = 1:30, min.dist = 1), FindClusters(resolution = 1.5)).

### NMF

We performed NMF with our *lhx1a* crispants scRNA-seq dataset according to a previous report^32^. The normalized UMI counts were standardized, log-transformed and subjected to NMF calculation with sklearn.decomposition.NMF(n_components=40). Each module was manually annotated by its cluster enrichment and gene ontology calculated with the top 30 genes with high module weight. Gene annotation, weight and ontology are provided in Supplementary Table 3. Gene ontology was calculated with the g:Profiler API (https://biit.cs.ut.ee/gprofiler/page/apis). The background was set to all genes used in the NMF calculation. Clusters governed by a single gene were excluded from our analysis.

### Statistical testing

Details of all statistical tests performed are provided in Supplementary Table 4. Scipy stat module (scipy version 1.7.0) was used for statistical analysis. In summary, we selected the statistical method as follows: (i) chi-square test was used to analyse the relationships of categorical variables; (ii) Wilcoxon rank-sum test (Mann–Whitney *U* test) was used when the data distribution type was not apparent; (iii) in cases in which the data distribution followed a Gaussian distribution, a *t*-test was used. Where multiple comparisons were made, the Bonferroni correction was applied. An alternative hypothesis (one-tailed or two-tailed) was selected depending on the aim of the analysis.

### Reporting summary

Further information on research design is available in the Nature Portfolio Reporting Summary linked to this article.

## Online content

Any methods, additional references, Nature Portfolio reporting summaries, source data, extended data, supplementary information, acknowledgements, peer review information; details of author contributions and competing interests; and statements of data and code availability are available at 10.1038/s41586-022-05688-9.

## Supplementary information

Supplementary Information This file contains Supplementary Figures 1–26, Supplementary Tables 1–6 and Supplementary References

Reporting Summary

Supplementary Table 1 Details of CellOracle pre-built base GRN data. This table details the different species for which we have constructed base GRNs.

Supplementary Table 2 TF annotation and previous study references. This table details the previously reported knockout phenotypes in mouse haematopoiesis and zebrafish development.

Supplementary Table 3 Negative PS sum scores for zebrafish KO simulation. This table details the ranked list of predicted regulators of somitogenesis, with published phenotypes.

Supplementary Table 4 Details of statistical tests. This table details the statistical tests used throughout this study.

Supplementary Table 5 *Lhx1a* crispant NMF data. This table details the NMF analysis of *Lhx1a* crispants. For statistical tests, Wilcoxon rank-sum test, Two-tailed was used. The p-value was corrected with the Bonferroni correction method.

Supplementary Table 6 sgRNA and primer sequences. This table details the sgRNA and primer sequences used in the zebrafish experiments.

Supplementary Video 1 **Visualization of the differentiation vector field and TF KO vector fields**. The same 2D vector field used in Fig. 1c–f is visualized as a movie. The left panel shows the differentiation vector field of Paul et al. haematopoiesis data. The centre and right panels show CellOracle KO simulation vectors for *Spi1* and *Gata1* transcription factors, respectively. The particle visual effects were generated with Unity (version 2020.3.0f1) and Visual Effect Graph Asset: https://docs.unity3d.com/Packages/com.unity.visualeffectgraph@13.1/manual/VisualEffectGraphAsset.html

## Data Availability

All data, including sequencing reads and single-cell expression matrices, are available from the GEO under accession codes GSE72859 (ref. ^16^), GSE112824 (ref. ^32^) and GSE145298 for the zebrafish profiling from this study; and from ArrayExpress under accession codes E-MTAB-7325 (*Tal1*^−/−^ chimeras) and E-MTAB-7324 (wild-type chimeras). Simulations can be explored at https://celloracle.org.

## Code availability

CellOracle code, documentation and tutorials are available at GitHub: https://github.com/morris-lab/CellOracle.
