# Supplementary material for: Dissecting cell identity via network inference and in silico gene perturbation
Source: Nature. 2023 Feb 8;614(7949):742–51. doi: 10.1038/s41586-022-05688-9 (PMC9946838; doi:10.1038/s41586-022-05688-9)
Supplement: Supplementary file 1 — This file contains Supplementary Figures 1–26, Supplementary Tables 1–6 and Supplementary References [file 41586_2022_5688_MOESM1_ESM.pdf]

nature portfolio

https://doi.org/10.1038/s41586-022-05688-9

Supplementary information

Dissecting cell identity via network inference and in silico gene perturbation

In the format provided by the authors and unedited

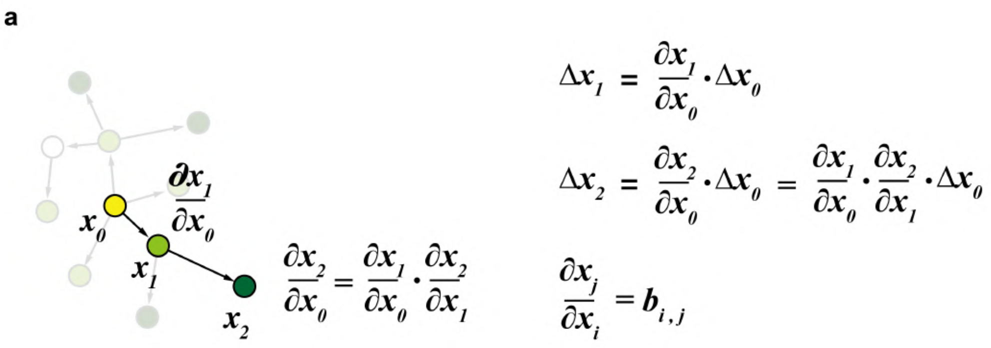

Supplementary Figure 1. Partial derivative and chain rule used in signal propagation. (a) Illustration of CellOracle signal propagation. x_i represents the gene expression value of gene i in the cell of interest. Here, x_0 regulates x_1, and x_1 regulates x_2. The signal propagation uses the gradient between two variables x_i and x_j. If gene i and j have a direct connection, the partial derivative $\frac{\partial x_j}{\partial x_i}$ is already obtained as a constant coefficient value $b_{i,j}$ in the linear regression model. If gene i and gene j have an indirect connection, $\frac{\partial x_j}{\partial x_i}$ is calculated using the chain rule, as illustrated by $\frac{\partial x_2}{\partial x_0}$.

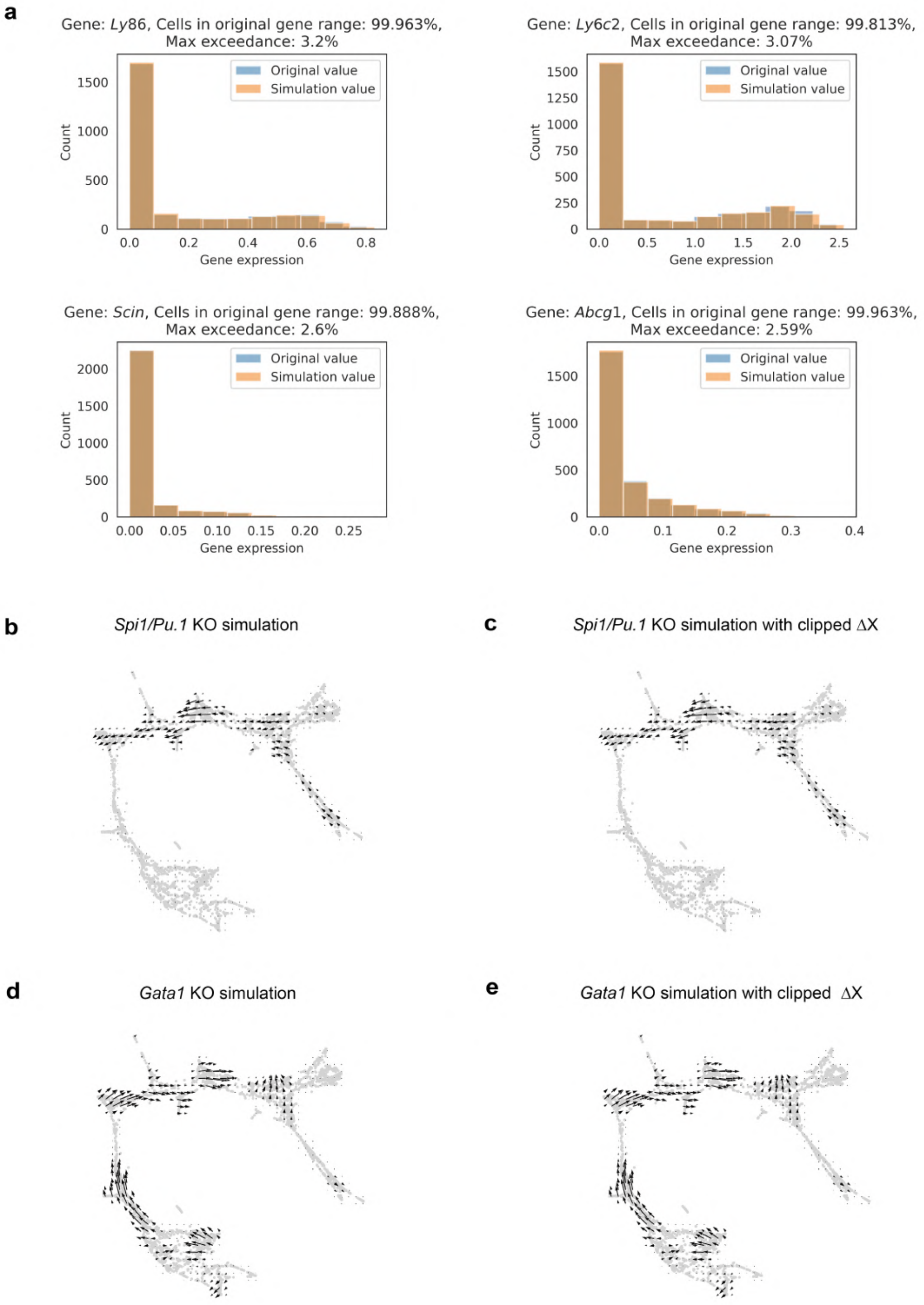

Supplementary Figure 2. Evaluation of simulation value distribution range.

(a) Histogram of the original wild-type (WT) gene expression value (blue, 50% transparent) and simulation gene expression level (orange, 50% transparent). The simulation gene expression level is the sum of the simulated gene expression shift and original WT gene expression value ($X_{\text{simulation gene expression level}} = X_{\text{original}} + \Delta X_{\text{simulated,}}$). The max exceedance value denotes the percentage difference of the maximum value of the simulated gene expression level compared to the maximum value of the wild-type gene expression level. For the *Spi1/Pu.1* KO simulation in the Paul et al.,

hematopoiesis analysis, the top 4 genes with the highest max exceedance value are shown. Almost all data points of *Ly86, Ly6c2, Scin*, and *Abcg1* distributions exist inside the WT data distribution (99.963%, 99.813%, 99.888%, 99.963%, respectively). The difference in data distribution between the out-of-distribution and WT range is minimal (3.2%, 3.07%, 2.60%, and 2.59%, respectively). **(b-e)** Comparison of the CellOracle KO simulation vector field between default settings (**b, d**) and clipped ΔX, in which out-of-distribution values are clipped into the WT distribution range (**c, e**). (**b, c**) *Spi1/Pu.1* KO simulation vector field. (**b, e**) *Gata1* KO simulation vector field. All parameters, except for the ΔX data range clipping, are the same between (**b**) and (**c**), (**d**) and (**e**).

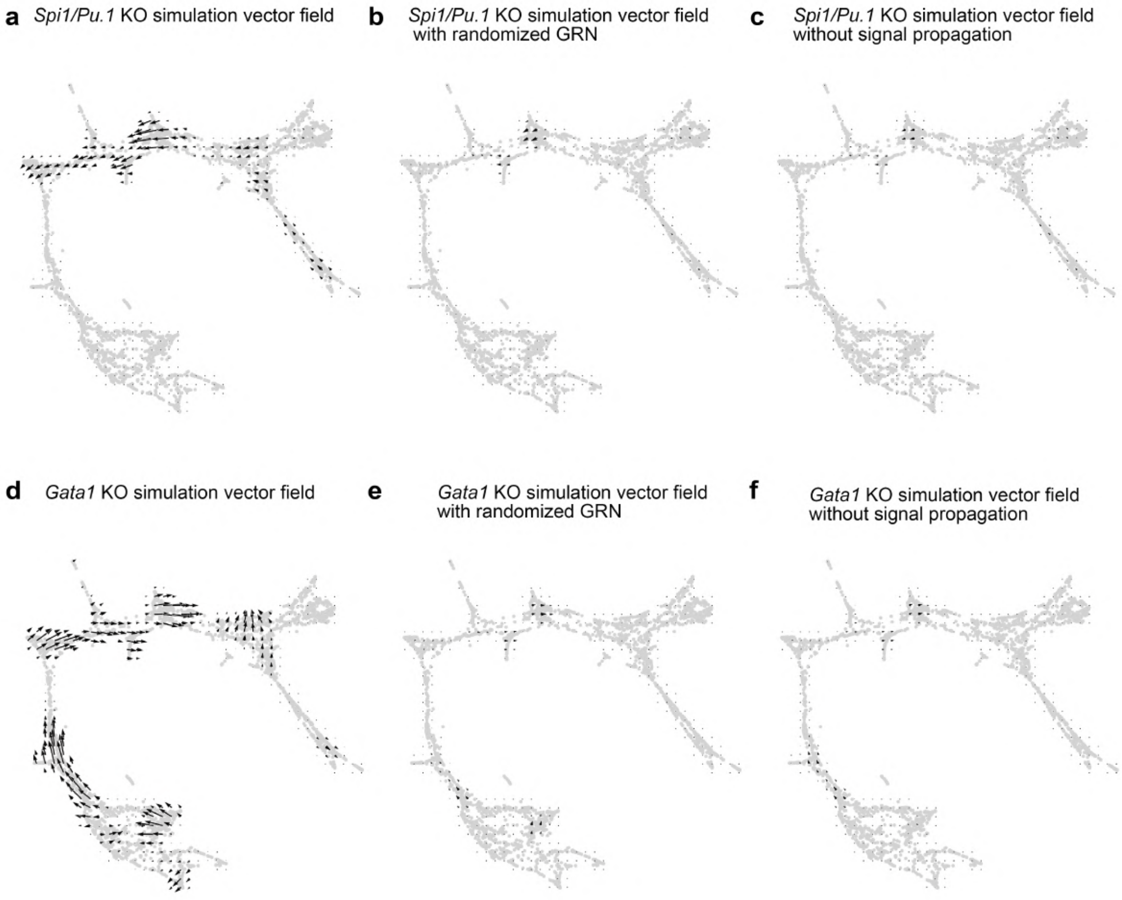

Supplementary Figure 3. CellOracle simulation results generated with randomized GRN or no signal propagation. **(a-c)** *Spi1/Pu.1* KO simulation and **(d-f)** *Gata1* KO simulation using the Paul hematopoiesis dataset. **(b, e)** CellOracle KO simulations performed with randomized GRNs. **(c, f)** CellOracle KO simulations performed without signal propagation. In this latter case, the cell identity shift vector was calculated based on input TF expression loss alone, thus representing information from only a singular TF's expression pattern.

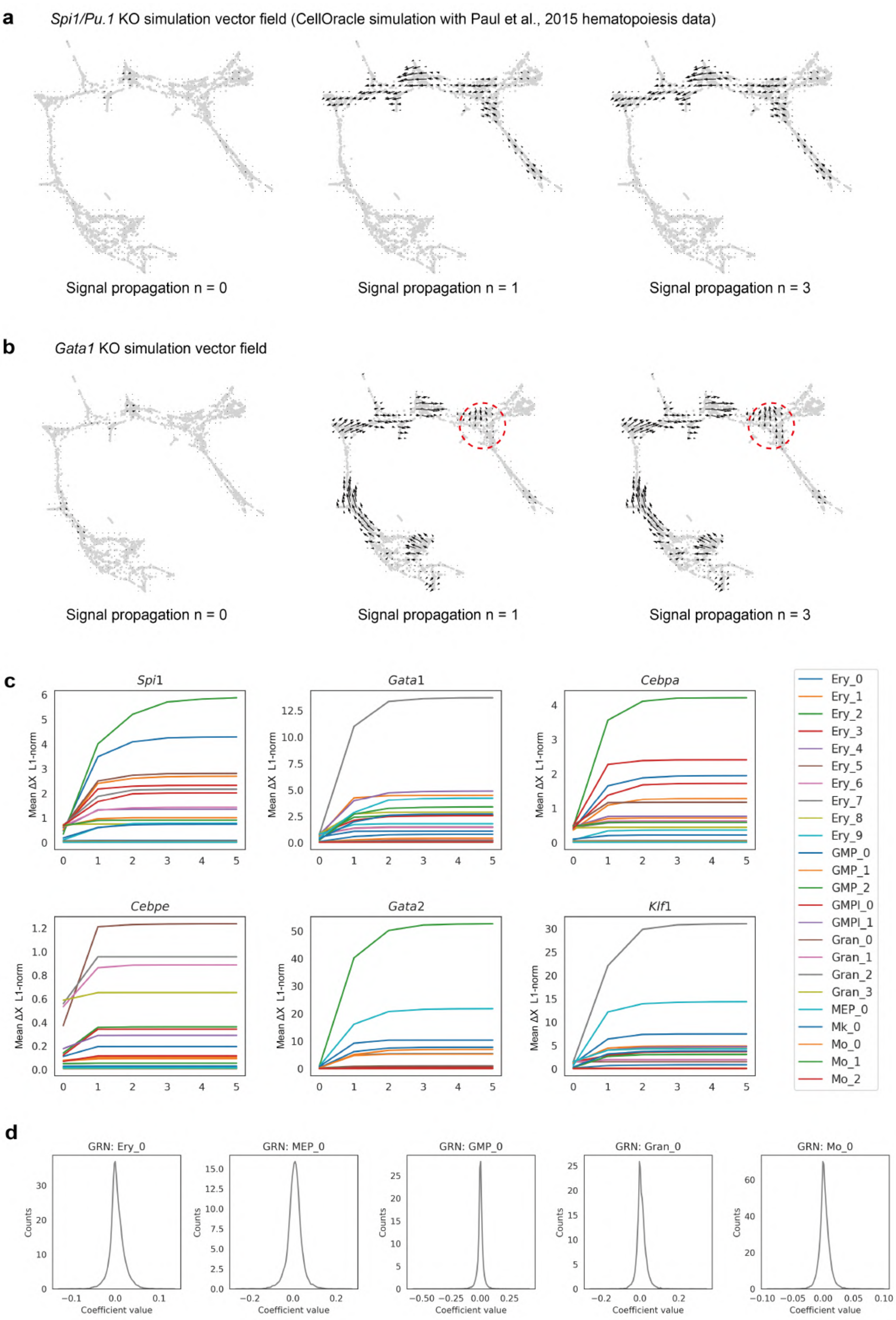

Supplementary Figure 4. Evaluation of signal propagation number with Paul et al. (2015) hematopoiesis data. (a) *Spi1/Pu.1* KO and **(b)** *Gata1* KO simulation vector fields with different

numbers of signal propagation rounds (n=0, 1, and 3, respectively). Vectors in late GMP/early granulocyte clusters for the *Gata1* KO simulations are circled in red. **(c)** L1-norm of ΔX, representing the summed magnitudes of all components in the simulated gene expression shift vectors. Following TF KO simulation of *Spi1/Pu.1, Gata1, Cebpα, Cebpε, Gata2*, and *Klf1*, the L1-norm of ΔX is calculated under various signal propagation numbers (n=0, 1, 2, 3, 4, and 5). The L1-norm of ΔX is calculated for each vector, and the value is grouped by clusters and visualized as a mean value. **(d)** Distribution of the GRN coefficient values visualized by a kernel density distribution plot.

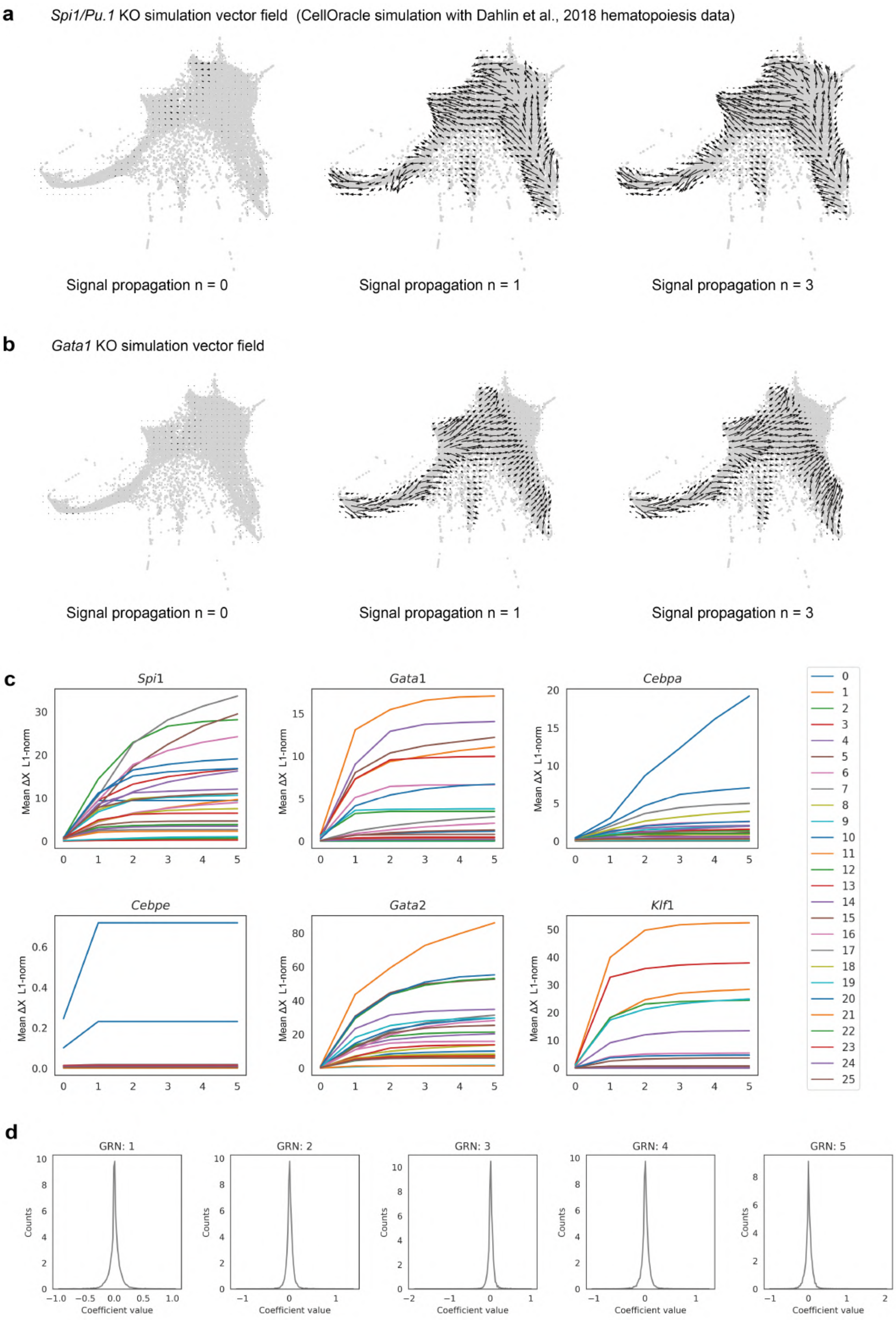

Supplementary Figure 5. Evaluation of signal propagation number with Dahlin et al. (2018) hematopoiesis data. (a) *Spi1/Pu.1* KO and **(b)** *Gata1* KO simulation vector fields with different

numbers of signal propagation rounds (n=0, 1, and 3, respectively). **(c)** L1-norm of ΔX, representing the summed magnitudes of all components in the simulated gene expression shift vectors. Following TF KO simulation of *Spi1/Pu.1, Gata1, Cebp*α*, Cebp*ε*, Gata2*, and *Klf1*, the L1-norm of ΔX is calculated under various signal propagation numbers (n=0, 1, 2, 3, 4, and 5). The L1-norm of ΔX is calculated for each vector, and the value is grouped by clusters and visualized as a mean value. **(d)** Distribution of the GRN coefficient value visualized by a kernel density distribution plot.

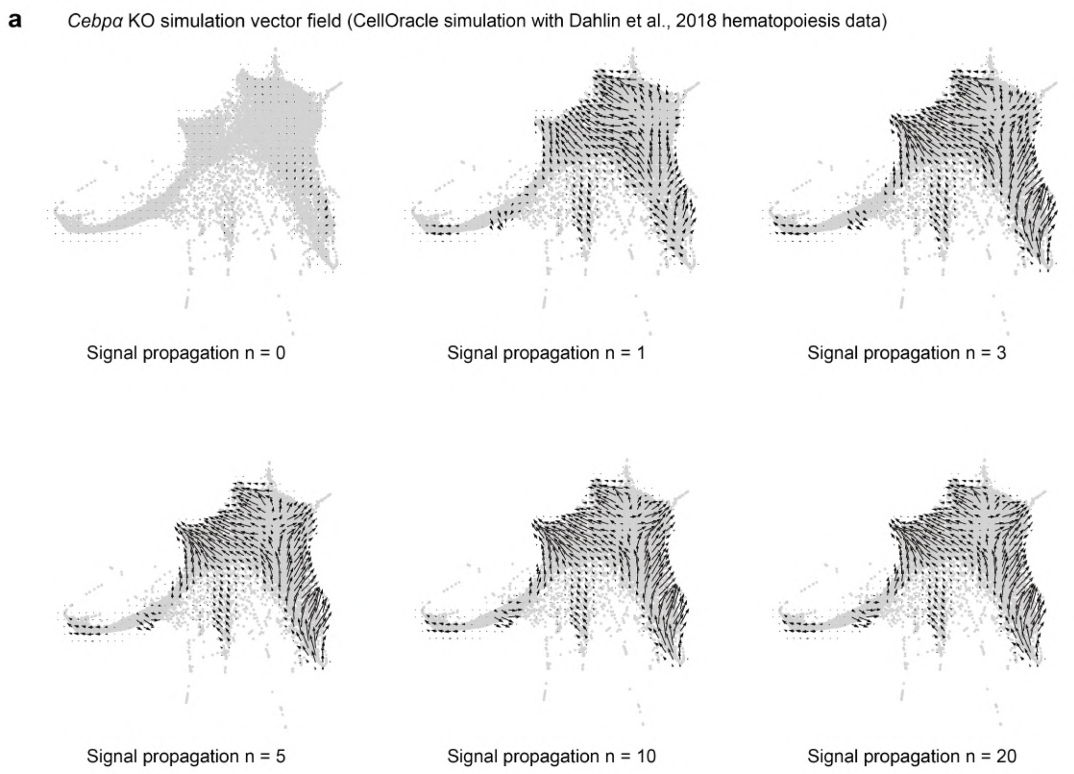

Supplementary Figure 6. *Cebpα* KO simulation performed with various signal propagation numbers using Dahlin et al. (2018) hematopoiesis data. (a) *Cebpα* KO simulation vector field with different signal propagation number (n=0, 1, 3, 5, 10, and 20).

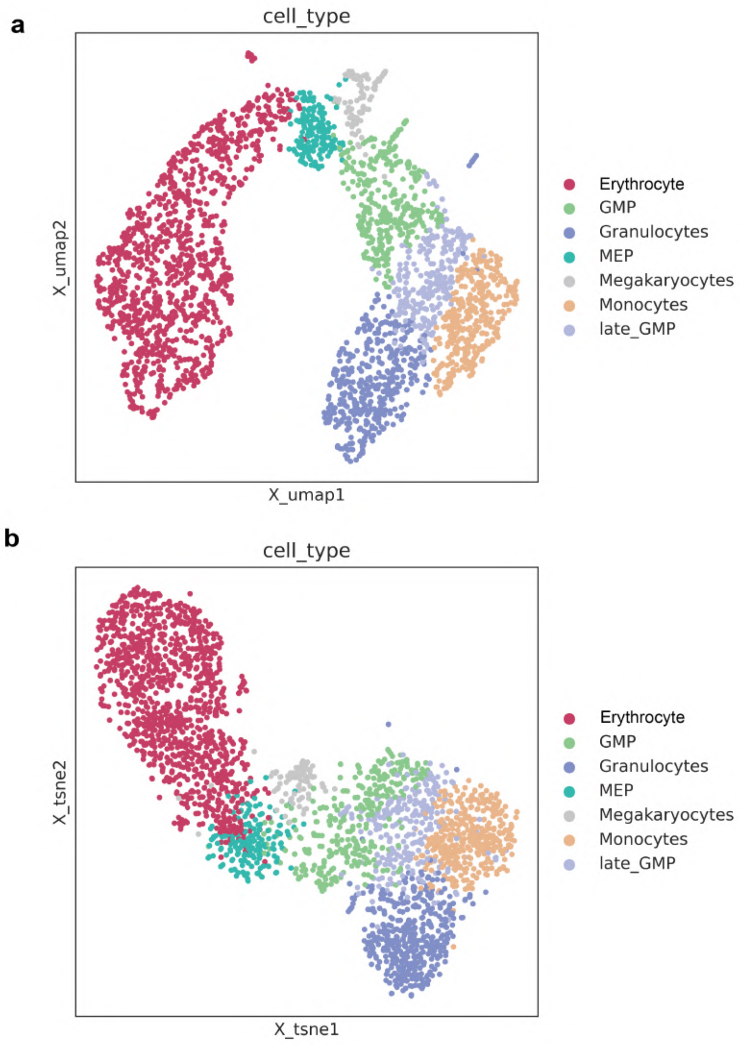

Supplementary Figure 7. Paul et al. hematopoiesis scRNA-seq data UMAP and t-SNE plots. (a) UMAP plot of 2,730 myeloid progenitor cells from Paul et al. (2015)[1] with cell-type annotation. **(b)** t-SNE graph of 2,730 myeloid progenitor cells from Paul et al. (2015)[1] with cell-type annotation.

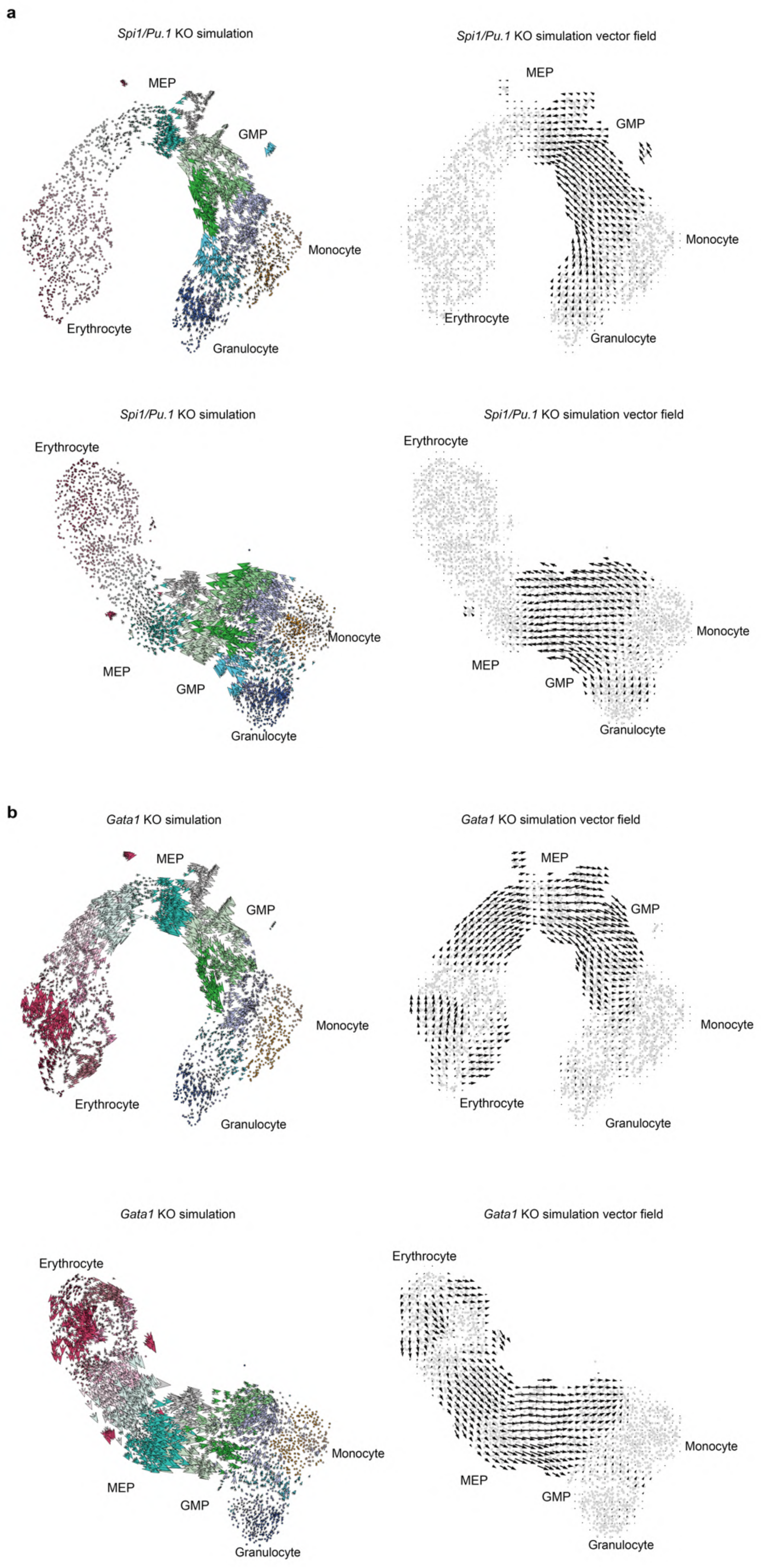
a
Spi1/Pu.1 KO simulation
Spi1/Pu.1 KO simulation vector field
MEP
GMP
Monocyte
Erythrocyte
Granulocyte
Spi1/Pu.1 KO simulation
Spi1/Pu.1 KO simulation vector field
Erythrocyte
Monocyte
MEP
GMP
Granulocyte
b
Gata1 KO simulation
Gata1 KO simulation vector field
MEP
GMP
Monocyte
Erythrocyte
Granulocyte
Gata1 KO simulation
Gata1 KO simulation vector field
Erythrocyte
Monocyte
MEP
GMP
Granulocyte

c

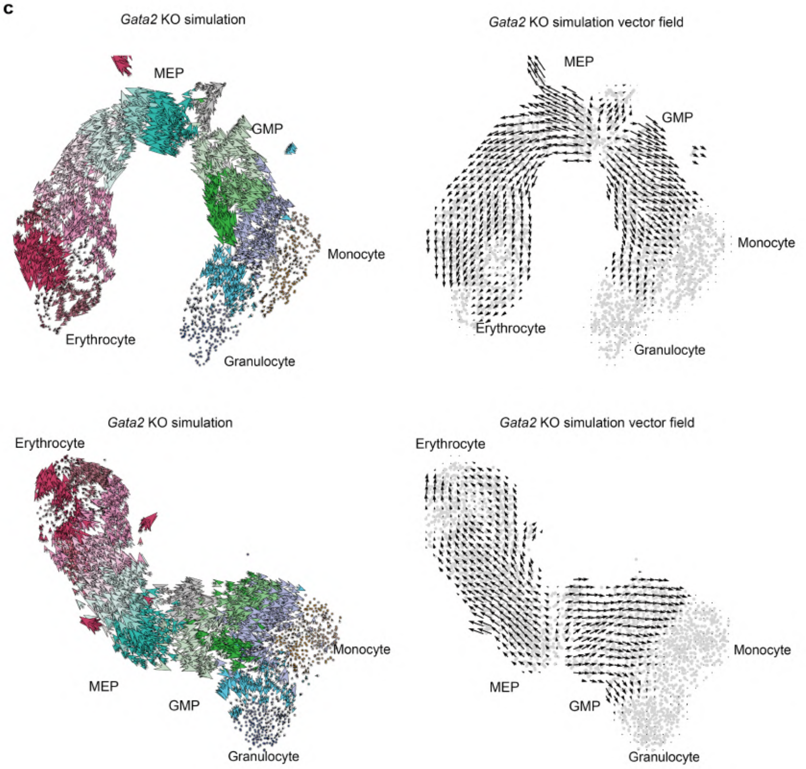
Gata2 KO simulation
Gata2 KO simulation vector field
MEP
GMP
Monocyte
Erythrocyte
Granulocyte
Gata2 KO simulation
Gata2 KO simulation vector field
Erythrocyte
Monocyte
MEP
GMP
Granulocyte

d

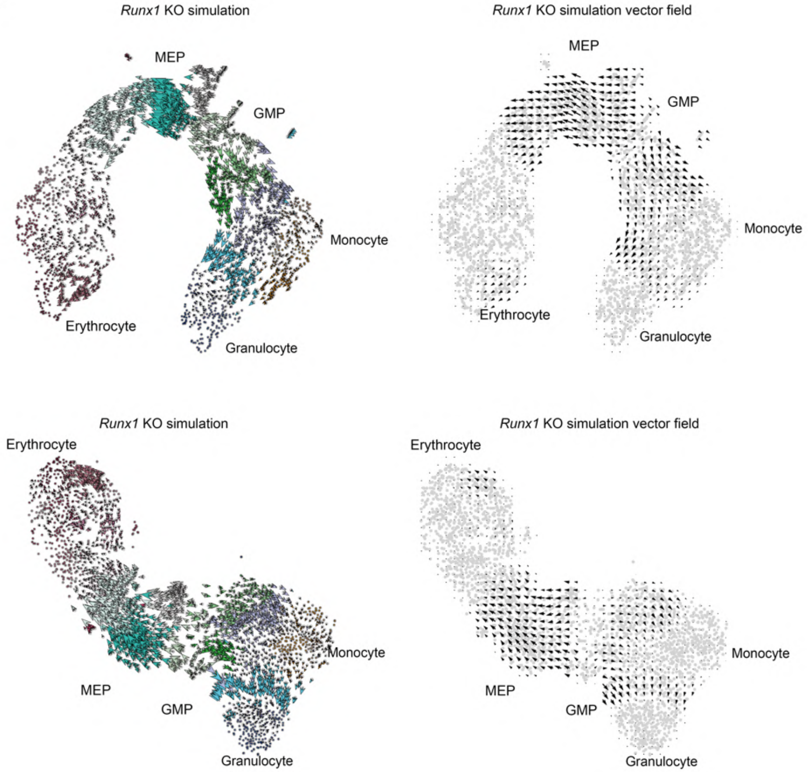
Runx1 KO simulation
Runx1 KO simulation vector field
MEP
GMP
Monocyte
Erythrocyte
Granulocyte
Runx1 KO simulation
Runx1 KO simulation vector field
Erythrocyte
Monocyte
MEP
GMP
Granulocyte

e

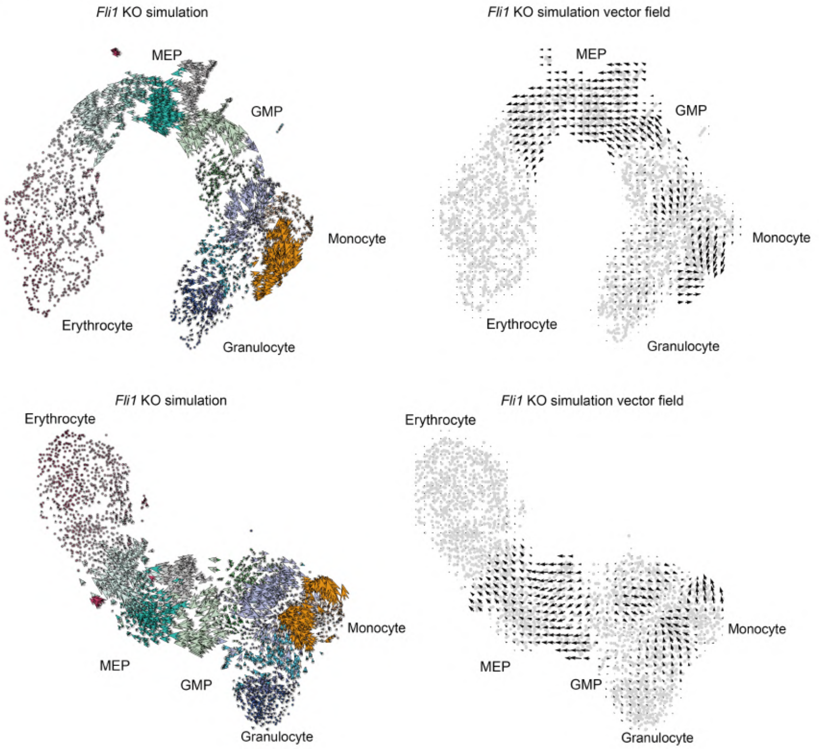
Fli1 KO simulation
MEP
GMP
Monocyte
Erythrocyte
Granulocyte
Fli1 KO simulation vector field
MEP
GMP
Monocyte
Erythrocyte
Granulocyte
Fli1 KO simulation
Erythrocyte
Monocyte
MEP
GMP
Granulocyte
Fli1 KO simulation vector field
Erythrocyte
Monocyte
MEP
GMP
Granulocyte

f

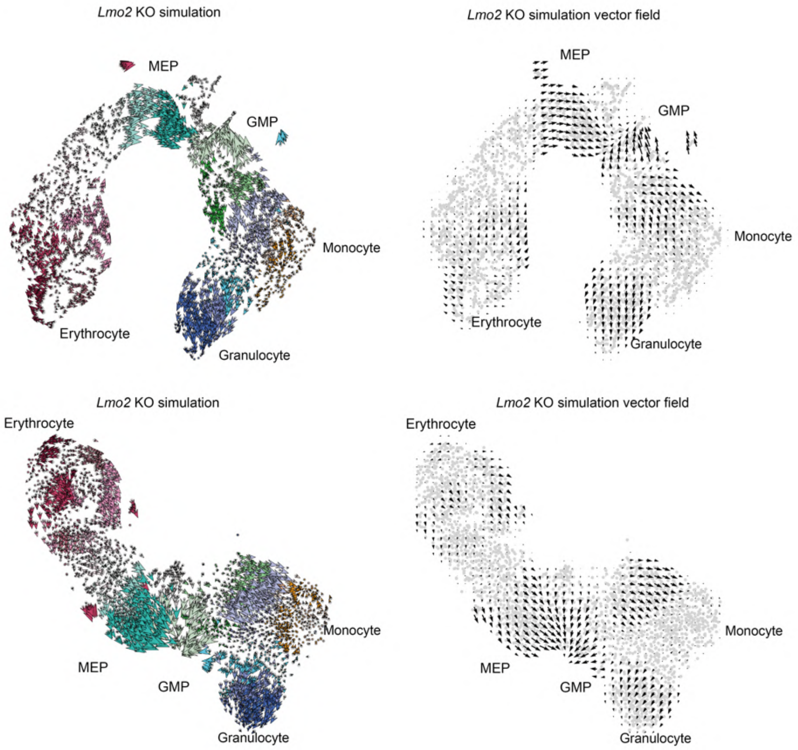
Lmo2 KO simulation
MEP
GMP
Monocyte
Erythrocyte
Granulocyte
Lmo2 KO simulation vector field
MEP
GMP
Monocyte
Erythrocyte
Granulocyte
Lmo2 KO simulation
Erythrocyte
Monocyte
MEP
GMP
Granulocyte
Lmo2 KO simulation vector field
Erythrocyte
Monocyte
MEP
GMP
Granulocyte

g

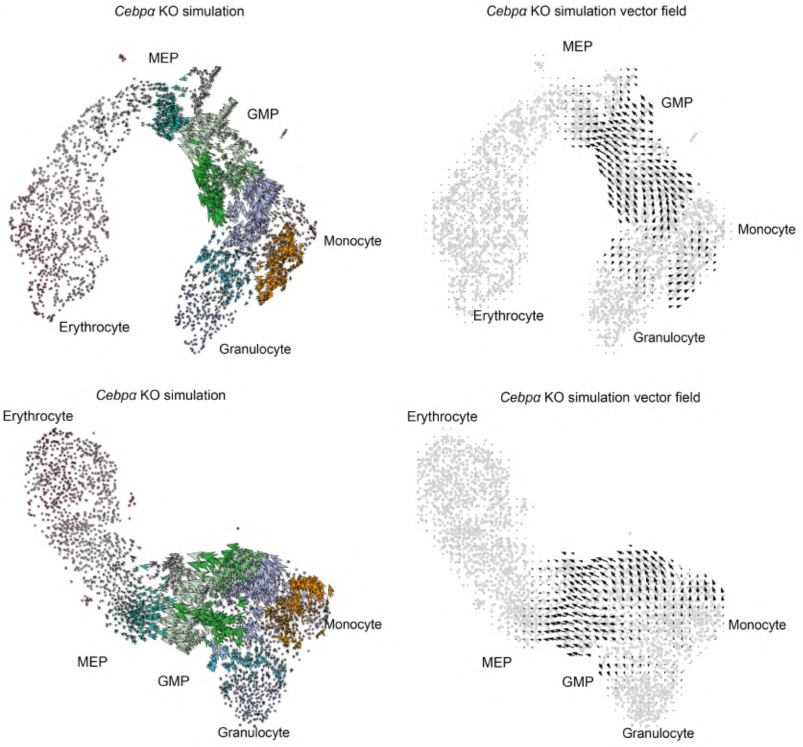

Cebpα KO simulation
MEP
GMP
Monocyte
Erythrocyte
Granulocyte
Cebpα KO simulation vector field
MEP
GMP
Monocyte
Erythrocyte
Granulocyte
Cebpα KO simulation
Erythrocyte
Monocyte
MEP
GMP
Granulocyte
Cebpα KO simulation vector field
Erythrocyte
Monocyte
MEP
GMP
Granulocyte

h

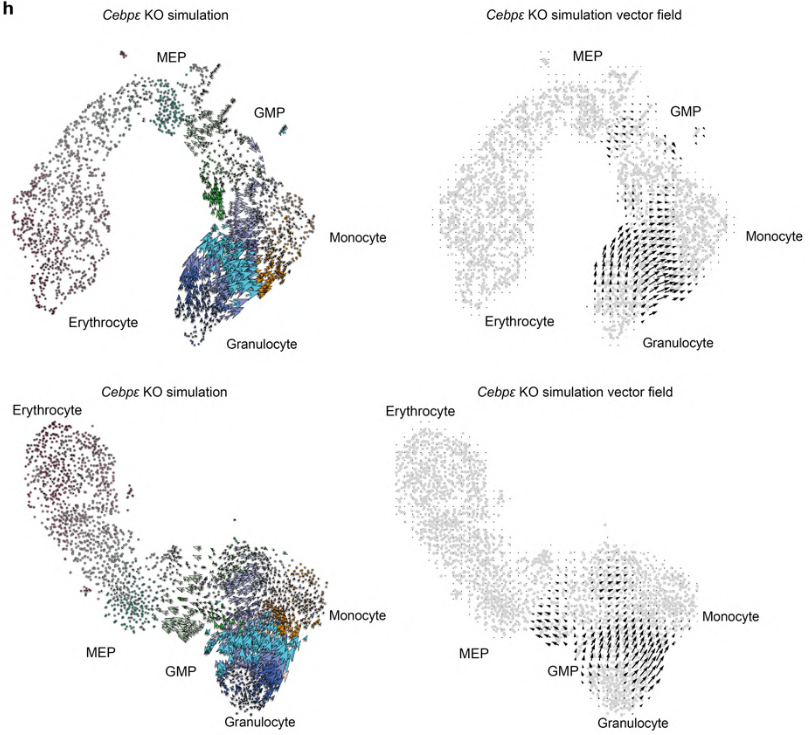

Cebpε KO simulation
MEP
GMP
Monocyte
Erythrocyte
Granulocyte
Cebpε KO simulation vector field
MEP
GMP
Monocyte
Erythrocyte
Granulocyte
Cebpε KO simulation
Erythrocyte
Monocyte
MEP
GMP
Granulocyte
Cebpε KO simulation vector field
Erythrocyte
Monocyte
MEP
GMP
Granulocyte

i

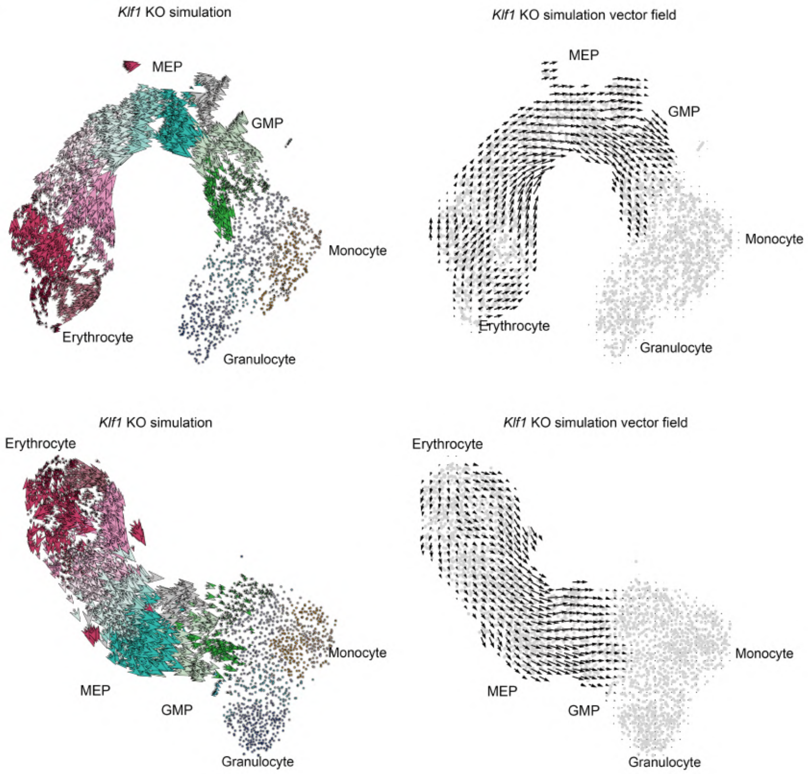
Klf1 KO simulation
MEP
GMP
Monocyte
Erythrocyte
Granulocyte
Klf1 KO simulation vector field
MEP
GMP
Monocyte
Erythrocyte
Granulocyte
Klf1 KO simulation
Erythrocyte
Monocyte
MEP
GMP
Granulocyte
Klf1 KO simulation vector field
Erythrocyte
Monocyte
MEP
GMP
Granulocyte

j

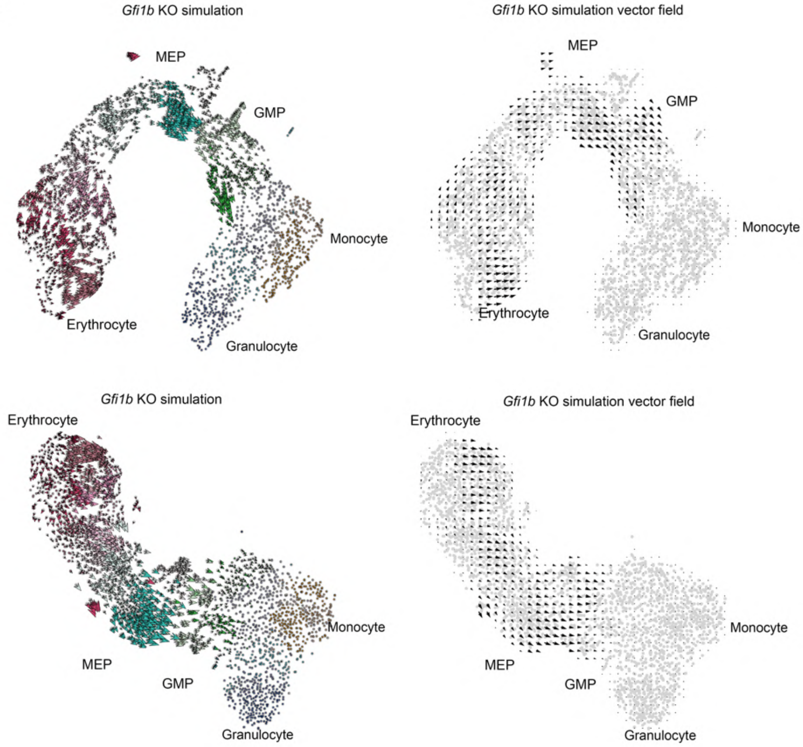
Gfi1b KO simulation
MEP
GMP
Monocyte
Erythrocyte
Granulocyte
Gfi1b KO simulation vector field
MEP
GMP
Monocyte
Erythrocyte
Granulocyte
Gfi1b KO simulation
Erythrocyte
Monocyte
MEP
GMP
Granulocyte
Gfi1b KO simulation vector field
Erythrocyte
Monocyte
MEP
GMP
Granulocyte

k

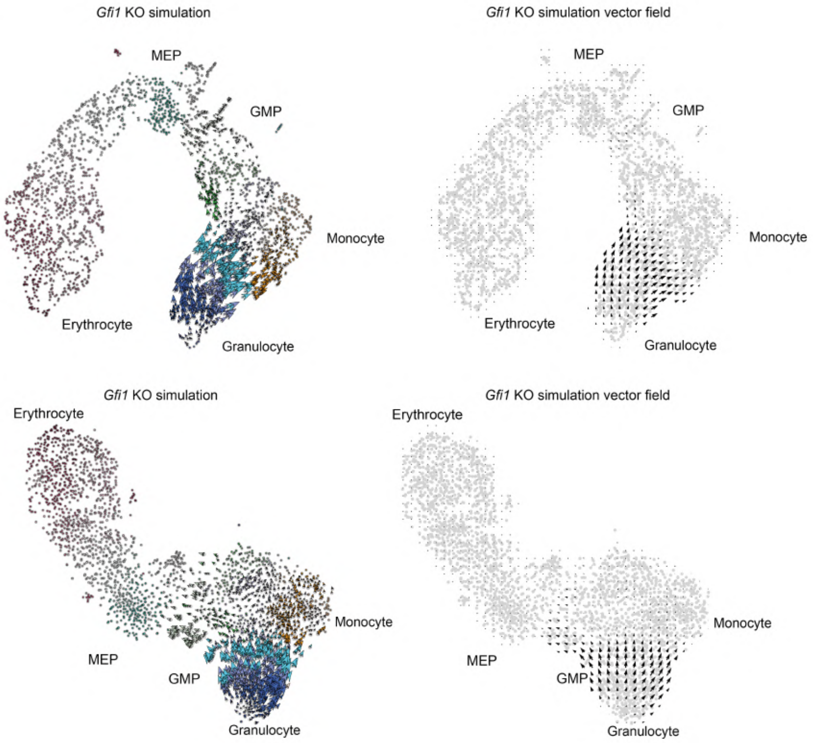

l

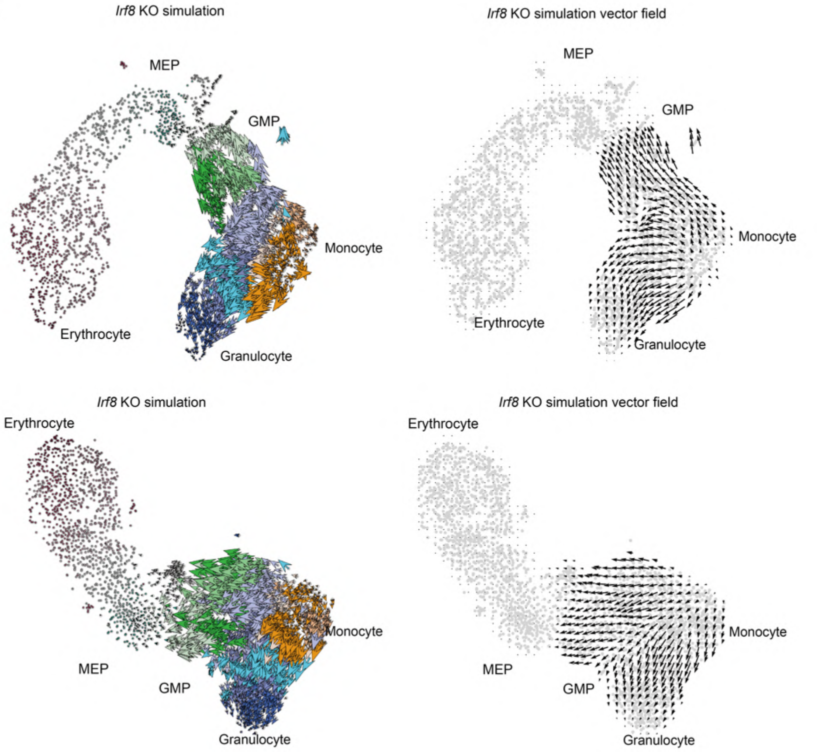

Supplementary Figure 8. CellOracle simulation vector visualized on UMAP or t-SNE plots. (a-l) CellOracle gene perturbation simulation is performed using the UMAP or t-SNE plot. The same parameters as **Fig. 1 and Fig. 2** are used except for the dimensionality reduction. CellOracle KO

simulation results are shown as single-cell resolution vector (left panels) and vector field map (right panels). UMAP graph or t-SNE graph is used for the simulation embedding (upper panels and lower panels, respectively).

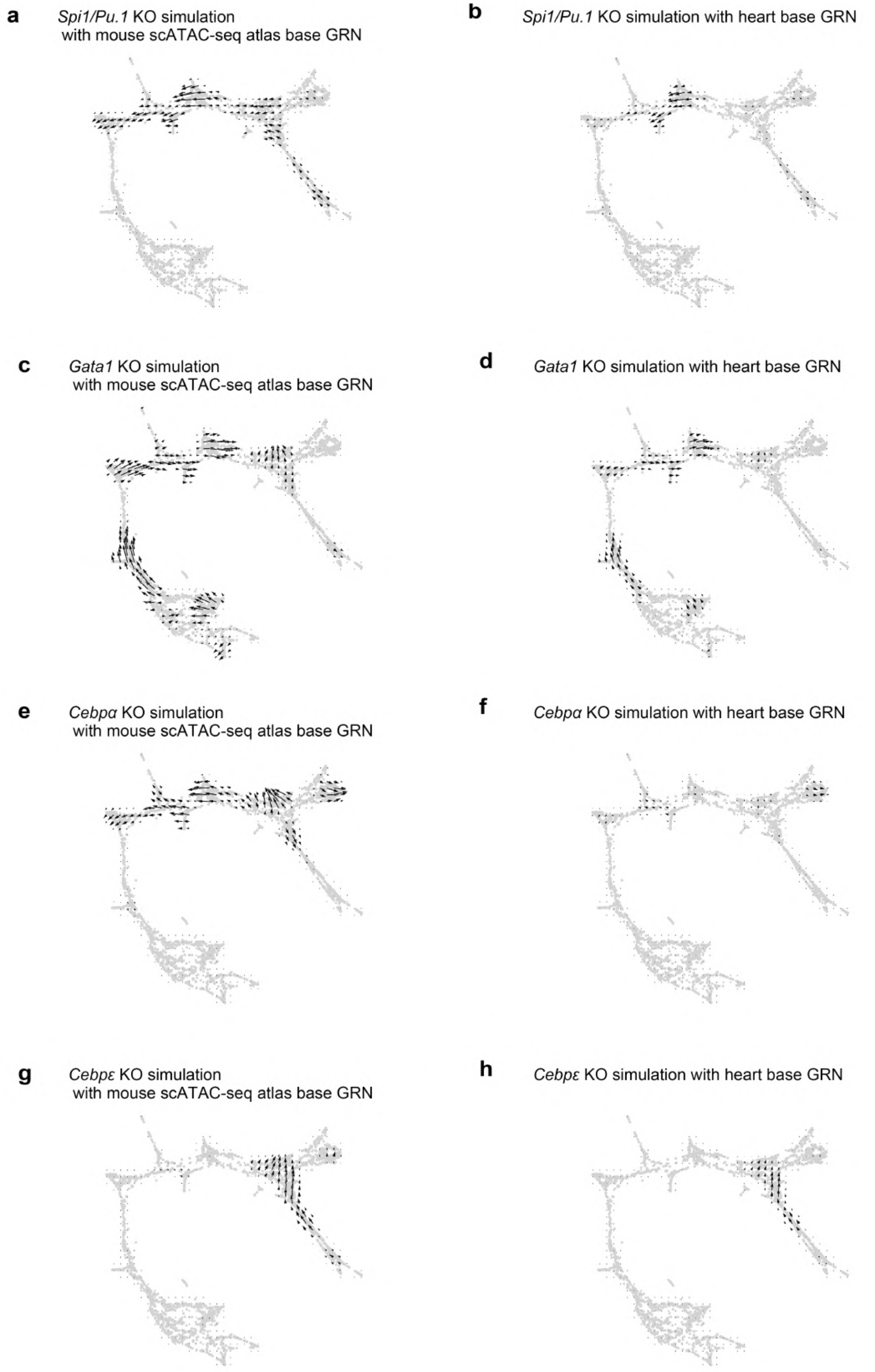

Supplementary Figure 9. CellOracle KO simulation with unrelated cell type base GRN.
(a, c, e, g) TF KO simulation with Paul et al. hematopoiesis scRNA-seq data and Cusanovich et al.[2] mouse scATAC-seq atlas base GRN. This is the same simulation condition shown in **Figs. 1**

and 2. (b, d, f, h) TF KO simulation with heart ATAC-seq base GRN. **(a, b)** *Spi1/Pu.1* KO simulation vector field. **(c, d)** *Gata1* KO simulation vector field. **(e, f)** *Cebp*α KO simulation vector field. **(g, h)** *Cebp*ε KO simulation vector field.

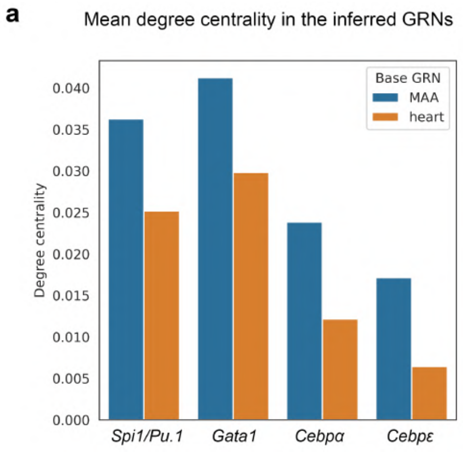

Supplementary Figure 10. Degree centrality in CellOracle GRNs generated with unrelated cell type base GRN. (a) Comparison of degree centrality scores between GRN models constructed with different base GRNs. Context-dependent GRN model initiated with the Cusanovich et al. mouse scATAC-seq atlas (MAA) base GRN (blue) and heart ATAC-seq base GRN (orange). Degree centrality represents the normalized number of directly connected edges in the network. The four TFs shown in **Supplementary Data Fig 9** are analyzed: *Spi1/Pu.1 Gata1*, *Cebpα,* and *Cebpε*. Degree centrality scores for each cluster-specific GRN model are averaged and shown as a bar plot.

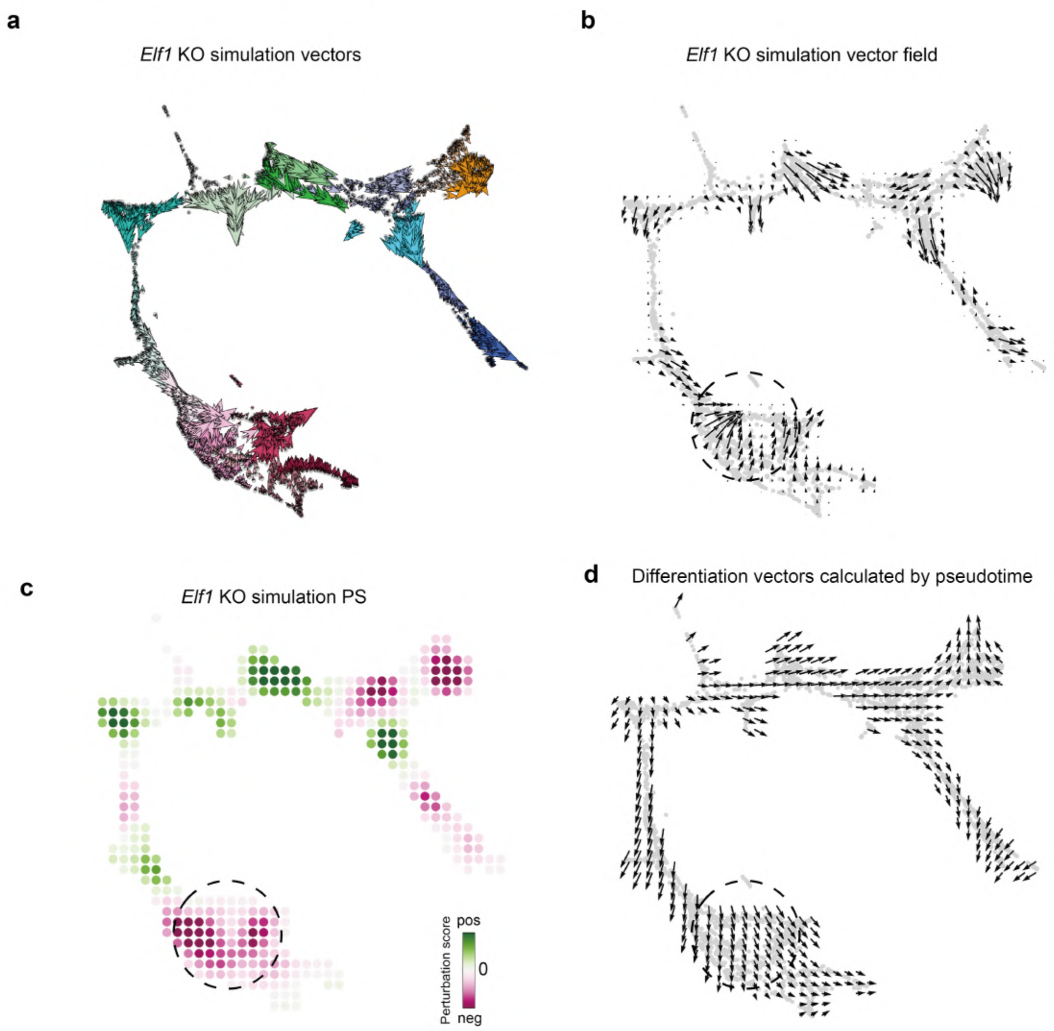

Supplementary Figure 11. CellOracle *Elf1* KO simulation results using Paul et al. (2015) hematopoiesis data. (a, b) CellOracle KO simulation for *Elf1*. The cell state transition vectors are visualized at single-cell resolution **(a)** or as a vector field **(b)**. **(c)** Perturbation scores (PS) of *Elf1* KO simulation (negative score: magenta; positive score: green). **(d)** Differentiation vector field. This is the same data as **Fig. 1c**. In late erythrocytes, highlighted by the dashed line, the differentiation vector points from upper left to lower right, while the *Elf1* KO vector points from lower left to upper right, producing the negative PS.

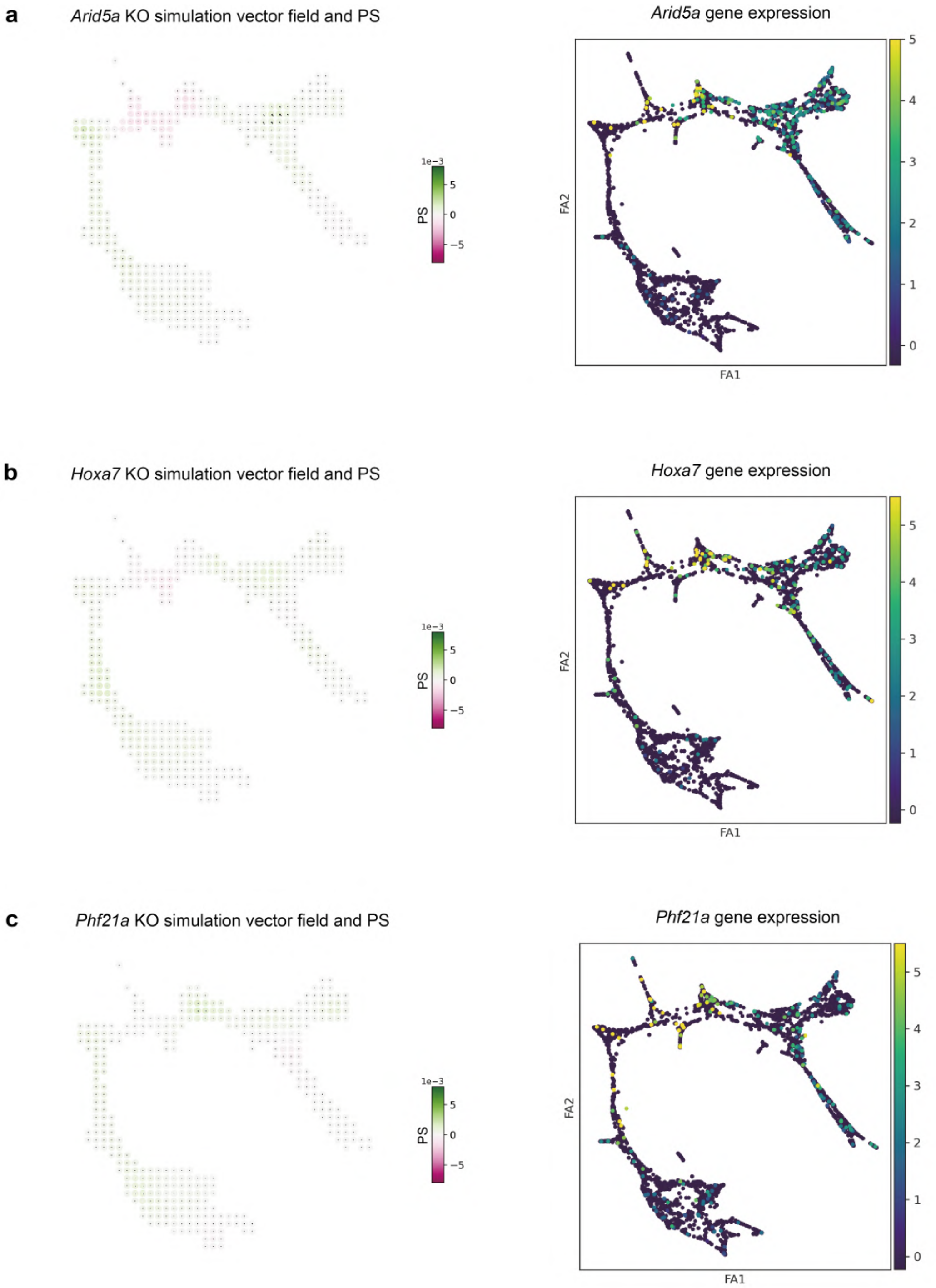

Supplementary Figure 12. CellOracle simulation results for TFs that show no predicted phenotype. (a-c) CellOracle KO simulation cell state transition-vector field (left panel) and gene expression (log-transformed UMIs, right panel) for highly expressed TFs with little to no change in phenotypes expected based on the shift vectors.

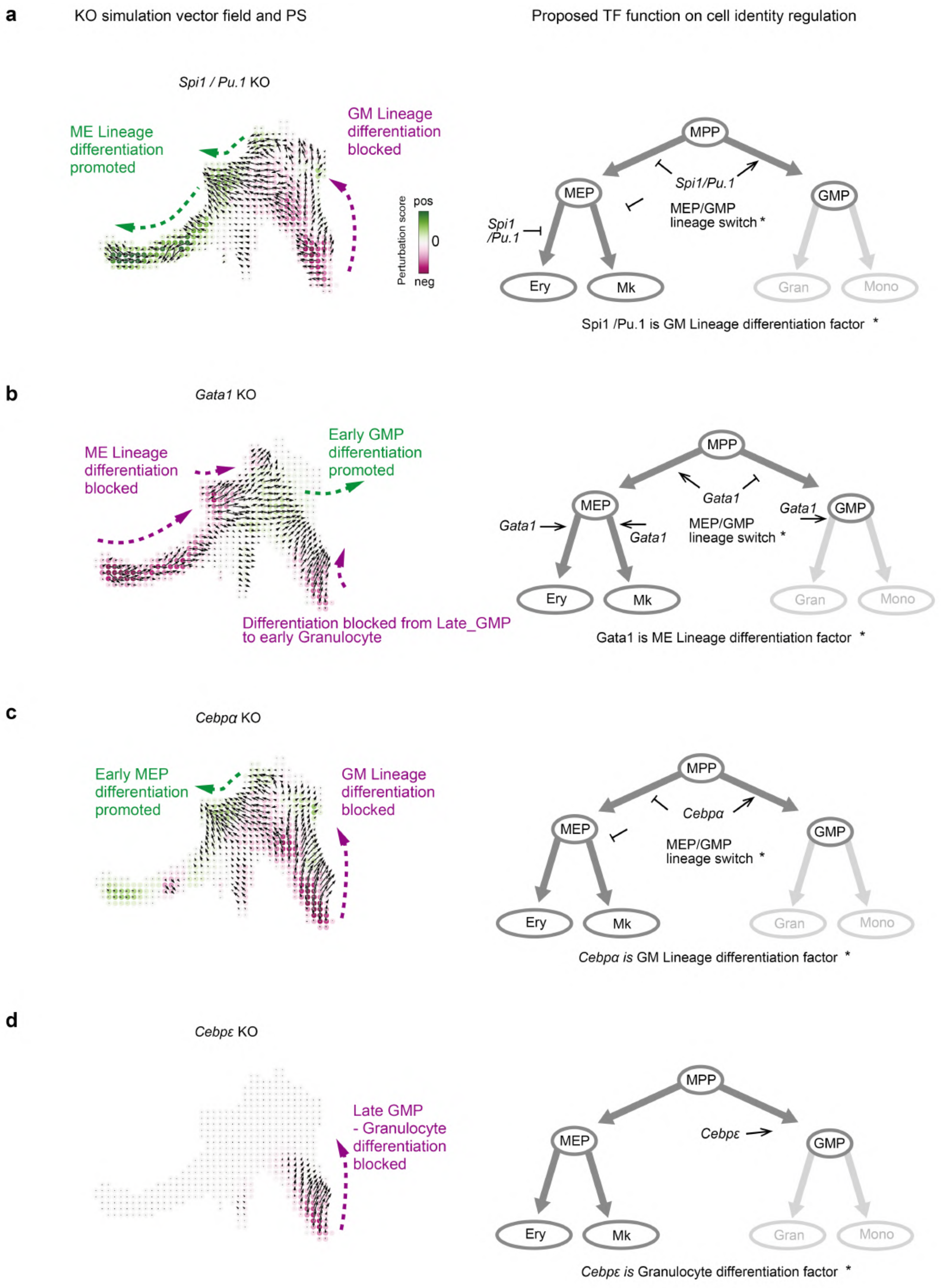
a
KO simulation vector field and PS
Proposed TF function on cell identity regulation
Spi1 / Pu.1 KO
ME Lineage differentiation promoted
GM Lineage differentiation blocked
Perturbation score
pos
0
neg
MPP
MEP
GMP
Spi1/Pu.1
MEP/GMP lineage switch *
Spi1 /Pu.1
Ery
Mk
Gran
Mono
Spi1 /Pu.1 is GM Lineage differentiation factor *
b
Gata1 KO
ME Lineage differentiation blocked
Early GMP differentiation promoted
Differentiation blocked from Late_GMP to early Granulocyte
MPP
Gata1
MEP
Gata1
Gata1
MEP/GMP lineage switch *
Gata1
GMP
Ery
Mk
Gran
Mono
Gata1 is ME Lineage differentiation factor *
c
Cebpα KO
Early MEP differentiation promoted
GM Lineage differentiation blocked
MPP
Cebpα
MEP
GMP
MEP/GMP lineage switch *
Ery
Mk
Gran
Mono
Cebpα is GM Lineage differentiation factor *
d
Cebpε KO
Late GMP - Granulocyte differentiation blocked
MPP
MEP
Cebpε
GMP
Ery
Mk
Gran
Mono
Cebpε is Granulocyte differentiation factor *

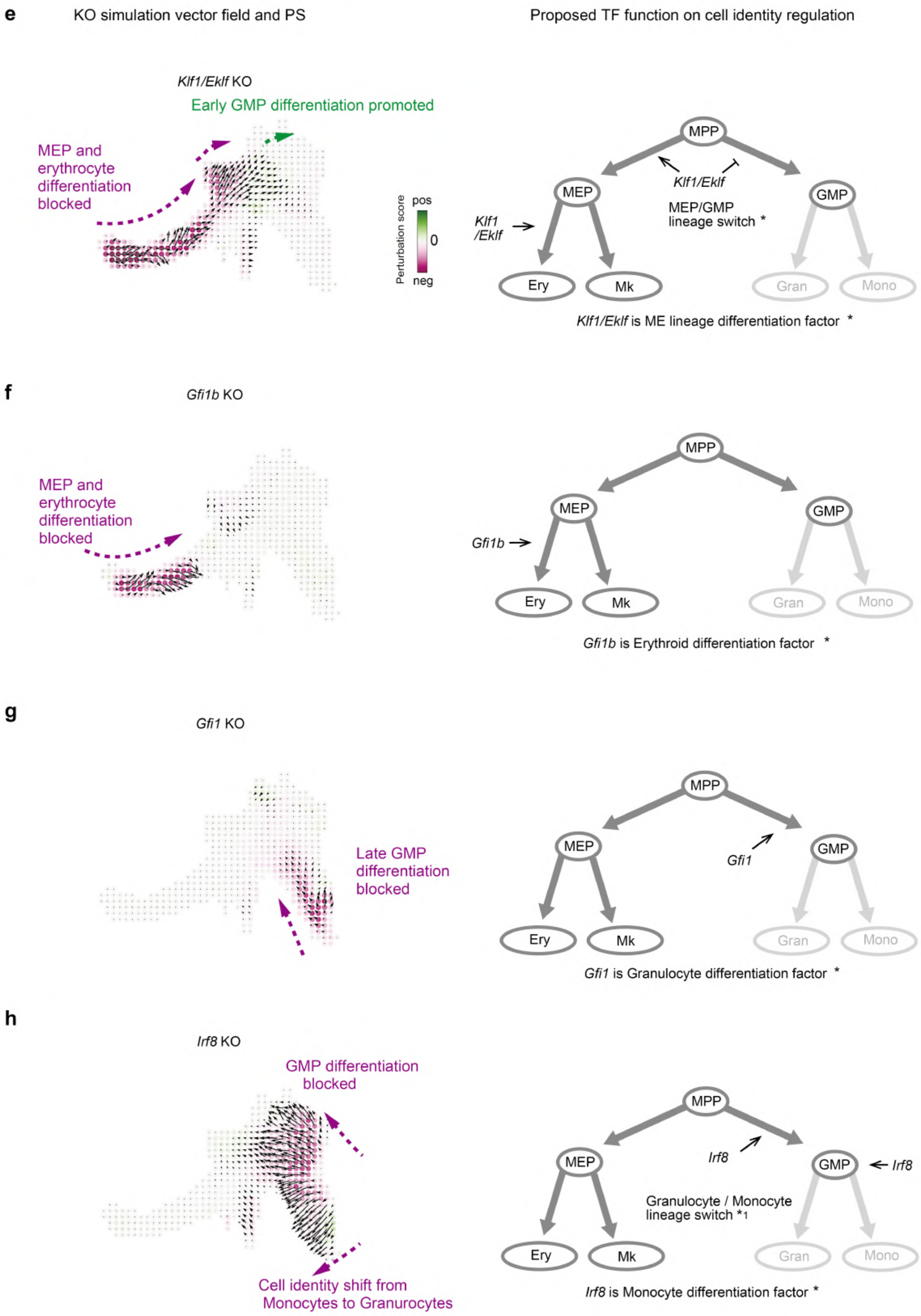
e
KO simulation vector field and PS
Proposed TF function on cell identity regulation
Klf1/Eklf KO
Early GMP differentiation promoted
MEP and erythrocyte differentiation blocked
Perturbation score
pos
0
neg
MPP
MEP
GMP
Klf1/Eklf
MEP/GMP lineage switch *
Klf1 /Eklf
Ery
Mk
Gran
Mono
Klf1/Eklf is ME lineage differentiation factor *
f
Gfi1b KO
MEP and erythrocyte differentiation blocked
MPP
MEP
GMP
Gfi1b
Ery
Mk
Gran
Mono
Gfi1b is Erythroid differentiation factor *
g
Gfi1 KO
Late GMP differentiation blocked
MPP
MEP
GMP
Gfi1
Ery
Mk
Gran
Mono
Gfi1 is Granulocyte differentiation factor *
h
Irf8 KO
GMP differentiation blocked
Cell identity shift from Monocytes to Granurocytes
MPP
MEP
GMP
Irf8
Irf8
Granulocyte / Monocyte lineage switch *1
Ery
Mk
Gran
Mono
Irf8 is Monocyte differentiation factor *

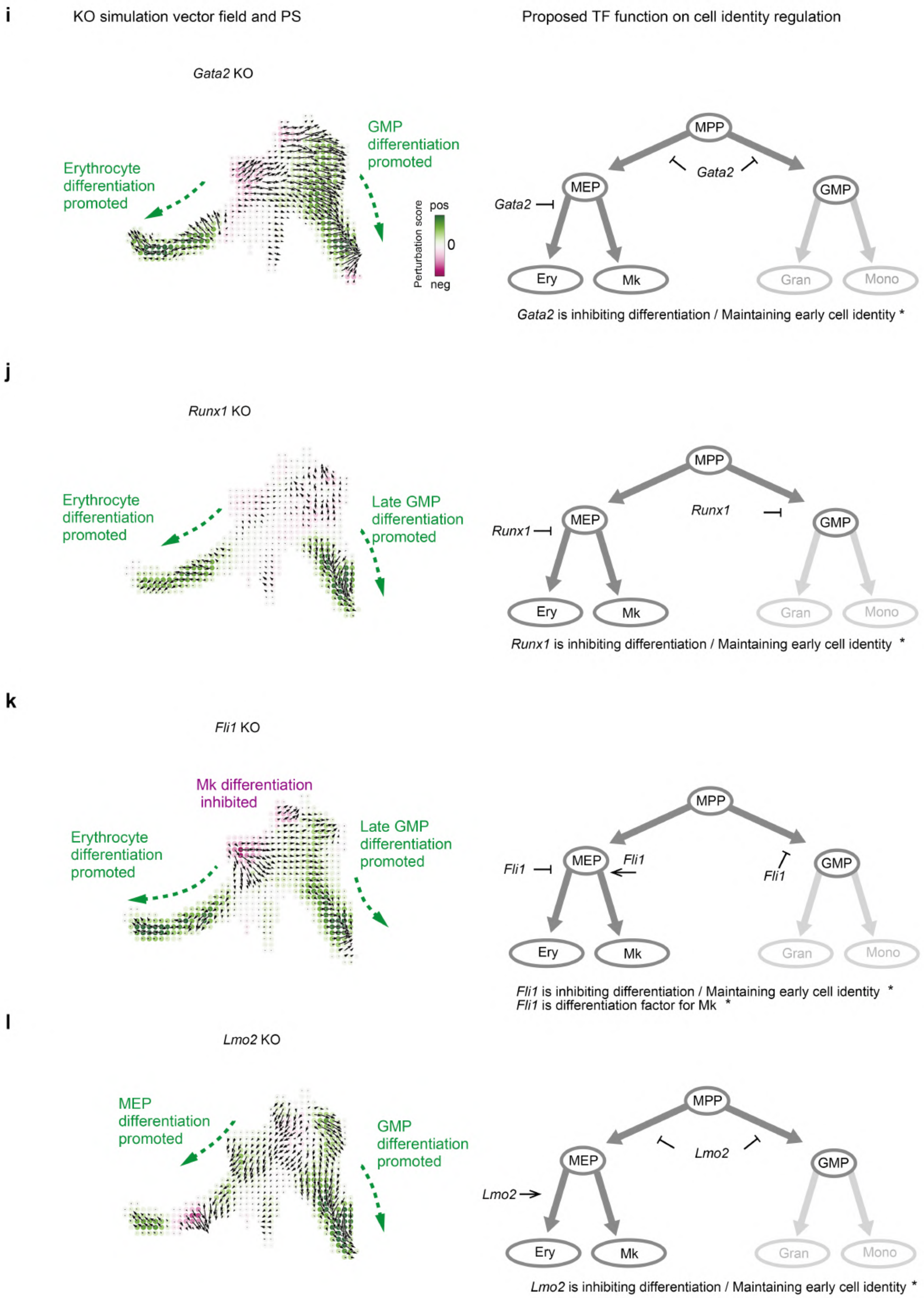

Supplementary Figure 13. CellOracle TF KO simulation results for Dahlin et al. (2018) hematopoiesis data. CellOracle KO simulation results for 12 key TFs: *Spi1/Pu.1*, *Gata1*, *Cebpα*, *Cebpε*, *Eklf/Klf1*, *Gfi1b*, *Fli1*, *Gfi1*, *Gata2*, *Lmo-2*, *Runx1*, and *Irf8* reported in[3] and[4]. The simulated

cell state transition-vector field is visualized with perturbation scores (PS; negative score: magenta, positive score: green). The right column shows a summary of the TF role based on the CellOracle simulation results, cell transition vector, and PS. For example, a positive PS in the TF KO simulation (green) implies that the TF has a role in cell state maintenance or inhibiting cell differentiation. In contrast, a negative PS in the KO simulation (magenta) implies that the TF normally serves a role in promoting cell differentiation. In these figures, the summary of the simulation results is shown in the right column with the mark (*), which indicates that the simulation results agree with the previously reported role or phenotype of the TF.

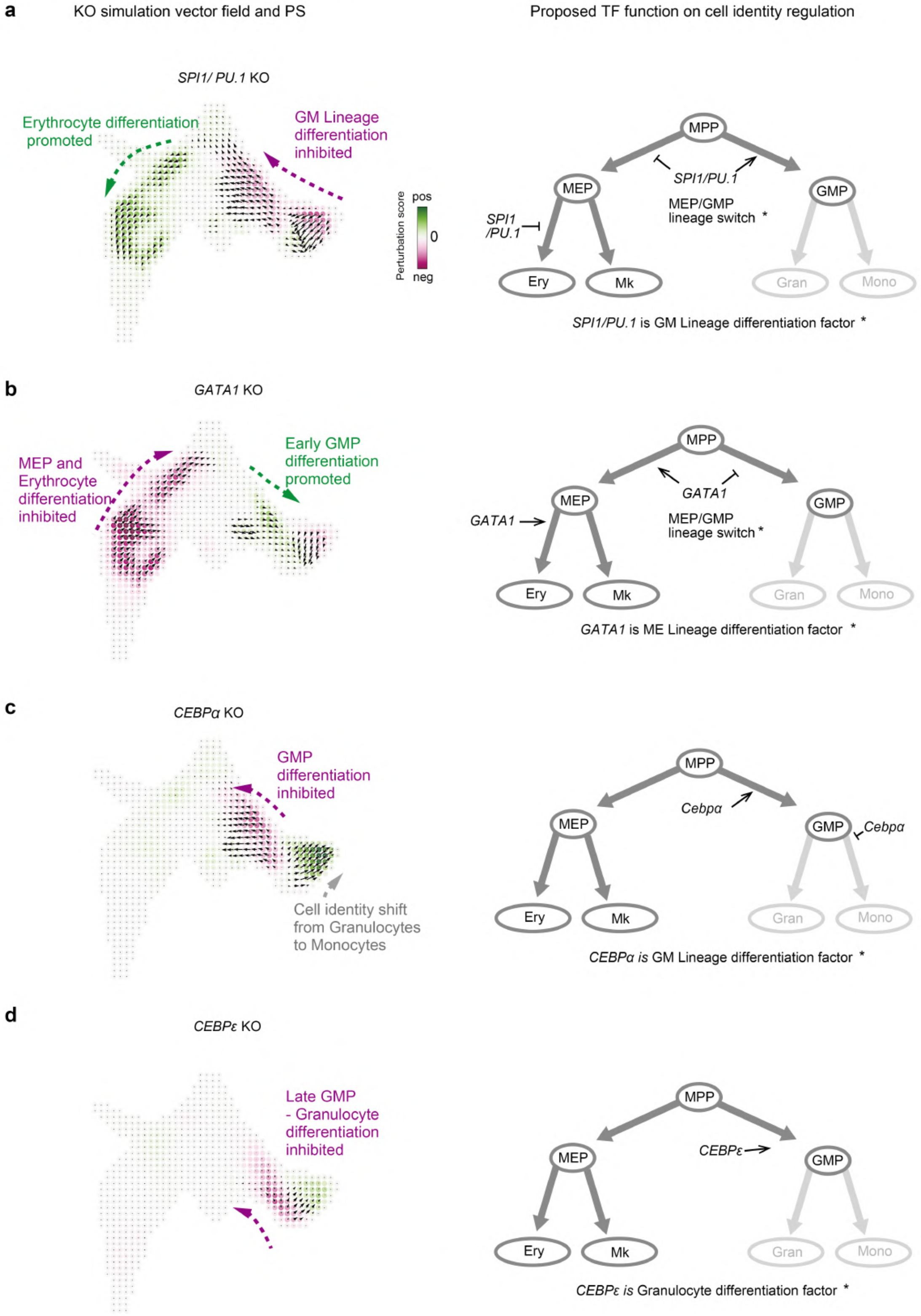

a
KO simulation vector field and PS
Proposed TF function on cell identity regulation
SPI1/ PU.1 KO
Erythrocyte differentiation promoted
GM Lineage differentiation inhibited
Perturbation score
pos
0
neg
MPP
MEP
GMP
SPI1/PU.1
MEP/GMP lineage switch *
SPI1 /PU.1
Ery
Mk
Gran
Mono
SPI1/PU.1 is GM Lineage differentiation factor *
b
GATA1 KO
MEP and Erythrocyte differentiation inhibited
Early GMP differentiation promoted
GATA1
MEP/GMP lineage switch *
GATA1 is ME Lineage differentiation factor *
c
CEBPα KO
GMP differentiation inhibited
Cell identity shift from Granulocytes to Monocytes
Cebpα
CEBPα is GM Lineage differentiation factor *
d
CEBPε KO
Late GMP - Granulocyte differentiation inhibited
CEBPε
CEBPε is Granulocyte differentiation factor *

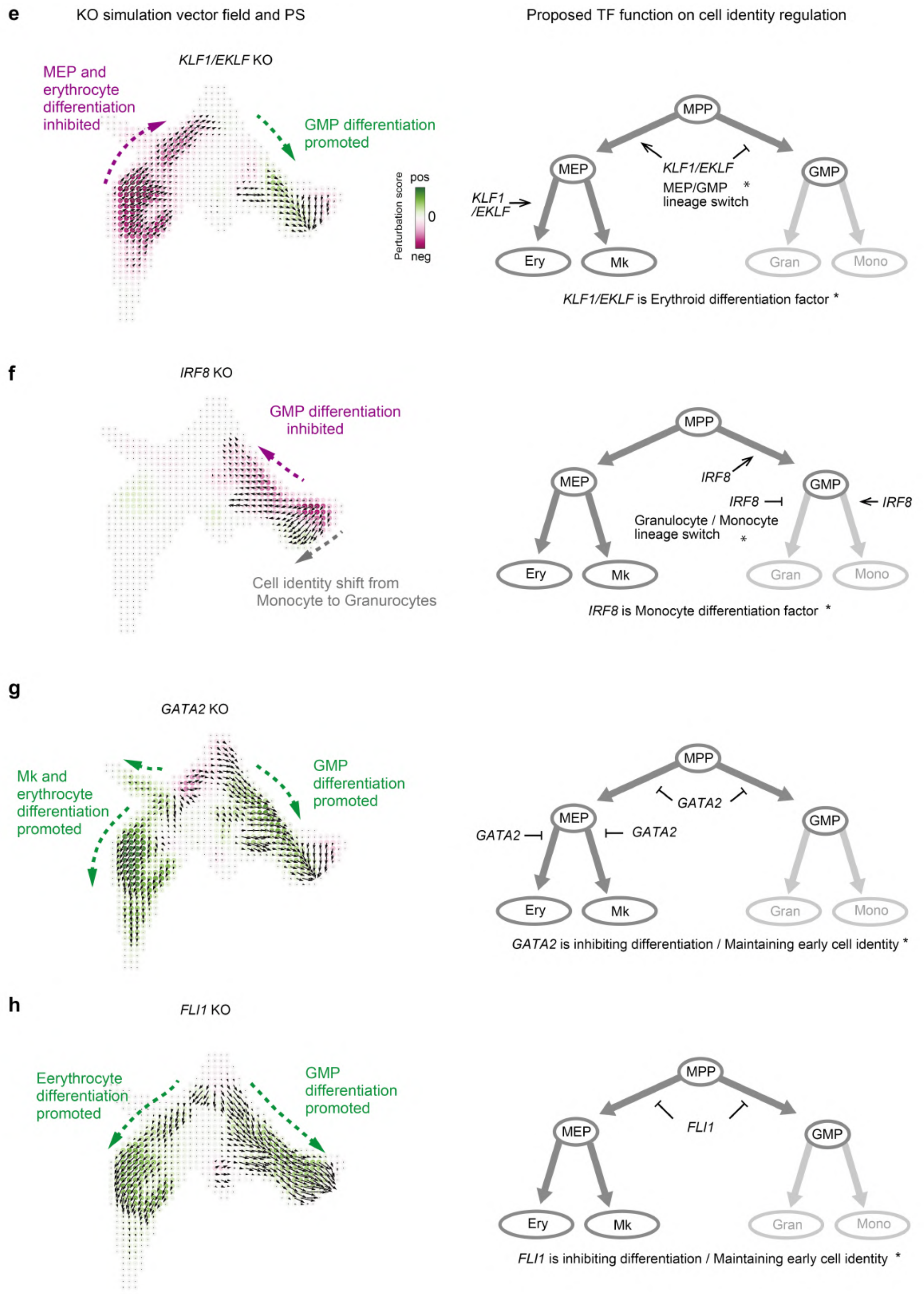

Supplementary Figure 14. CellOracle TF KO simulation results for Setty et al. (2019) hematopoiesis data. CellOracle KO simulation results for *SPI1/PU.1, GATA1, CEBPα, CEBPε,*

EKLF/KLF1, FLI1, GATA2, and *IRF8*, human homologs for eight of the key TFs shown in **Supplementary Fig 13** are analyzed by CellOracle KO simulation. *LMO2, RUNX1, GFI1*, and *GFI1B* could not be analyzed in the Setty et al. hematopoiesis data because *GFI1* and *GFI1B* are not included in the raw data, and *LMO2* and *RUNX1* are filtered out by either the first scRNA-seq preprocessing or subsequent GRN calculation. The Buenrostro et al.[5] human hematopoiesis scATAC-seq data was used for human hematopoiesis base GRN construction. (PS; negative score: magenta, positive score: green). The right column shows a summary of the TF role based on the CellOracle simulation results, cell transition vector, and perturbation score. For example, a positive PS in the TF KO simulation (green) implies that the TF has a role in cell state maintenance or inhibiting cell differentiation. In contrast, a negative PS in the KO simulation (magenta) implies that the TF normally promotes cell differentiation. In these figures, the summary of the simulation results is shown in the right column with the mark (*), which indicates that the simulation results agree with the previously reported role or phenotype of the TF.

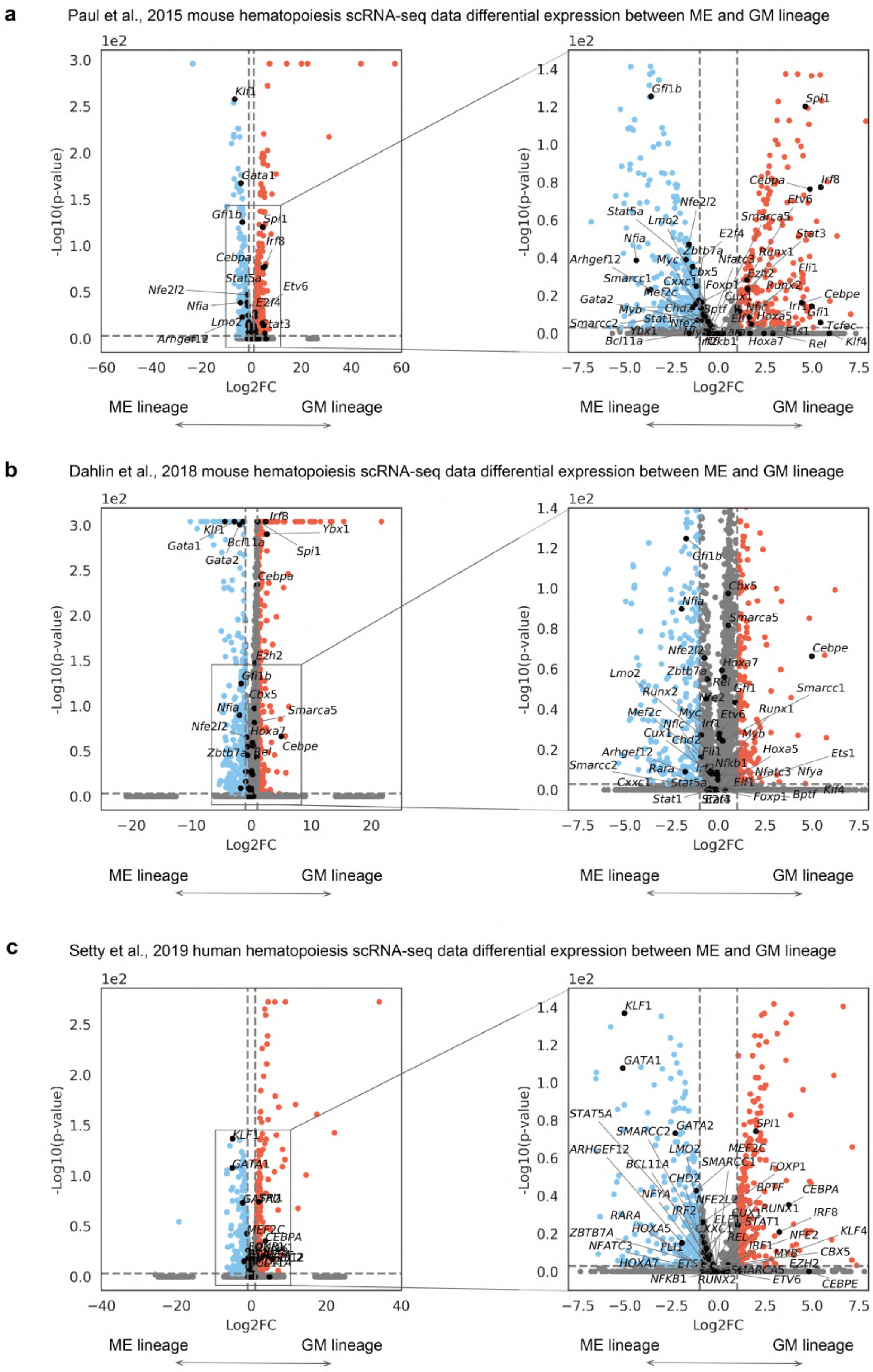

Supplementary Figure 15. Differential expression analysis. Using three independent hematopoiesis datasets in mouse and human, cells were split into ME and GM lineages, and

differential expression (DE) was analyzed by measuring fold change and p-value. Results are visualized as a volcano plot. The right panel shows a magnified area. The x-axis represents log2 fold change. If the gene has more than Log2FC = 1 or less than Log2FC=-1, which indicates a 2-fold difference, the gene was highlighted in blue (upregulated in ME lineage) or red (upregulated in GM lineage). The TFs highlighted by CellOracle analysis (**Fig. 1j**) are shown in black for comparison. The y-axis shows -Log10 p-value of the difference. The statistical test was performed using the two-tailed Wilcoxon rank-sum test with Bonferroni correction. **(a)** DE analysis with Paul et al. 2015 mouse hematopoiesis scRNA-seq data. **(b)** DE analysis with Dahlin et al. 2018 mouse hematopoiesis scRNA-seq data. **(c)** DE analysis with Setty et al. 2019 human hematopoiesis scRNA-seq data.

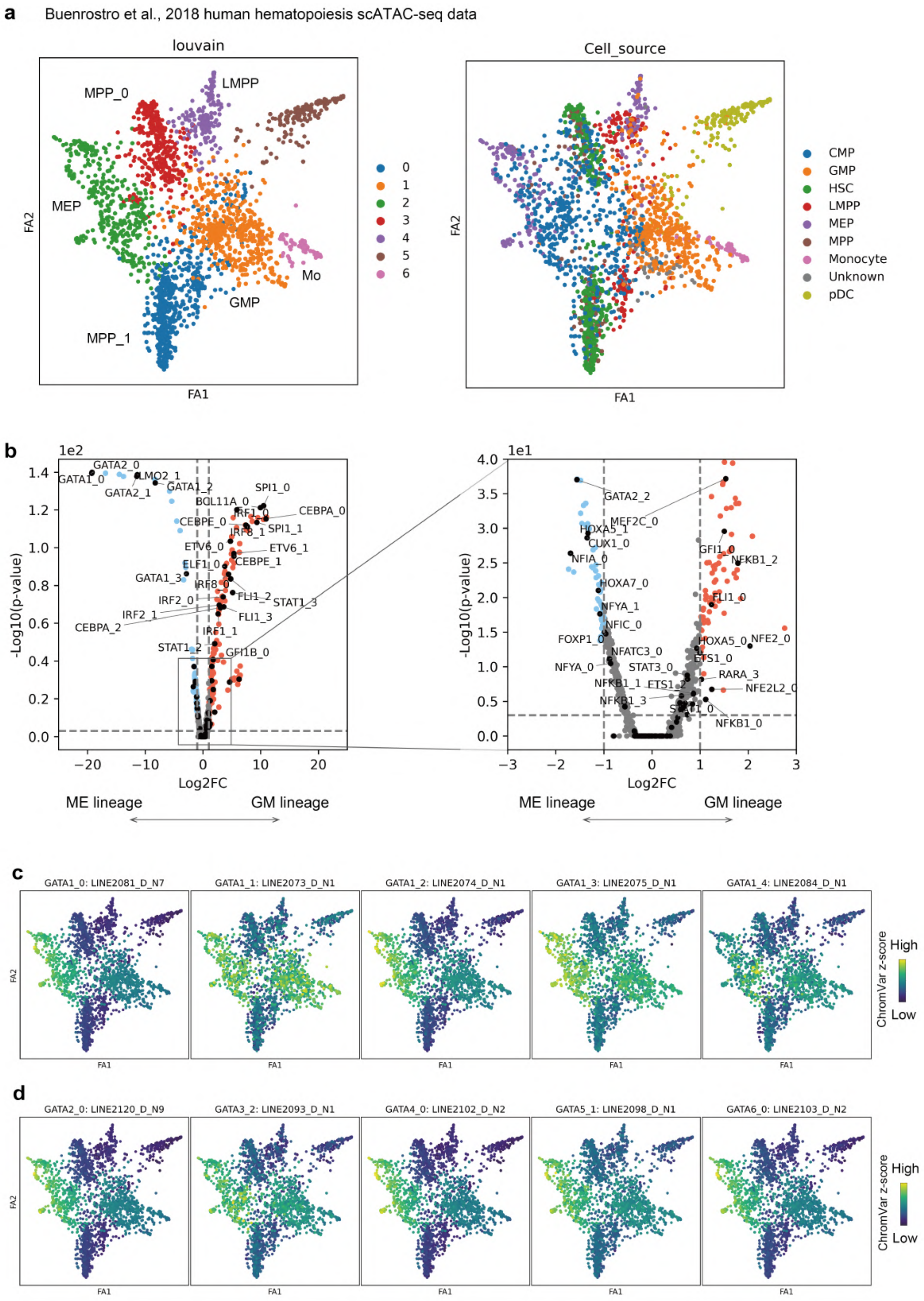

Supplementary Figure 16. scATAC-seq motif enrichment analysis. (a) Force-directed graph of 2,034 cells from Buenrostro et al. 2018 hematopoiesis scATAC-seq data[5], colored with Louvain

clustering (left panel) or cell source information based on FACS gating (right panel). **(b)** ChromVar motif scores are compared between ME and GM lineage clusters. The volcano plot shows the differential motif score between ME and GM lineage cells. The x-axis represents log2 fold change. The gene was highlighted as blue (enriched in ME lineage) or red (enriched in GM lineage) if the gene has a greater than two-fold change and the p-value is less than 0.001. The TFs highlighted by CellOracle analysis (**Fig. 1j**) are shown in black for comparison. The statistical test was performed using the two-tailed Wilcoxon rank-sum test with Bonferroni correction. A gene can have multiple motifs. **(c)** GATA1 motifs projected onto the force-directed graph. **(c)** GATA family motifs projected onto the force-directed graph.

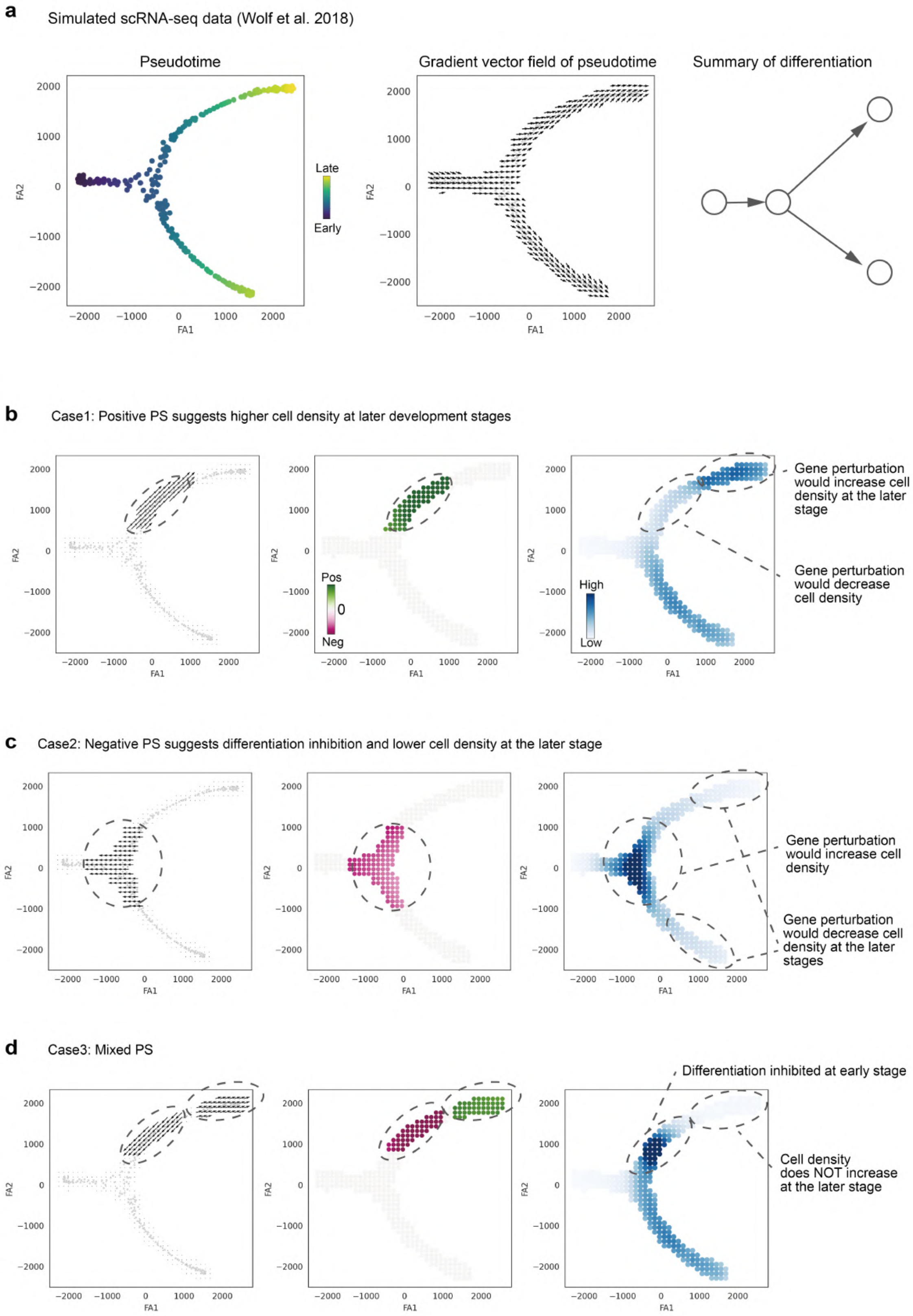

Supplementary Figure 17. Markov simulation. A Markov random walk simulation was performed as previously reported[6] with some modifications (**Methods**). **(a)** Simulated scRNA-

seq data[7] was analyzed to illustrate a typical Markov random walk simulation. The data were downloaded and processed with Scanpy. The cells show a Y-shape differentiation trajectory, bifurcating into two branches. **(b-d)** Markov simulations were performed based on several artificial perturbation vectors: **(b)** The perturbation vector shares the same direction as the differentiation vector. The area highlighted with the dashed line indicates the area that has positive PS values. The results of the random walk simulation based on the perturbation vector (right panel) show that the perturbation increases cell density in later stages while it decreases cell density within the positive PS area due to the promotion of differentiation. **(c)** If the perturbation vector opposes the differentiation vector, it would induce cell differentiation inhibition or arrest, where cell density is high at the point of inhibited differentiation, resulting in the loss of cells at later stages. **(d)** Markov simulation analysis with the oscillating perturbation vectors. The earlier negative PS will have dominant effects, leading to cell differentiation inhibition at an early stage; thus, the later promotion of differentiation would not impact cell density.

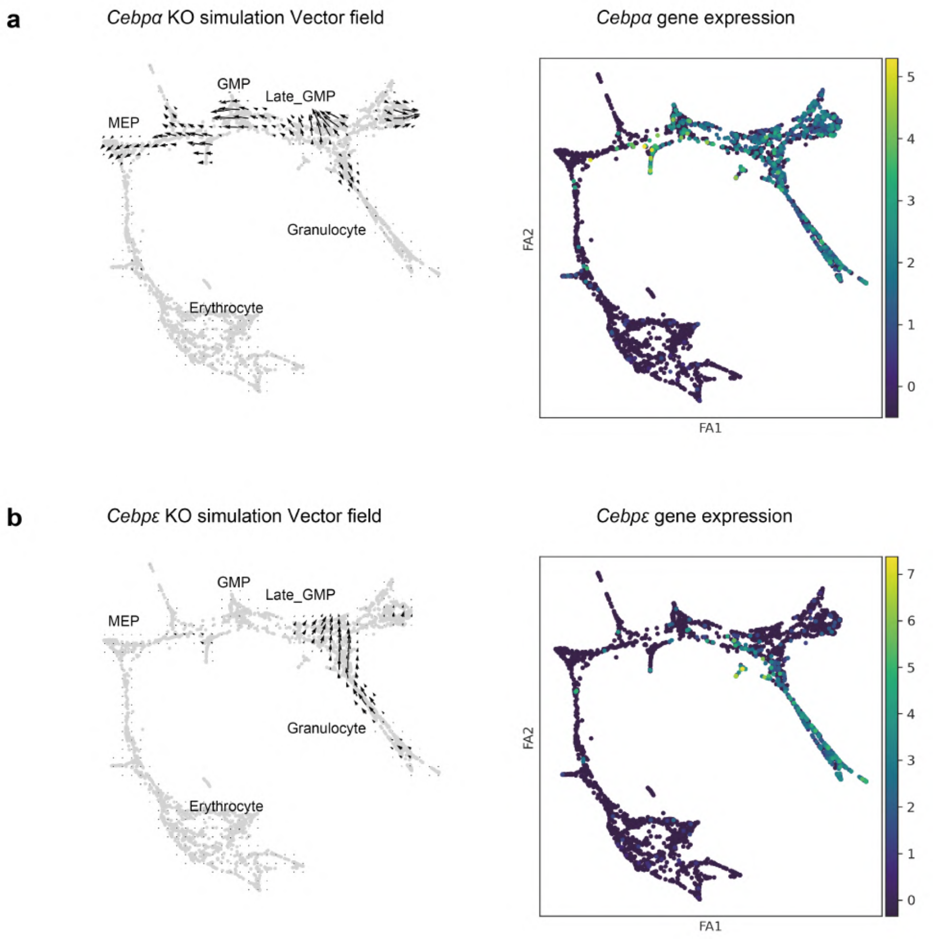

Supplementary Figure 18. CellOracle cell identity shift simulation vector and expression of *Cebpα* and *Cebpε*. (a) Cell identity shift vector of *Cebpα* KO simulation (left) and *Cebpα* gene expression (log-transformed UMI). **(b)** Cell identity shift vector of *Cebpε* KO simulation (left) and *Cebpε* gene expression (log-transformed UMI). It is notable that the KO vector and gene expression exhibit different patterns. For example, the *Cebpε* KO vector and experimental KO cell density showed the most prominent change from the late GMP to the early granulocyte entry point. In contrast, gene expression is not high at this point, suggesting that the gene expression pattern alone is not enough to predict the KO phenotypes highlighted in this analysis.

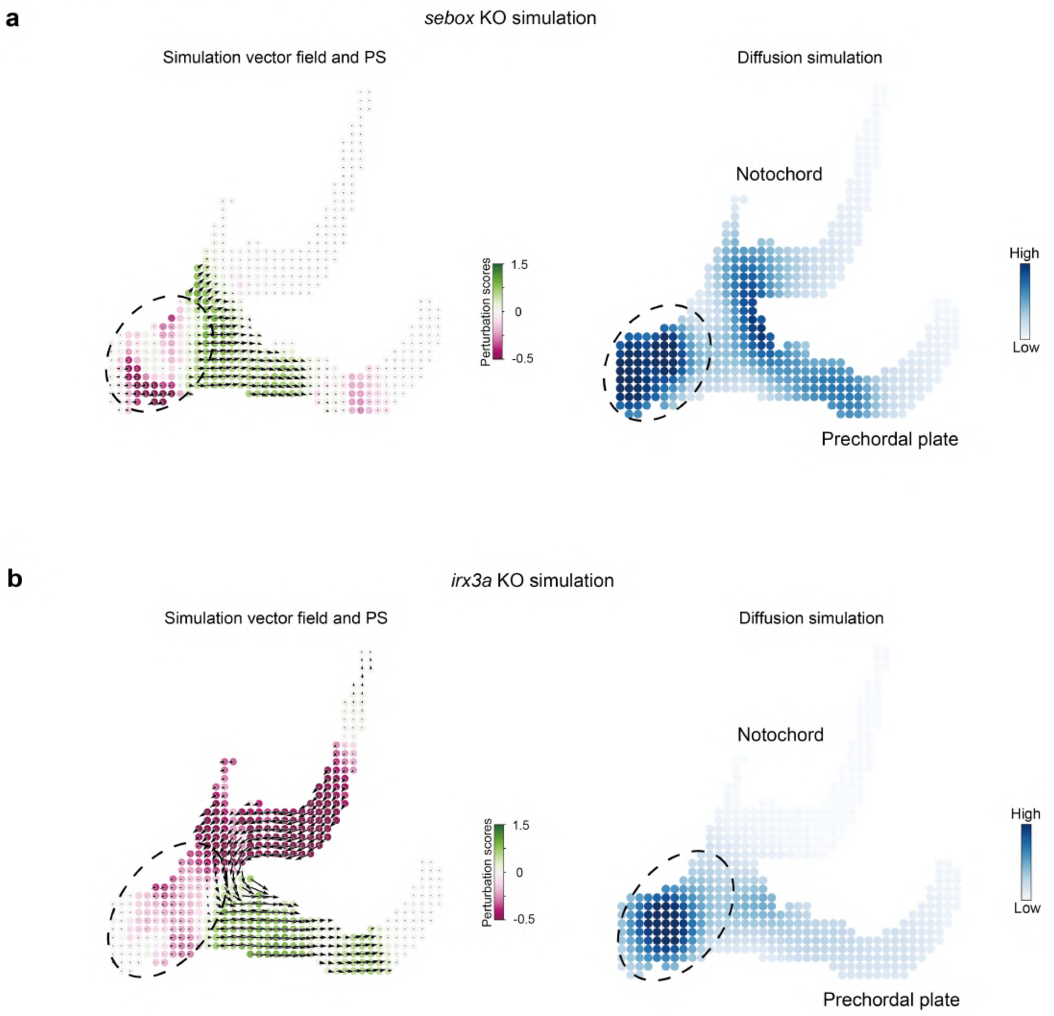

Supplementary Figure 19. CellOracle KO simulation for *sebox* and *irx3a*. (a, b) CellOracle cell identity shift vector field and perturbation score for *sebox* and *irx3a* KO simulation (left) and Markov simulation results. The Markov diffusion simulation suggests that cell differentiation is inhibited in the early stages of axial mesoderm differentiation (highlighted by the dashed circle).

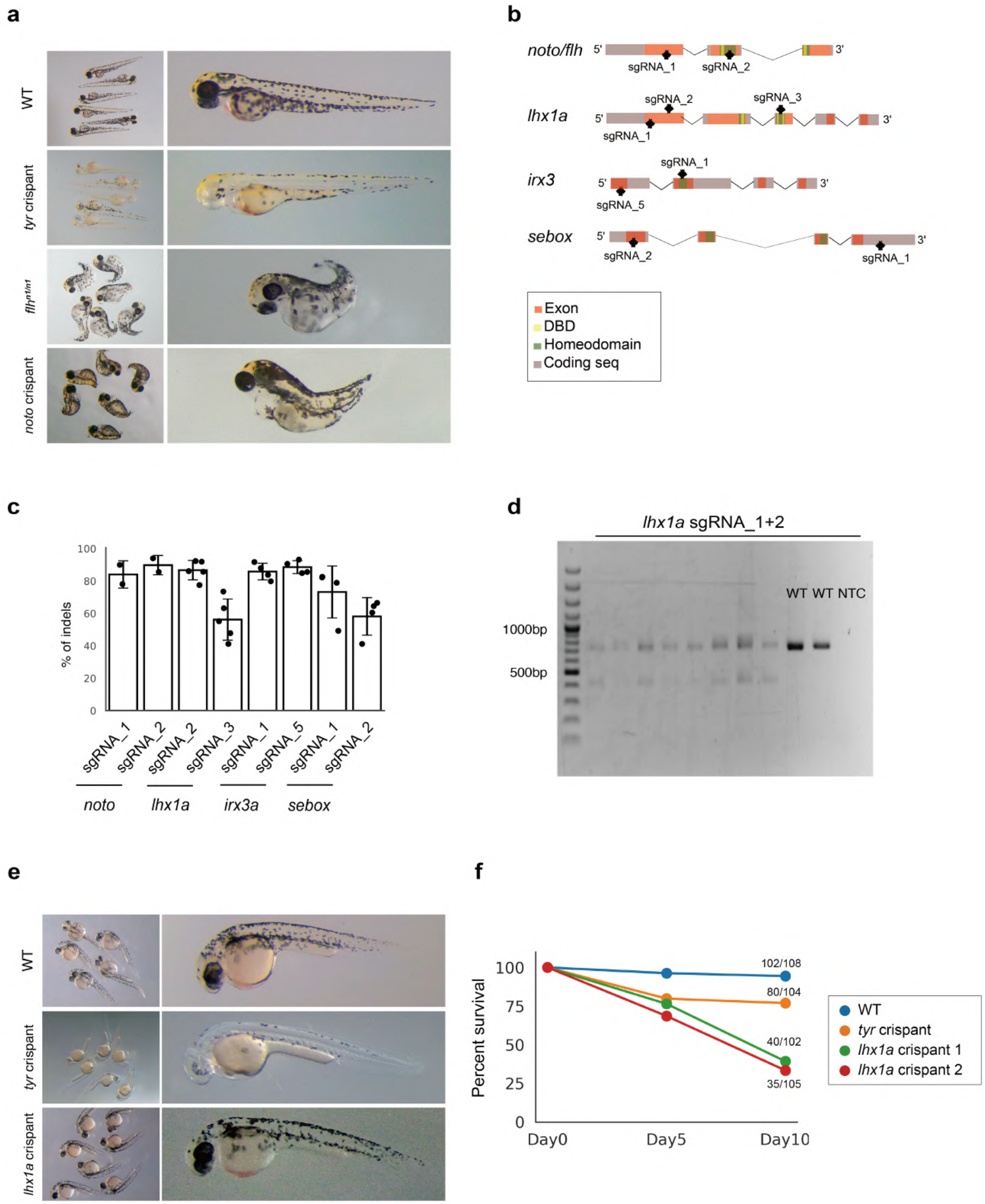

Supplementary Figure 20. Zebrafish mutant morphology, genome editing efficiency, and survival rate. (a) The morphological phenotype of WT zebrafish, *flh*$^{n1/n1}$ mutant, *tyr* crispant, and *noto/flh* crispant at 2 days post fertilization (dpf). **(b)** Schematic representation of sgRNAs designed based on CHOPCHOP prediction and target functional domain. **(c)** Genome editing

efficiency was analyzed by TIDE in embryos injected with RNPs. The bar plot shows mean ± SD from n=2 embryos for *flh*$^{n1/n1}$, and n=4 embryos for *lhx1a, sebox,* and *irx3a* crispants. **(d)** *lhx1a* sgRNA_1 and sgRNA_2 genome editing validation with gel electrophoresis. Although *lhx1a* sgRNA_1 alone did not induce indels, we found a combination of *lhx1a* sgRNA_1 and sgRNA_2 induced deletions in the target locus. The gel image is the representation of more than 3 biological replicates. Tests were repeated at least 3 times. **(e)** Morphology of *tyr* and *lhx1a* crispants at 1 dpf. The embryo images are representative of more than 50 embryos. These experiments were repeated at least 3 times. **(f)** Percentage survival at 0, 5, and 10 days post-fertilization (dpf). The number of embryos analyzed is shown in the plot.

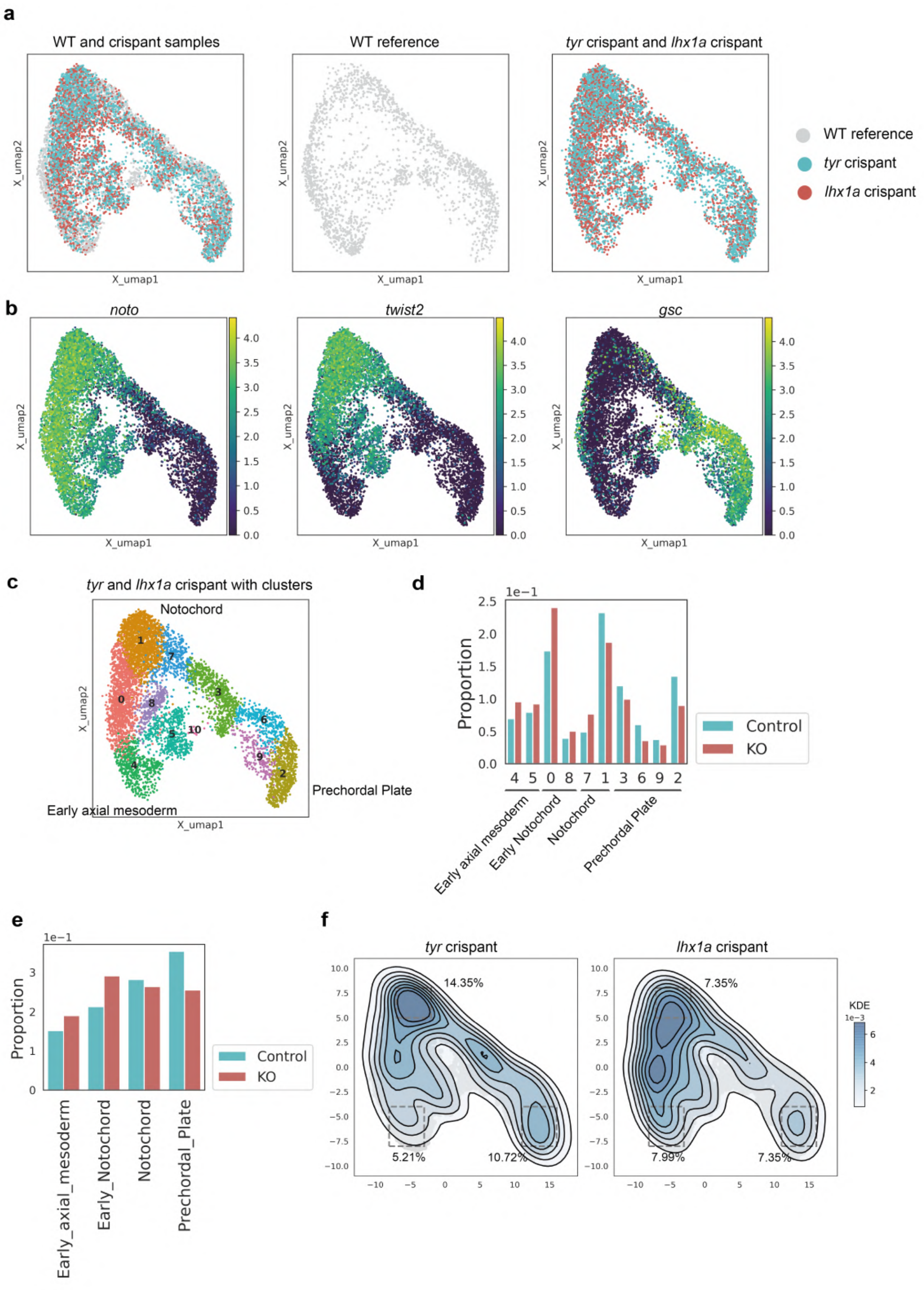

Supplementary Figure 21. Zebrafish scRNA-seq experiments for *lhx1a* LOF analysis without data transfer. (a) UMAP plot of integrated axial mesoderm data consists of *lhx1a* crispant, *tyr* control crispant, and WT reference samples. The UMAP plot is calculated with integrated data. **(b)** Gene expression of *noto*, *twist2*, and *gsc2* projected onto the UMAP plot (log-transformed UMI). **(c)** Louvain cell clustering results projected onto the UMAP plot. **(d)** Cell cluster

composition comparing *lhx1a* crispant and *tyr* control crispant samples. **(e)** Cells are grouped by several clusters shown in d, and cell composition is compared between *lhx1a* crispant and *tyr* control crispant samples. **(f)** Control cell density (left, n=2,342 cells) and lhx1a crispant cell density (right, n=2,502 cells) are shown in the kernel cell density contour plot. A Scatter plot of whole integrated data is shown as background (light gray) to highlight the overall cell trajectory structure.

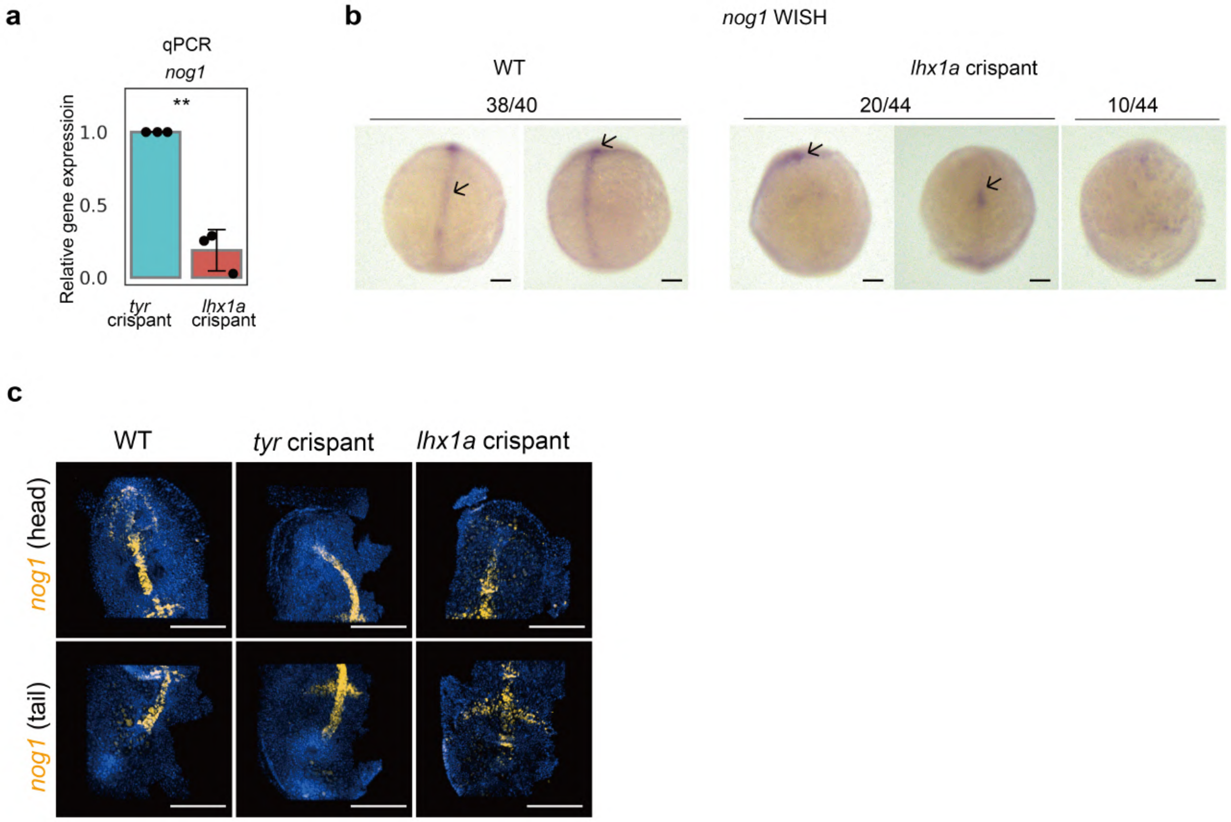

Supplementary Figure 22. Zebrafish qRT-PCR and WISH. (a) qRT-PCR for *nog1* (normalized to rpl13a) at 10 hpf in *lhx1a* and *tyr* crispants. *Nog1* expression is significantly depleted in *lhx1a* crispants (n=3 independent biological replicates, 3 technical replicates each, 50 embryos per sample; p=4.99 x10^{-3}, paired t-test, one-tailed). Data are presented as mean +/- the standard deviation value. **(b)** WISH for *nog1* in WT and *lhx1a* crispant embryos at 10 hpf. Fractions indicate the number of embryos with the pictured phenotype over the number of embryos examined. The experiment is representative of 2 independent biological replicates. Scale bar = 150 µm. (**c**) Representative flattened HCR images of 10 hpf embryos stained with probes against *nog1* (yellow), nuclei stained with DAPI (blue). Scale bar = 300 µm. These images are representative of n=8 embryos for each condition. The experiment was repeated twice.

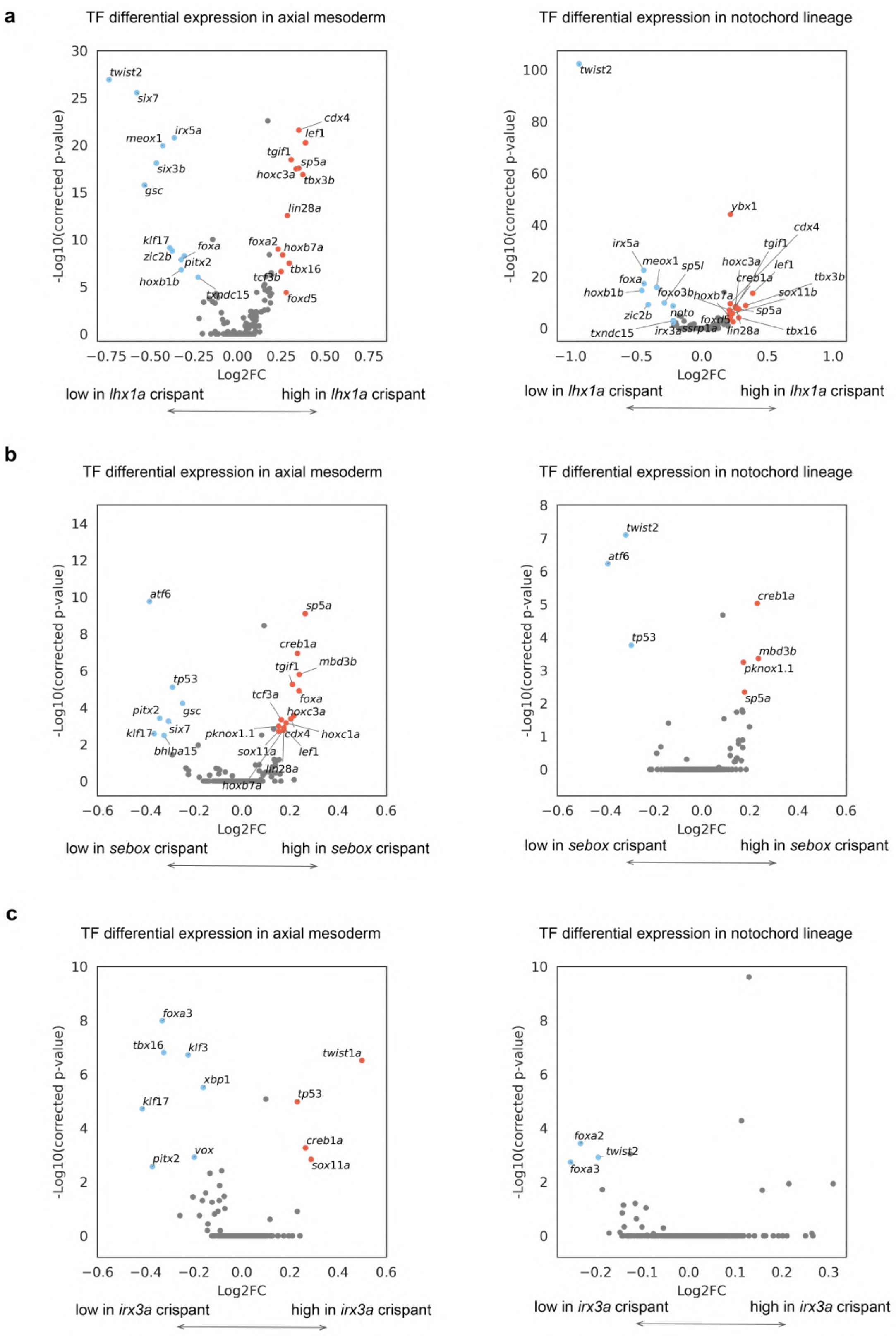

Supplementary Figure 23. Differential transcription factor expression in *lhx1a*, *sebox*, and *irx3a* crispants. TF gene expression in *lhx1a*, *sebox*, and *irx3a* crispant samples compared with control

tyr crispant samples. All TFs in the zebrafish promoter base GRN were selected, and differential gene expression (DE) was calculated. The left panel shows DE calculated with whole axial mesoderm clusters, while the right panel shows the DE in the notochord lineage, including Notochord, Early_Notochord, and Early_axial_mesoderm clusters. The x-axis represents log2 fold change (Log2FC). If the gene has more than Log2FC = 0.25 or less than Log2FC=-0.25, it is highlighted as blue or red. The y-axis shows -Log10 p-value of the difference. The statistical test was performed using the two-tailed Wilcoxon rank-sum test with Bonferroni correction. **(a)** TF DE analysis comparing *lhx1a* crispant with *tyr* crispant samples. **(b)** TF DE analysis comparing *sebox* crispant with *tyr* crispant samples. **(c)** TF DE analysis comparing *irx3a* crispant with *tyr* crispant samples.

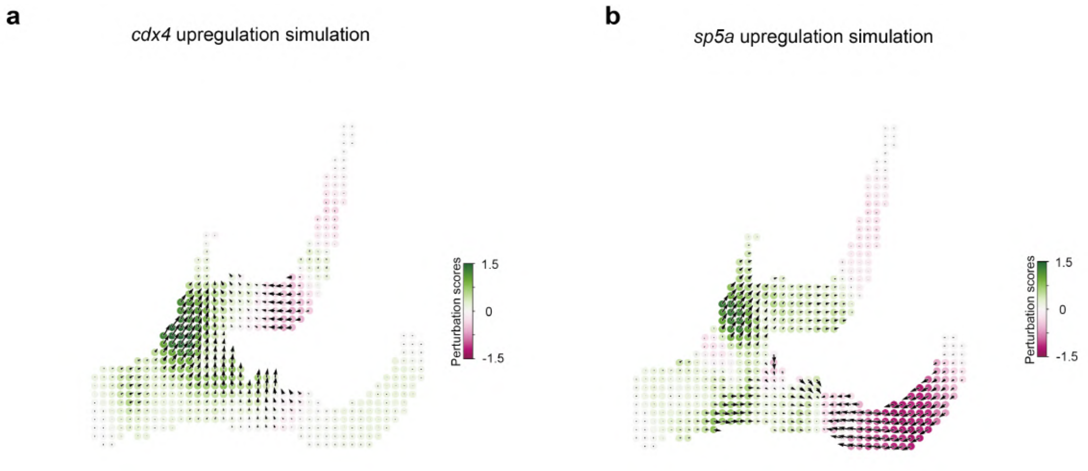

Supplementary Figure 24. CellOracle upregulation simulation with Farrell et al. 2018 zebrafish scRNA-seq data. CellOracle TF perturbation simulation is performed for the upregulation of *cdx4* and *sp5a*, which are slightly upregulated in the axial mesoderm of *lhx1a* and *sebox* crispant samples. *lhx1a* and *sebox* expression values are set to the maximum value of wild-type cell gene expression. Cell identity shift vector field and perturbation score are visualized together. (**a**) *Cdx4* upregulation simulation predicts that Notochord differentiation is promoted. (**b**) *Sp5a* upregulation simulation predicts that Notochord differentiation is promoted while Prechordal_Plate differentiation is inhibited.

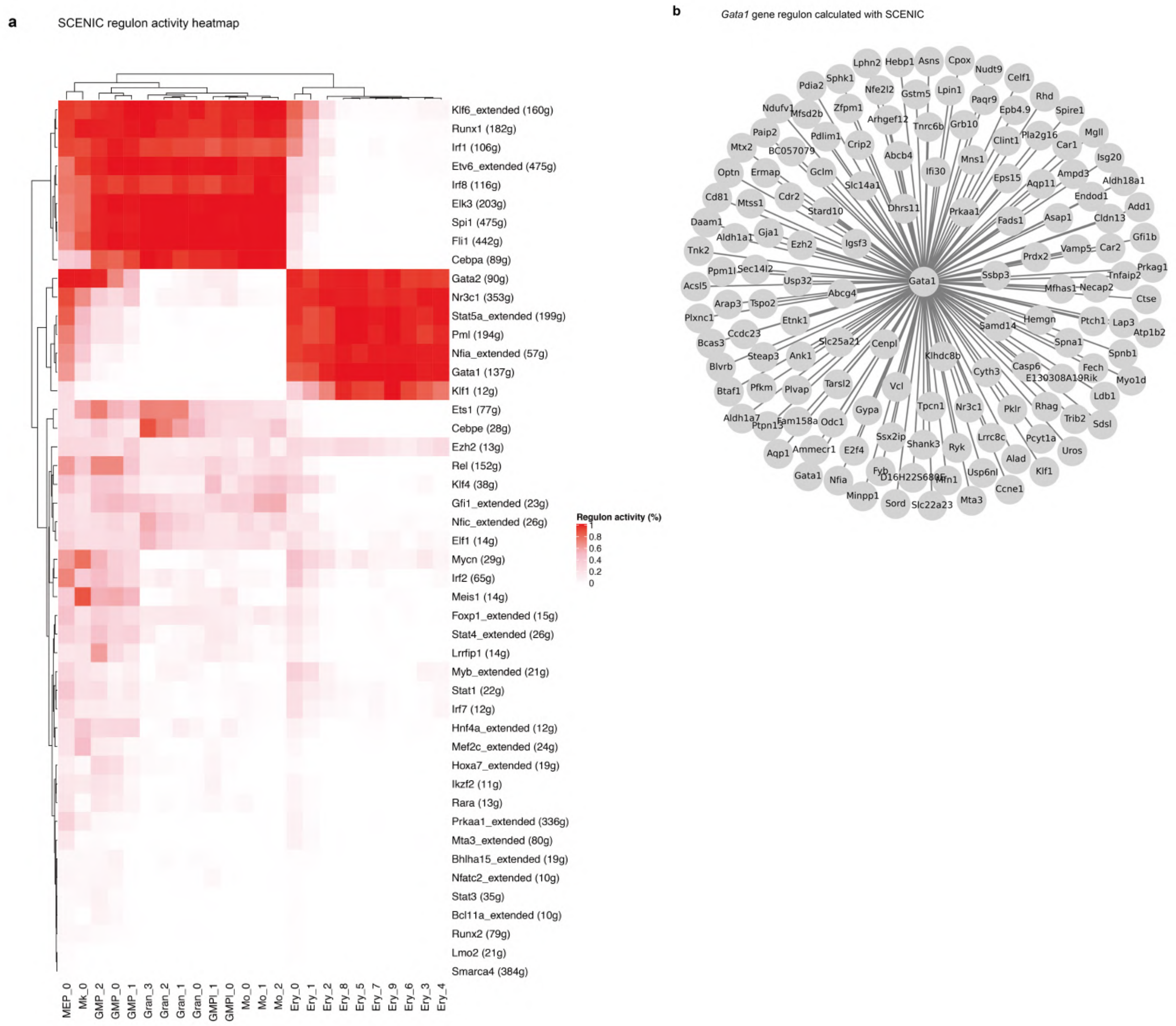

Supplementary Figure 25. SCENIC analysis with Paul et al. 2015 hematopoiesis scRNA-seq data. SCENIC regulon analysis is performed with 2,730 myeloid progenitor cell scRNA-seq data from Paul et al. (2015)[1]. The same cell-type annotation is used in the CellOracle analysis in Figure 2. (**a**) SCENIC Regulon activity score is shown as a heatmap. (**b**) SCENIC regulon structure. *Gata1* regulon structure is visualized.

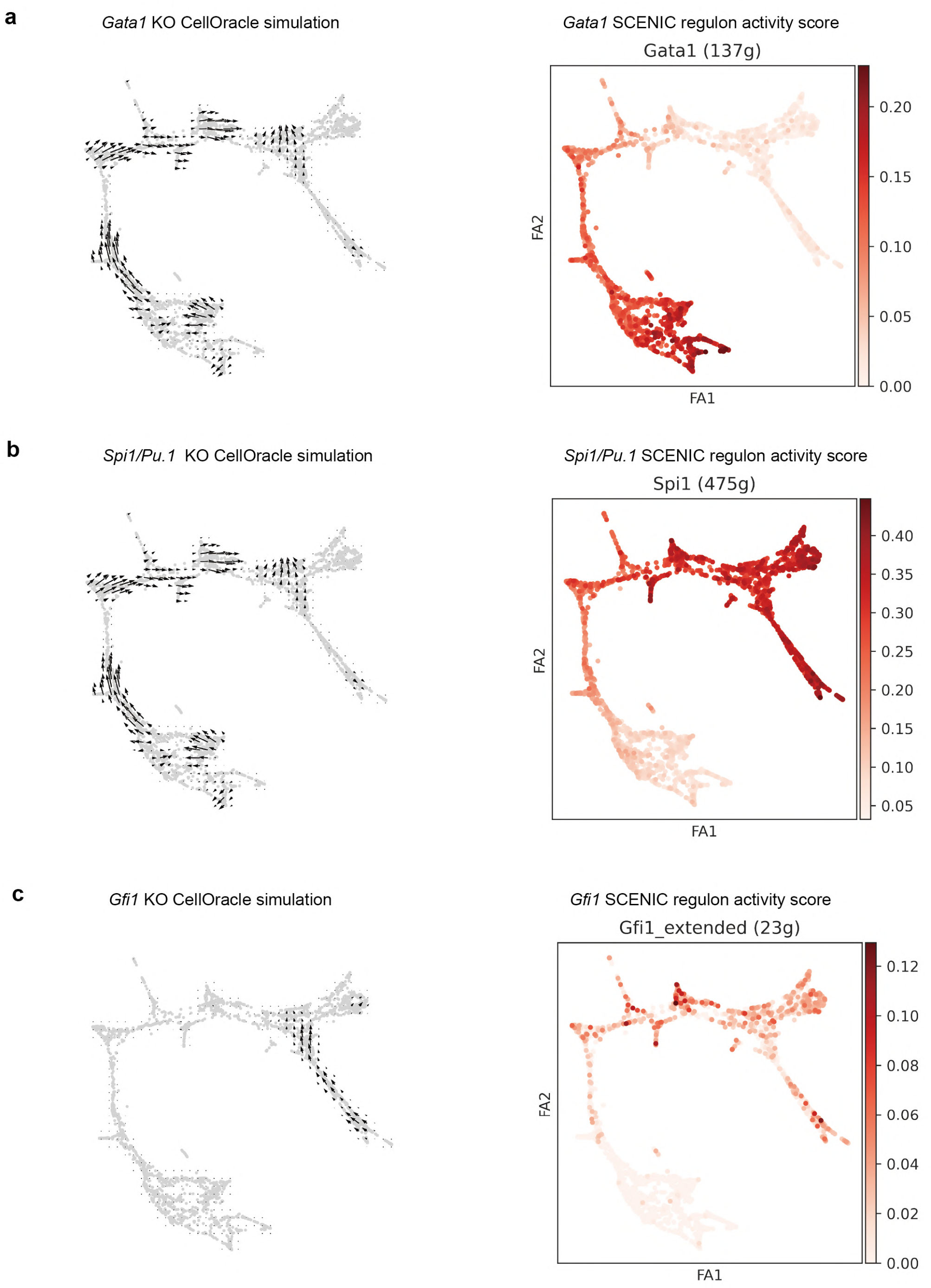
a
Gata1 KO CellOracle simulation
Gata1 SCENIC regulon activity score
Gata1 (137g)
FA2
FA1
0.20
0.15
0.10
0.05
0.00
b
Spi1/Pu.1 KO CellOracle simulation
Spi1/Pu.1 SCENIC regulon activity score
Spi1 (475g)
FA2
FA1
0.40
0.35
0.30
0.25
0.20
0.15
0.10
0.05
c
Gfi1 KO CellOracle simulation
Gfi1 SCENIC regulon activity score
Gfi1_extended (23g)
FA2
FA1
0.12
0.10
0.08
0.06
0.04
0.02
0.00

d

Klf1 KO CellOracle simulation

Klf1 SCENIC regulon activity score

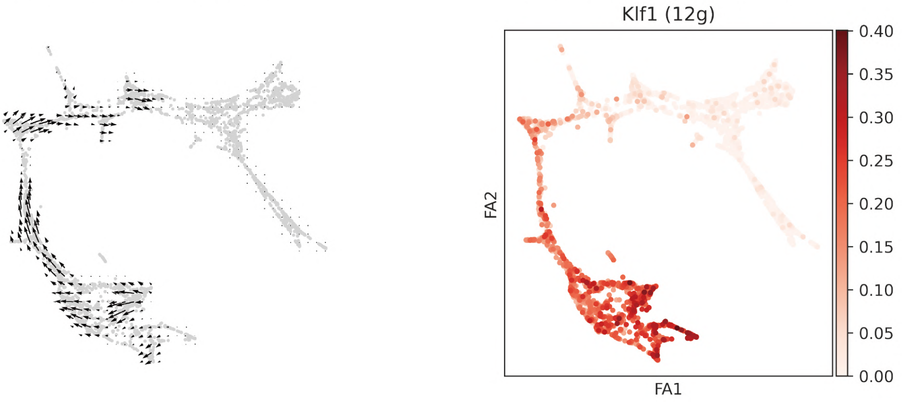

e

Gfi1b KO CellOracle simulation

Gfi1b SCENIC regulon activity score

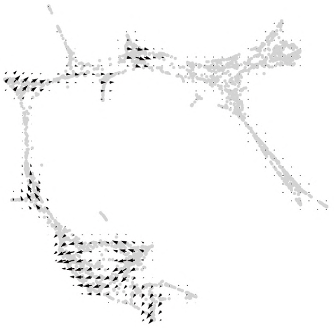

na

f

Irf8 KO CellOracle simulation

Irf8 SCENIC regulon activity score

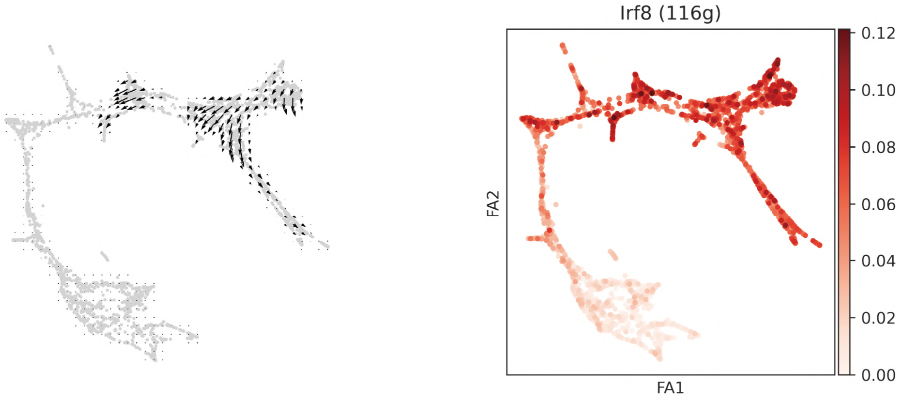

g

Cebpα KO CellOracle simulation

Cebpα SCENIC regulon activity score

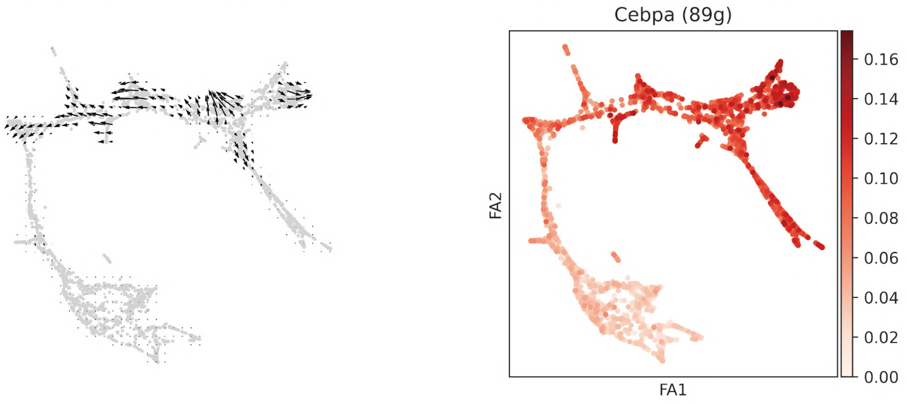

h

Cebpε KO CellOracle simulation

Cebpε SCENIC regulon activity score

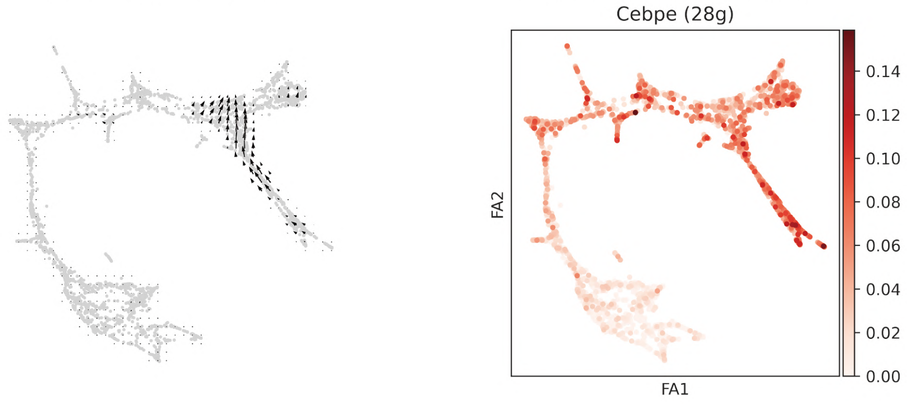

i

Gata2 KO CellOracle simulation

Gata2 SCENIC regulon activity score

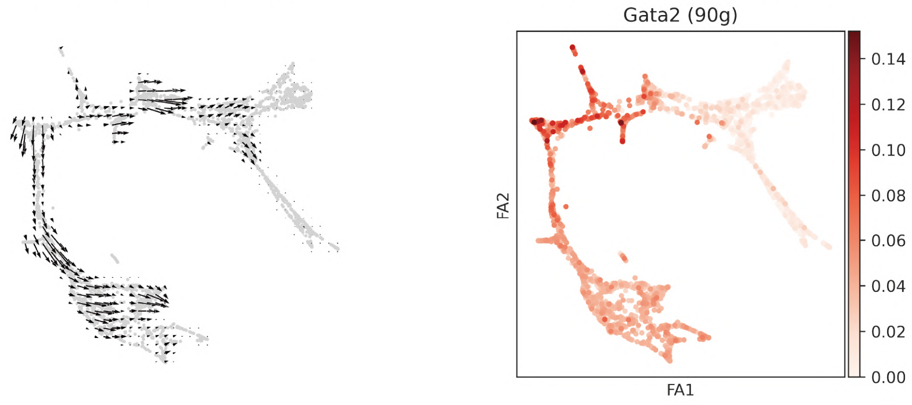

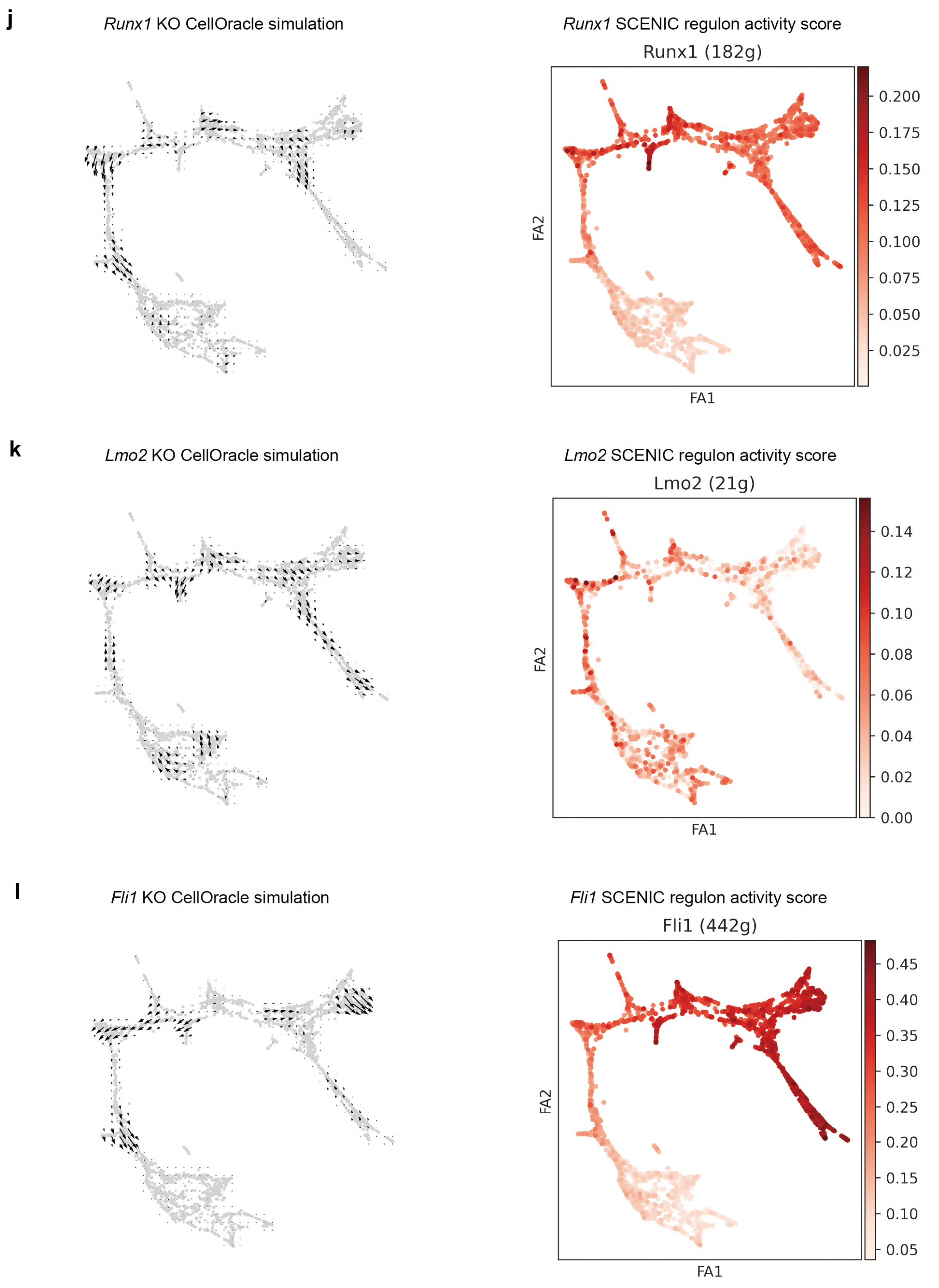

Supplementary Figure 26. Comparison of SCENIC analysis and CellOracle simulation results. SCENIC regulon activity analysis outputs and CellOracle gene perturbation simulation results are compared. CellOracle gene perturbation results are shown as a vector field graph (left panels). The SCENIC regulon activity score is projected onto the same force-directed graph embedding used in the CellOracle analysis (right panels). Although the output of CellOracle's perturbation vector is distinct from SCENIC's scalar regulon values, some of the differences in these results could arise from the motif database used, low effect size, or variation due to pre-processing.

Supplementary References

1. Paul, F. *et al.* Transcriptional Heterogeneity and Lineage Commitment in Myeloid Progenitors. *Cell* **163**, 1663–77 (2015).
2. Cusanovich, D. A. *et al.* A Single-Cell Atlas of In Vivo Mammalian Chromatin Accessibility. *Cell* **174**, 1309–1324 (2018).
3. Orkin, S. H. & Zon, L. I. Hematopoiesis: an evolving paradigm for stem cell biology. *Cell* **132**, 631–44 (2008).
4. Rosenbauer, F. & Tenen, D. G. Transcription factors in myeloid development: balancing differentiation with transformation. *Nature Reviews Immunology 2007 7:2* **7**, 105–117 (2007).
5. Buenrostro, J. D. *et al.* Integrated Single-Cell Analysis Maps the Continuous Regulatory Landscape of Human Hematopoietic Differentiation. (2018) doi:10.1016/j.cell.2018.03.074.
6. la Manno, G. *et al.* RNA velocity of single cells. *Nature* **560**, 494–498 (2018).
7. Wolf, F. A., Angerer, P. & Theis, F. J. SCANPY: large-scale single-cell gene expression data analysis. *Genome Biol* **19**, 15 (2018).
