# Supplementary material for: Dissecting cell identity via network inference and in silico gene perturbation
Source: Nature. 2023 Feb 8;614(7949):742–51. doi: 10.1038/s41586-022-05688-9 (PMC9946838; doi:10.1038/s41586-022-05688-9)
Supplement: Supplementary file 2 — Reporting Summary [file 41586_2022_5688_MOESM2_ESM.pdf]

nature portfolio

Corresponding author(s): Samantha A Morris

Last updated by author(s): Dec 19, 2022

Reporting Summary

Nature Portfolio wishes to improve the reproducibility of the work that we publish. This form provides structure for consistency and transparency in reporting. For further information on Nature Portfolio policies, see our Editorial Policies and the Editorial Policy Checklist.

Statistics

For all statistical analyses, confirm that the following items are present in the figure legend, table legend, main text, or Methods section.

n/a	Confirmed	
☐	☒	The exact sample size (*n*) for each experimental group/condition, given as a discrete number and unit of measurement
☐	☒	A statement on whether measurements were taken from distinct samples or whether the same sample was measured repeatedly
☐	☒	The statistical test(s) used AND whether they are one- or two-sided<br>*Only common tests should be described solely by name; describe more complex techniques in the Methods section.*
☒	☐	A description of all covariates tested
☐	☒	A description of any assumptions or corrections, such as tests of normality and adjustment for multiple comparisons
☐	☒	A full description of the statistical parameters including central tendency (e.g. means) or other basic estimates (e.g. regression coefficient) AND variation (e.g. standard deviation) or associated estimates of uncertainty (e.g. confidence intervals)
☐	☒	For null hypothesis testing, the test statistic (e.g. *F*, *t*, *r*) with confidence intervals, effect sizes, degrees of freedom and *P* value noted<br>*Give P values as exact values whenever suitable.*
☐	☒	For Bayesian analysis, information on the choice of priors and Markov chain Monte Carlo settings
☐	☒	For hierarchical and complex designs, identification of the appropriate level for tests and full reporting of outcomes
☐	☒	Estimates of effect sizes (e.g. Cohen's *d*, Pearson's *r*), indicating how they were calculated

Our web collection on statistics for biologists contains articles on many of the points above.

Software and code

Policy information about availability of computer code

Data collection	1. The Cell Ranger v5.0.1 pipeline (https://support.10xgenomics.com/single-cell-gene-expression/software/downloads/latest) was used to process data collected using the 10x Chromium platform.<br>2. sgRNAs were designed with CHOPCHOP (http://chopchop.cbu.uib.no/)
Data analysis	1: CellOracle (https://github.com/morris-lab/CellOracle)<br>2. HOMER (http://homer.ucsd.edu/homer/)<br>3. gimmemotifs (https://gimmemotifs.readthedocs.io/en/master/)<br>4. CisBP version2 database (http://cisbp.ccbr.utoronto.ca)<br>5. Scanpy (https://scanpy.readthedocs.io/en/stable/)<br>6. Seurat (https://satijalab.org/seurat/)<br>7. scikit-learn (https://scikit-learn.org/stable/)<br>8. igraph (https://igraph.org)<br>9. SCENIC (https://github.com/aertslab/SCENIC)<br>10. FASTQC quality check (https://www.bioinformatics.babraham.ac.uk/projects/fastqc/)<br>11. IMARIS 9.9 software<br>12. g:Profiler API (https://biit.cs.ut.ee/gprofiler/page/apis)

For manuscripts utilizing custom algorithms or software that are central to the research but not yet described in published literature, software must be made available to editors and reviewers. We strongly encourage code deposition in a community repository (e.g. GitHub). See the Nature Portfolio guidelines for submitting code & software for further information.

Data

Policy information about availability of data

All manuscripts must include a data availability statement. This statement should provide the following information, where applicable:

- Accession codes, unique identifiers, or web links for publicly available datasets
- A description of any restrictions on data availability
- For clinical datasets or third party data, please ensure that the statement adheres to our policy

All source data, including sequencing reads and single-cell expression matrices, are available from the Gene Expression Omnibus (GEO) under accession codes GSE7285916, GSE11282432, and GSE145298 for the zebrafish profiling from this study; from ArrayExpress under accession codes E-MTAB-7325 (Tal1−/− chimeras), and E-MTAB-7324 (wild-type chimeras). Simulations can be explored at celloracle.org.

Field-specific reporting

Please select the one below that is the best fit for your research. If you are not sure, read the appropriate sections before making your selection.

☒ Life sciences ☐ Behavioural & social sciences ☐ Ecological, evolutionary & environmental sciences

For a reference copy of the document with all sections, see nature.com/documents/nr-reporting-summary-flat.pdf

Life sciences study design

All studies must disclose on these points even when the disclosure is negative.

Sample size	For GRN inference, a minimum of 50 cells from scRNA-seq was determined via benchmarking. For scRNA-seq experiments and validation, a minimum of 3 independent biological replicates was used as the sample size. No methods to predetermine the sample size were used. A sample size of a minimum of 3 was rationalized to be sufficient as the size of the cell populations under study were large enough to sufficiently power the analysis.
Data exclusions	No data were excluded from the analyses.
Replication	Independent biological replicates were performed to determine reproducibility and no results have been omitted.
Randomization	Randomization was not required in our study as the zebrafish were assigned into different groups based on their genotype.
Blinding	Blinding was performed for single-cell profiling and analysis of the zebrafish crispant data.

Reporting for specific materials, systems and methods

We require information from authors about some types of materials, experimental systems and methods used in many studies. Here, indicate whether each material, system or method listed is relevant to your study. If you are not sure if a list item applies to your research, read the appropriate section before selecting a response.

Materials & experimental systems

n/a	Involved in the study	
☐	☒	Antibodies
☒	☐	Eukaryotic cell lines
☒	☐	Palaeontology and archaeology
☐	☒	Animals and other organisms
☒	☐	Human research participants
☒	☐	Clinical data
☒	☐	Dual use research of concern

Methods

n/a	Involved in the study	
☒	☐	ChIP-seq
☒	☐	Flow cytometry
☒	☐	MRI-based neuroimaging

Antibodies

Antibodies used	anti-DIG antibody (Roche #11093274910)
Validation	This is a broadly-used and validated antibody, as detailed here: https://www.sigmaaldrich.com/deepweb/assets/sigmaaldrich/product/documents/329/822/11093274910.pdf

Animals and other organisms

Policy information about studies involving animals; ARRIVE guidelines recommended for reporting animal research

Laboratory animals	Zebrafish, sex not specified. The following zebrafish lines were used in this study: AB* and floating headn1/n1 (flh/noto) mutants. Embryos were collected at 6-10 hpf.
Wild animals	No wild animals were used in the study.
Field-collected samples	No field-collected samples were used in the study.
Ethics oversight	Institutional Animal Care and Use Committees at Washington University in St. Louis

Note that full information on the approval of the study protocol must also be provided in the manuscript.
